# Systematic Review on the Association of Radiomics with Tumor Biological Endpoints

**DOI:** 10.3390/cancers13123015

**Published:** 2021-06-16

**Authors:** Agustina La Greca Saint-Esteven, Diem Vuong, Fabienne Tschanz, Janita E. van Timmeren, Riccardo Dal Bello, Verena Waller, Martin Pruschy, Matthias Guckenberger, Stephanie Tanadini-Lang

**Affiliations:** 1 Department of Radiation Oncology, University Hospital Zurich and University of Zurich, 8091 Zurich, Switzerland; diem.vuong@usz.ch (D.V.); Janita.vanTimmeren@usz.ch (J.E.v.T.); riccardo.dalbello@usz.ch (R.D.B.); matthias.guckenberger@usz.ch (M.G.); stephanie.tanadini-lang@usz.ch (S.T.-L.); 2 Laboratory of Applied Radiobiology, Department of Radiation Oncology, University of Zurich, 8091 Zurich, Switzerland; fabienne.tschanz@uzh.ch (F.T.); verena.waller@uzh.ch (V.W.); martin.pruschy@uzh.ch (M.P.)

**Keywords:** radiomics, tumor biology, cancer, imaging biomarker, tumor molecular marker

## Abstract

**Simple Summary:**

In this systematic review, we aim to highlight existing literature devoted to the study of an association between medical imaging radiomics and cancer biological endpoints. The use of radiomics as an ancillary tool in cancer treatment would allow for a non-invasive, inexpensive, three-dimensional characterization of the tumor phenotype, contributing to the delivery of precision medicine. Nonetheless, its clinical application remains a challenge, as extensive, multi-center validation studies of radiomic features connection with tumor biology are required. In this review, we performed a search in PubMed database for peer-reviewed studies which evaluate the association between radiomic features and the following set of clinically relevant tumor markers: anaplastic lymphoma kinase (ALK), v-raf murine sarcoma viral oncogene homolog B1 (BRAF), epidermal growth factor (EGFR), human epidermal growth factor receptor 2 (HER-2), isocitrate dehydrogenase (IDH), antigen Ki-67, kirsten rat sarcoma viral oncogene homolog (KRAS), programmed cell death ligand 1 (PD-L1), tumor protein p53 (TP-53) and vascular endothelial growth factor (VEGF).

**Abstract:**

Radiomics supposes an alternative non-invasive tumor characterization tool, which has experienced increased interest with the advent of more powerful computers and more sophisticated machine learning algorithms. Nonetheless, the incorporation of radiomics in cancer clinical-decision support systems still necessitates a thorough analysis of its relationship with tumor biology. Herein, we present a systematic review focusing on the clinical evidence of radiomics as a surrogate method for tumor molecular profile characterization. An extensive literature review was conducted in PubMed, including papers on radiomics and a selected set of clinically relevant and commonly used tumor molecular markers. We summarized our findings based on different cancer entities, additionally evaluating the effect of different modalities for the prediction of biomarkers at each tumor site. Results suggest the existence of an association between the studied biomarkers and radiomics from different modalities and different tumor sites, even though a larger number of multi-center studies are required to further validate the reported outcomes.

## 1. Introduction

Cancer precision medicine involves therapy adaptation to improve clinical outcome based on patient-specific characteristics as well as the tumor-specific molecular profile. The advent of high-throughput gene-sequencing techniques in the last decade has allowed for the identification of multiple tumor molecular markers, also known as signature molecules, which correspond to genomic changes that affect gene and protein expression [1,2]. These encompass a great variety of biological molecules such as nucleic acids, proteins, peptides, lipid metabolites and other small molecules, and their assessment can be beneficial for diagnosis, prognosis, or prediction of therapy response. Besides assisting in clinical-decision processes, these signature molecules may also hold the potential for new personalized molecular targeted or immunologic therapies.

On the other hand, the fast-evolving field of radiomics has experienced an increased interest in the past decade, especially within cancer research, due to accumulating evidence of an association between quantitative medical imaging features and clinical and biological endpoints [3]. The underlying principle behind radiomics is that medical images enclose latent information which can be unveiled through the extraction of radiomic features, i.e., quantitative features which describe the shape, size, intensity and texture of a region of interest. The most common imaging modalities used for this purpose are computed tomography (CT), magnetic resonance imaging (MRI), positron emission tomography (PET) and ultrasound (US). Recent advances in machine learning and computer hardware, together with the availability of large-scale medical imaging data, have redefined radiomics as a powerful tool for precision medicine in clinical-decision support systems [4]. Moreover, the non-invasiveness nature of radiomics supposes a great advantage when compared to current gold-standard techniques for tumor phenotype characterization.

The purpose of this systematic review was to determine which radiomic features have been linked to tumor biology in peer-reviewed studies and, thus, could be potentially incorporated in cancer precision medicine. To this end, we chose to summarize current findings assessing the potential association of radiomics with ten classic proteins or genes which are relevant clinical prognostic markers, and which may be targeted by either small molecular inhibitors or antibodies. These are: anaplastic lymphoma kinase (ALK), v-raf murine sarcoma viral oncogene homolog B1 (BRAF), epidermal growth factor (EGFR), human epidermal growth factor receptor 2 (HER-2), isocitrate dehydrogenase (IDH), antigen Ki-67, kirsten rat sarcoma viral oncogene homolog (KRAS), programmed cell death ligand 1 (PD-L1), tumor protein p53 (TP-53) and vascular endothelial growth factor (VEGF).

These factors, when mutated or over-expressed, play important roles in cancer progression and growth (EGFR, HER-2, KRAS, BRAF, ALK), angiogenesis (VEGF), cell cycle regulation and cell death (TP-53), the immune response (PD-L1), and metabolic regulation (IDH). At the same time, it is important to understand the proliferation kinetics (Ki-67 proliferation marker) of the respective cancer types as that can also influence the treatment response.

- EGFR, HER-2 and ALK are all receptor tyrosine kinases, located on the cell surface and activated through the binding of ligands, mostly growth factors. This leads to the activation of a whole range of downstream signaling cascades and results in cell survival, proliferation and migration [5,6].
- KRAS and BRAF are the genes responsible for making the proteins K-ras and B-raf, which are, amongst others, involved in important signaling pathways (e.g., Ras-Raf-MAPK, PI3-K-AKT) [7,8]. Mutation and down-/up-regulation of any of those kinases can lead to malignancy and especially cancer formation.
- VEGF is a signaling factor promoting the formation of new blood vessels. To grow and metastasize, solid cancers require blood supply, which they attain by expressing VEGF to form supporting vasculature [9].
- TP-53 is involved in the regulation and progression through the cell cycle; monitors genomic stability and can induce apoptosis. It is one of the most prominent tumor-suppressors [10].
- PD-L1 is involved in suppressing the adaptive arm of the immune system. By upregulating PD-L1 expression, cancer cells may evade the host immune system [11].
- IDH catalyzes the decarboxylation of isocitrate. Through this metabolic deregulation, cancer progression can be initiated or supported [12].
- Ki-67 is a protein that is present during all active phases of the cell cycle but absent in resting (quiescent) cells [13]. Therefore, this cellular proliferation marker is frequently used to distinguish fast growing cell populations, such has cancer cells, from normal cells.

Throughout this review, the term “biomarker” refers, for the sake of simplicity, to any of the above-mentioned biological endpoints. This is also in accordance with the World Health Organization (WHO), which defines biomarker as “any substance, structure, or process that can be measured in the body, or its products and influences or predicts the incidence of outcome or disease” [14].

## 2. Materials and Methods

The analysis was conducted according to the PRISMA-P Preferred Reporting Items for Systematic Reviews and Meta-Analyses statement [15]. The protocol for this systematic review was registered at PROSPERO (CRD42020207220) and is available at https://www.crd.york.ac.uk/prospero/display_record.php?ID=CRD42020207220 (accessed on 11 June 2021). No amendments were performed with respect to the published protocol.

### 2.1. Literature Search

The search was conducted in PubMed database. According to PRISMA guidelines, article selection was carried out via multiple steps. The literature search was performed using the query “Radiomics [All Fields] AND keyword [All Fields]”, where keyword corresponded to one of the ten molecular markers under study (i.e., ALK, BRAF, EGFR, HER-2, IDH, Ki-67, KRAS, TP-53, PD-L1 and VEGF) and the possible variations in its naming (e.g., HER2 and HER-2). The full list of queries is provided in the Supplementary Materials (List S1). In total, twenty independent searches were performed. No records were included from other sources such as direct correspondence with authors. The search had no start date limit and was concluded on 31 March 2020.

For each independent search, all retrieved studies were collected, and duplicates were posteriorly removed using the open-source reference management software Zotero [16].

### 2.2. Eligibility Criteria

During the first screening phase, those studies which did not fulfil the following requirements were excluded: (1) the article had to be written in English, (2) the study had to be a scientific article excluding reviews, (3) the topic had to be related to biomarkers in cancer. Following this step, every article was assigned to one of the following categories, depending on the cancer site: breast, central nervous system (CNS), gastrointestinal, liver, lung and others.

The full-text articles were then assessed for eligibility. An article was excluded from the final analysis if at least one of the following criteria applied: (1) only one of the two groups, biomarker-negative or biomarker-positive, patients were included in the study, (2) the total number of patients was less than 40, (3) the association between the biomarker and radiomics features was not investigated, (4) the biomarker analyzed was not among the ten biomarkers defined in the search and (5) less than 20 image features were investigated.

### 2.3. Analysis

Those articles that satisfied the screening and eligibility criteria were included in the following analysis, with each tumor site corresponding to a dedicated subsection in this review. First, the distribution of the number of patients included within all the studies was evaluated. The frequency of investigation of a given biomarker for each tumor site was collected in a dedicated table, together with the total number of studies on each tumor site and on each biomarker.

For each study, we gathered the following information when available: the studied biological endpoint and its alteration, e.g., mutation on a specific exon, over-expression, etc.; the imaging modality; the origin of the dataset; the training set size; the validation set size and type of validation, i.e., internal, temporally independent, external, leave-one-out-, 3-, 5- and 10-fold cross-validation (LOOCV, 3-CV, 5-CV, 10-CV) or bootstrap methods; the initial number of studied radiomic features; the application of feature reduction and feature robustness analysis methods; the reported performance, i.e., the area under the receiver-operating characteristic curve (AUC), classification accuracy or c-index; the public availability of the code and/or data; the reported quality score of the radiomics study, e.g., the transparent reporting of a multivariable prediction modelling for individual prognosis or diagnosis (TRIPOD) score [17] or Radiomics Quality Score (RQS) [4].

Furthermore, radiomic features of the best performing models on the training set were identified for each combination of tumor site, biomarker and image modality, in order to provide, when possible, a visual interpretation of the findings. For consistency, performance on the training set was evaluated since external validation was only performed on a small fraction of the studies. Moreover, in this comparison, the selection was limited to models based solely on radiomic features, i.e., mixed models including clinical-radiological data were excluded. This process was done independently by each of the authors in the systematic review. If the study provided a visual interpretation of such features, it was recorded. Otherwise, whenever possible, the missing interpretation was provided by the authors.

In accordance with PRISMA guidelines, a strategy for bias risk minimization was adopted as follows: the processes of screening, eligibility evaluation and extraction of data for the meta-analysis were performed independently by authors ALG, DV, FT, RDB and VW. Each author analyzed one specific tumor site. The more experienced authors JEvT, ST-L, MG and MP supervised the process and guaranteed a uniform and unbiased analysis throughout the different tumor sites. A detailed description of this process can be found on the PRISMA checklist in the Supplementary Materials (List S2).

## 3. Results

### 3.1. Literature Search, Eligibility Criteria and Study Selection

**Figure 1:**
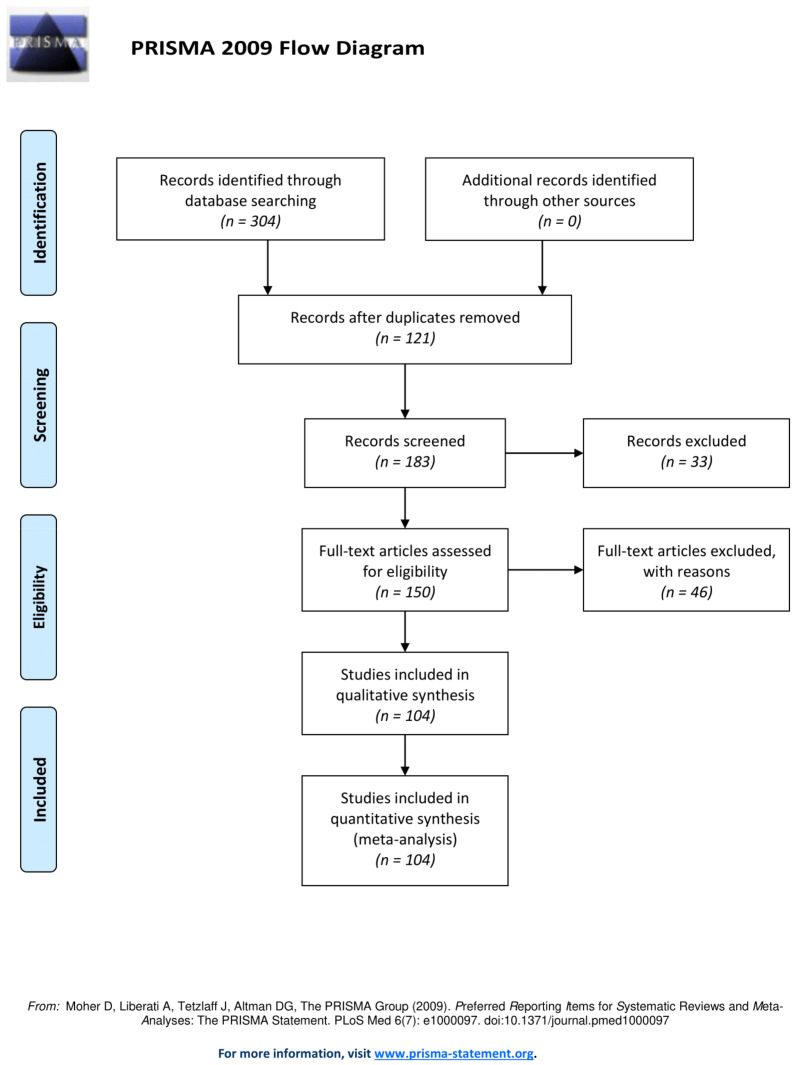
Flow diagram of the study selection process according to PRISMA guidelines [15].

A diagram summarizing the study selection workflow following PRISMA guidelines is shown in Figure 1. A total of 304 records were first retrieved from PubMed. After duplicate removal, 183 articles were left for screening. The first screening excluded 33 articles, leaving 150 full-text studies for the eligibility assessment. After further evaluation, 46 references were excluded because they did not meet the conditions previously defined. As a result, 104 articles were included in the current review.

**Figure 2:**
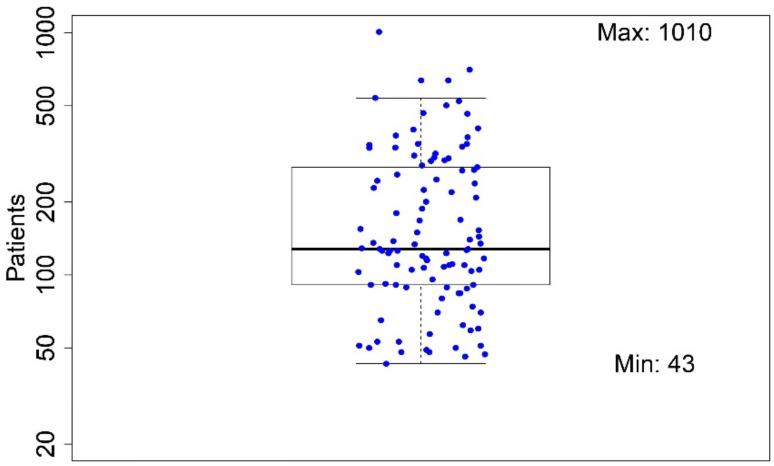
Distribution of the number of patients in the studies included in the analysis.

The size of the dataset under study varied significantly among the reported papers (43–1010 patients). As above-mentioned, studies including less than 40 patients in total were excluded from the analysis during the screening phase. The mean number of patients included was 198. The distribution is shown in Figure 2.

The frequency of investigation of a given biomarker with respect to each tumor site is presented in Table 1. It should be noted that multiple keywords, i.e., multiple biomarkers, were allowed for the same article. Therefore, the total sum of the entries (*n* = 125) is greater than the number of full-text papers included in the systematic review (*n* = 104). Similarly, those articles that investigated more than one tumor site were included in each of the corresponding subsections. The most frequently studied entity was lung cancer, followed by CNS tumors and breast cancer. The most frequently analyzed biomarker was EGFR, followed by Ki-67 and IDH. The association between EGFR and radiomics in lung cancer was the most frequently investigated (*n* = 26).

### 3.2. CNS

#### 3.2.1. Summary

A total of thirty-six studies were found which associated CNS tumor molecular markers with clinical imaging radiomic features, using conventional magnetic resonance imaging (MRI) alone (*n* = 21) [18,19,20,21,22,23,24,25,26,27,28,29,30,31,32,33,34,35,36,37,38], advanced MRI sequences (*n* = 11) [39,40,41,42,43,44,45,46,47,48,49], Positron Emission Tomography (PET, *n* = 3) [50,51,52] or Amide Proton Transfer-weighted (APTw, *n* = 1) imaging [53]. Glioma was the tumor type analyzed in the vast majority (*n* = 35) [18,19,20,21,22,23,24,25,26,27,28,29,30,31,32,33,34,36,37,38,39,40,41,42,43,44,45,46,47,48,49,50,51,52,53], including lower-grade glioma (LGG) (*n* = 20) [18,21,22,23,24,25,26,27,28,29,30,32,34,36,37,46,47,48,49,53], gliomas of all grades (*n* = 9) [33,38,42,43,44,45,50,51,52], and glioblastoma (GBM) [17,18,29,37,38,39]. One study focused on pituitary macroadenoma [35]. IDH genotype was the most frequently studied biomarker (*n* = 24) [18,19,20,21,22,23,24,25,26,27,28,29,30,38,42,43,45,46,47,48,49,50,52,53], followed by EGFR (*n* = 5) [31,32,39,40,41], Ki-67 (*n* = 5) [33,34,35,44,51], TP-53 (*n* = 2) [30,36] and VEGF (*n* = 1) [37]. All studies showed a significant association between the biomarker and radiomic features (AUC = 0.70–0.99). Thirty-four studies validated their models either on internal cohorts, temporally independent cohorts or through cross-validation/bootstrap methods. Two studies validated their models using externally acquired datasets. Three studies were prospective registered studies [21,47,53]. None of the studies reported any radiomics quality measure. The findings of this section are summarized in Table 2.

#### 3.2.2. IDH

A total of fifteen studies were found which investigated the power of MR radiomics to predict IDH genotype in glioma (AUC = 0.70–0.99, accuracy = 73.1–97%) [18,19,20,21,22,23,24,25,26,27,28,29,30,38]. Four studies exclusively employed MR radiomics [20,21,23,25], whereas eight studies used a combination of traditional radiomic features with other types of imaging features such as location parameters (*n* = 5) [23,24,25,26,27], Visually AcceSAble Rembrandt Images (VASARI) features (*n* = 3) [19,29,30], deep learning radiomics (DLR, *n* = 2) [22,27], and tumor growth model parameters (*n* = 1) [38]. Three studies based their models completely on non-conventional radiomics: one of them employed purely anatomical location features [24], another one used histogram of oriented gradients (HoGs), raw voxel intensities and scale-invariant feature transform (SIFT) descriptors [28] and the third one used fine texture features obtained by k-means singular value decomposition (K-SVD), a dictionary learning algorithm [18]. Three of the fifteen studies also incorporated clinical-radiological parameters into their models [19,27,29].

The best predictive performance was achieved by Li et al. on MR images of glioblastoma patients by means of a random forests (RF) model based on gray-level co-occurrence matrix (GLCM), grey-level run-length matrix (GLRLM) and grey-level size zone matrix (GLSZM) textural features together with patient age (AUC = 0.96, external validation) [19]. Among MR radiomics in both, lower- and higher-grade gliomas, relevant features for IDH mutation status prediction were associated with textural heterogeneity, suggesting that IDH wild-type tumors are more heterogeneous and more structurally complex than IDH-mutant ones [19,20,26]. Another feature that was significantly linked with IDH mutation status was tumor mean surface-to-volume ratio, which was lower in IDH-mutant cases [20,23]. Moreover, IDH-mutant gliomas were found to occur more frequently in the frontal, insular and temporal lobes [24,26].

A total of seven studies were found which combined MR radiomics with diffusion weighted imaging (DWI), perfusion weighted imaging (PWI) and/or diffusion kinetic imaging (DKI) features to predict IDH mutation status in glioma patients (AUC = 0.747–0.931) [42,43,45,46,47,48,49]. Among these studies, three of them incorporated clinical-radiological parameters in their modelling [42,43,45] and one employed additional VASARI imaging features [49]. The best performance on an external cohort was achieved by Lu et al., who proposed two support vector machine (SVM) models based on MR and DWI features together with patient age and sex to predict IDH mutation status in GBM and LGG patients separately (accuracy = 88.9–91.7%, external validation) [43]. Similar to MRI radiomics, DWI and PWI textural and intensity features describing increased tumor heterogeneity were associated with IDH wild-type tumors. Moreover, IDH wild-type LGGs were found to have smaller minimum Apparent Diffusion Coefficient (ADC) and Cerebral Blood Volume (CBV) values, which could indicate an increased tumor proliferation index and increased malignancy [47,49].

Two studies were found which used PET radiomics in conjunction with clinical-radiological parameters to predict IDH status in gliomas. One of them used fluoroethyl-tyrosine (FET)-PET standard parameter slope and one texture feature (accuracy = 80.0%, 10-CV) [52], while the other one combined fluorodeoxyglucose (FDG)-PET radiomics with age and tumor metabolism to achieve an AUC of 0.90 on an internal validation set [50]. Among FDG-PET radiomics, the feature sphericity was found to play a significant role in IDH mutation status prediction, indicating that IDH-mutant gliomas are less spherical than IDH wild-type in FDG-PET scans. Lastly, one study used APTw radiomics to predict IDH status in LGG patients (AUC = 0.84, internal validation) [53]. GLCM and GLRLM radiomic features describing tumor heterogeneity were identified as main contributors of IDH genotype prediction, with IDH-mutant tumors being more homogeneous.

#### 3.2.3. EGFR 

In total, two studies were found which investigated the relationship between MR radiomics and EGFR alterations in glioma, more precisely, between EGFR over-expression in LGG patients and EGFR mutation in GBM patients [31,36]. The former proposed a logistic regression model based on 41 radiomics features (AUC = 0.95, internal validation) [36], and the latter study employed a symbolic regression method based on non-conventional MR spatial diversity descriptors (AUC = 0.845, cross-validation) [31]. In both cases, MR features describing tumor textural heterogeneity and shape irregularity were linked to EGFR, suggesting increased diversity in EGFR-mutated and EGFR-amplified tumors.

Three studies were found which employed MR radiomics together with DWI and PWI radiomics to predict EGFR mutation status in GBM patients. Binder et al. studied a variety of EGFR missense mutations and concluded that EGFR mutation at alanine 289 (EGFR-A289D/T/V) presented a unique radiographic phenotype. Authors reported significantly lower average T1 signal, higher relative cerebral blood volume and longer major axis in EGFR-A289D/T/V-mutant tumors among other features [39]. The two remaining studies investigated the prediction of EGFR mutation at exons 2–7 (EGFRvIII) and incorporated additional imaging features on their modelling such as location parameters, tumor growth model parameters and the peritumoral heterogeneity index. Authors reported predictive accuracies of 73.58% [40] and 87% [41] on a temporally independent and on an internal validation cohort, respectively. Authors of the three above-mentioned studies suggested that EGFR-mutant tumors present an increase in shape variability and water concentration as well as a decreased cell density.

#### 3.2.4. Ki-67

Three studies were found which investigated the association between Ki-67 expression and MR radiomics in CNS tumors: one in lower grade glioma (AUC = 0.9, accuracy = 88.6%, internal validation) [34], one in both, lower and higher-grade gliomas (AUC = 0.713, accuracy = 66%, cross-validation) [33] and one in pituitary macroadenoma (accuracy = 96.7%, internal validation) [35]. In the three studies, Ki-67 expression was associated with increased textural heterogeneity. One study was found which combined MR, DWI and PWI radiomics to predict Ki-67 expression in grade I-IV gliomas (AUC = 0.936, training cohort) [44]. Authors reported that DWI features were more strongly associated with Ki-67 than the other two imaging modalities. Another study focused on Ki-67 prediction using FDG-PET radiomics (AUC = 0.76, internal validation) [51].

Among the five studies, different cut-off thresholds were employed to distinguish between low- and high-expressing tumors, with two studies using 10% [34,51], two studies using 25% [34,44] and one study using 3% [35].

#### 3.2.5. TP-53

Two studies evaluated the power of MR radiomics for TP-53 mutation status prediction in LGGs with a varying performance (AUC = 0.763–0.869, internal validation) [30,36]. One of them also included VASARI imaging features in the modelling. Authors concluded that TP-53 mutant gliomas are more heterogeneous and present higher water content. 

#### 3.2.6. VEGF

One study was found which investigated the use of conventional MR radiomics to predict VEGF expression in LGGs (AUC = 0.702, internal validation) [37].

### 3.3. Breast Cancer

#### 3.3.1. Summary

Sixteen studies which assessed the relationship between breast tumor molecular markers and radiomic features were identified. The imaging modalities studied were PWI (*n* = 9) [54,55,56,57,58,59,60,61,62], conventional MRI (*n* = 2) [63,64], DWI (*n* = 1) [65], DWI+PWI [66], Digital Breast Tomography (DBT, *n* = 1) [67], PET/CT (*n* = 1) [68] and standard 2D digital mammography (DMG, *n* = 1) [69]. Most studies focused on invasive breast cancer (*n* = 13) [54,55,56,57,58,59,60,63,64,65,66,67,69], while three studies investigated non-invasive breast cancer types [61,62,68]. HER-2 was the most frequently investigated biomarker (*n* = 10), and all studies showed some degree of linkage between HER-2 status and radiomic features (AUC = 0.65–0.95) [54,56,57,58,59,60,62,64,68,69]. Eight studies examined Ki-67, and all except one study found a significant association to radiomics features (AUC = 0.70–0.81) [56,57,62,63,65,66,67,68]. One study investigated TP-53 status (*n* = 1) (AUC = 0.88) [55]. Thirteen studies employed internal validation sets or cross-validation and bootstrap methods datasets. None of the studies reported any radiomics quality measure and only two of them were registered prospective studies [64,67]. The findings of this section are summarized in Table 3.

#### 3.3.2. HER-2

Ten studies focused on HER-2 status prediction based on radiomic features derived from PWI (*n* = 7) [54,57,58,59,60,61,62], DMG (*n* = 1) [69], PET/CT (*n* = 1) [68], and conventional MRI (*n* = 1) [64]. The term HER-2 positivity was used equivalently to an immunohistochemistry (IHC) result of +3 in most studies, while HER-2 negativity corresponded to an IHC score of 0 or +1.

Among PWI radiomics analysis, the highest predictive performance was achieved by Fan et al. by means of a logistic regression model based on 15 features from dynamic contrast enhanced MRI (DCE-MRI) images (AUC = 0.95, internal validation) [59]. Conventional MR features were also shown to associate with HER-2 status in [64] (accuracy of 73.6%, training cohort). In the study using PET/CT, only mean standardized uptake value (SUV_mean_) and total lesion glycolysis (TLG) were independently associated with HER-2 status (*p* = 0.021 and *p* = 0.046, respectively) [68]. DMG radiomic features were employed to predict HER-2 status in [69]. Authors reported higher prediction performance when employing a combination of bilateral craniocaudal and mediolateral oblique view images derived from 2D MG (AUC = 0.787, internal validation), compared to radiomic features from each view alone.

#### 3.3.3. Ki-67

Three studies investigated the relationship between Ki-67 expression levels and PWI radiomics (AUC = 0.74–0.81) [56,57,62], with two of them employing pharmacokinetic radiomic features. Authors in two of the three studies suggested that high Ki-67 expressing tumors are associated with higher intra-tumoral heterogeneity. On the other hand, DWI radiomics were employed in [65], achieving a final AUC of 0.72 on an internal validation cohort. Another study combined both, PWI and DWI radiomics, to predict Ki-67 status and reported a final AUC of 0.81 after cross-validation by means of a multi-task learning model which was also trained to predict tumor grade [66].

The study using PET/CT could not find any radiomic features that were significantly associated with Ki-67 expression level [68]. Another study used DBT images and showed that a combination of the five most predictive features yielded an AUC of 0.698 for low- versus high Ki-67 expression [67]. Liang et al. compared the utility of T1-weighted with contrast (T1 + C) to T2-weighted (T2w) radiomics to predict Ki-67 [63]. The analysis revealed that the T2w image-based radiomics classifier could significantly associate to Ki-67 expression on an external cohort (AUC: 0.740 (95% CI: 0.645–0.836), whereas T1 + C-based radiomics failed for the same dataset.

Among the eight studies exploring an association of Ki-67 expression levels with breast cancer radiomics, four studies employed a cut-off threshold of 14% [56,63,65,67], two studies used a threshold of 20% [62,68] and the other two did not specify any cut-off value.

#### 3.3.4. TP-53

The strongest association for breast cancer was found in PWI radiomics of 88 patients in which 13 radiomic features predicted TP-53 alterations with an AUC of 0.886 (95% CI: 0.817–0.955) after cross-validation [55].

### 3.4. Lung Cancer

#### 3.4.1. Summary

In total, 33 studies investigating radiomics and biological endpoints for lung lesions were identified. The imaging modalities employed were CT (*n* = 22) [70,71,72,73,74,75,76,77,78,79,80,81,82,83,84,85,86,87,88,89,90,91], PET/CT (*n* = 8) [92,93,94,95,96,97,98,99], PET (*n* = 2) [100,101] and MRI (*n* = 1) [102]. One study investigated radiomics from metastases, while all other studies associated tissue biomarkers with radiomics of the primary tumor. Almost half of the studies selected histological subtypes and used adenocarcinoma patients only (*n* = 15), whereas the other half used a mix of histologies. Research has predominantly focused on EGFR (*n* = 26) [70,71,72,73,74,75,76,77,78,79,80,81,82,83,84,85,86,92,93,94,95,96,97,100,101,102], followed by KRAS (*n* = 5) [83,97,100,101,102], ALK (*n* = 3) [87,98,102], PD-L1 (*n* = 3) [88,89,99], Ki-67 (*n* = 2) [90,91], and TP-53 (*n* = 1) [84]. All studies showed a significant relationship between EGFR and radiomic features (AUC = 0.66–0.95). Two studies that could not find an association with KRAS status, but all remaining studies found some linkage between radiomics and the respective biomarker (AUC = 0.66–0.99). In total, 21 studies validated their predictive models, two of which were external validation. None of the studies reported any radiomics quality measure, and only one of them was a registered prospective study [83]. A summary of the findings of this section can be found in Table 4.

#### 3.4.2. EGFR

The power of CT radiomics to predict EGFR mutations status strongly varied among the studies using validation (AUC = 0.69–0.851) [72,73,74,75,76,78,79,80,81,82,83,84,85,86]. Across CT radiomics studies, relevant features for EGFR mutation status prediction were associated with texture heterogeneity, suggesting that mutated tumors were more heterogeneous. Further, two studies observed an association of CT radiomics with EGFR mutation subtypes, i.e., differentiation subtype to wildtype (AUC = 0.655–0.727) [76,77] and within subtypes (AUC = 0.708–0.87) [81,95]. While PET radiomics also showed potential for EGFR mutation status prediction (AUC = 0.67, internal validation) [100,101], combining CT and PET were reported similar or better compared to single modal radiomics models [92,93,94,95,96]. Two studies examined radiomics at different time points other than pre-treatment [70,71]. In contrast to three-week post-treatment CT radiomics, one delta radiomic feature (i.e., change of feature value over time) was found predictive for EGFR mutation status (AUC  =  0.74, weakly corrected to delta volume and diameter) [70]. Multiple studies were found using contrast-enhanced CT (CE CT) [74,84,85], non-CE CT imaging [70,72,73,77,78,79,81,82] or a mix of thereof [75,83]. In a recent study, it was shown that a model based on CE CT features did not significantly performed different to a model based non-CE CT for EGFR mutation status prediction [85].

#### 3.4.3. KRAS

CT radiomics was weakly predictive for KRAS mutation status in 763 lung adenocarcinoma patients from four institutions (AUC = 0.63, temporally independent validation) [83]. Authors suggested that KRAS mutant tumors were more homogeneous. PET radiomics was reported non-predictive for KRAS mutation status (AUC < 0.56, no validation) [100,101]. Radiomics was further shown to better differentiate between EGFR and KRAS mutated tumors in CT (AUC = 0.80, internal validation) [83] than in PET (AUC = 0.65) [100].

#### 3.4.4. ALK

For ALK mutation status, CT radiomics showed an AUC of 0.80 on a temporally independent validation cohort [87]. Selected radiomic features inferred that ALK mutated tumors were associated with denser tumors. One study observed that PET-based radiomics combined with tumor stage and age was able to differentiate ALK/ROS1/RET fusion-positive and fusion negative tumors (sensitivity = 0.73, specificity = 0.70) [98]. A study on 110 patients evaluated if MR radiomics from brain metastasis originated from lung cancer was shown to associate with EGFR, ALK and KRAS mutations and reported excellent model performances for all three tissue biomarkers (AUC > 0.9, LOOCV) [102].

#### 3.4.5. PD-L1

PD-L1 expression levels were observed to associate with CT radiomic features in two studies (AUC = 0.661 [89] and AUC = 0.848 [88], internal validations), indicating that dense and homogeneous tumors (without ground-glass opacity, necrosis, cavitation or calcification) were more likely PD-L1 positive in lung adenocarcinoma [89]. Radiomics from PET/CT imaging was found to be similarly strongly predictive as CT but outperformed PET in PD-L1 expression level prediction for 399 stage I-IV non-small cell lung cancer (NSCLC) patients (AUC > 0.8, internal validation) [99].

#### 3.4.6. Ki-67

CT radiomic features were found significantly predictive for Ki-67 in two studies (best performing feature: inverse variance, AUC = 0.77) [90,91].

#### 3.4.7. TP-53

The association between CT radiomic features and TP-53 mutation was studied in [84]. Authors reported a final AUC of 0.604 on an internal validation cohort.

### 3.5. Gastrointestinal Cancer

#### 3.5.1. Summary

A total of ten studies addressing the relationship of radiomics and biological tissue markers in gastrointestinal cancers were identified. The imaging modalities used were CT (*n* = 6) [103,104,105,106,107], combined PET/CT (*n* = 2) [108,109], MRI (*n* = 2) [110,111] and MRI + PWI + DWI (*n* = 1) [112]. The tumor types analyzed belong to gastric cancers (*n* = 2) [103,104], rectal cancers (*n* = 3) [110,111,112], pancreatic cancers (*n* = 2) [105,108], colorectal cancer (CRC) and colorectal liver metastases (*n* = 3) [106,107,109]. The most frequent biomarker analyzed was KRAS mutation (*n* = 7) [106,107,108,109,110,111,112] followed by TP-53 mutation (*n* = 2) [108,109], Ki-67 (*n* = 3) [104,105,112] and HER-2 expression status (*n* = 2) [103,112]. One group studied BRAF (*n* = 1) together with KRAS and NRAS as one mutation signature [106]. All except one study on TP-53 showed a significant correlation between the respective biomarker and radiomic features (AUC = 0.65–0.88). All studies were set up retrospectively and used internal data. Two research groups validated their results on external datasets. None of the studies reported any radiomics quality measure and none of them were registered prospective studies. A summary of the findings of this section can be found in Table 5.

#### 3.5.2. KRAS

The association of KRAS mutations with radiomic signatures was the most frequently assessed in gastrointestinal cancer. The strongest relationship was found in CE CT of CRC patients, where the mutation signature KRAS/BRAF/NRAS was significantly associated with three GLCM features (energy, maximum probability and sum average), achieving a final AUC of 0.829 on an internal validation cohort [106].

One group focused on the association of KRAS mutation to FDG-PET radiomics of pancreatic ductal adenocarcinoma patients [108], concluding that low-intensity textural features were significantly associated with KRAS gene mutational status (AUC = 0.794–0.82, training). Authors suggested that KRAS-mutated genes were associated with higher intra-tumoral heterogeneity levels. The relationship between FDG-PET radiomics and KRAS mutation was also studied for CRC patients in [109]. KRAS-mutated tumors presented an increased value at the 25th percentile of maximal SUV (SUV_max_) of the metabolic tumor volume (MTV) as well as for the GLCM-derived contrast (AUC = 0.73–0.79, training).

Another study evaluated the association between KRAS mutation and CT imaging features, including hand-crafted and deep learning radiomics, of CRC patients [107]. The combined model achieved the highest performance (c-index = 0.831 (95% CI, 0.762–0.905), external validation), when compared to radiomics-alone and deep learning radiomics-alone models.

Two studies evaluated the association between T2w MR radiomics and KRAS mutational status in rectal cancer. In the first one, authors reported a final AUC of 0.884 on the training cohort by means of a decision tree based on three textural features [111]. In the second study, seven features were shown to associate to KRAS mutation status [110]. The best prediction model was obtained with SVM classifiers (AUC = 0.714 (95% CI, 0.602–0.827), external validation). Moreover, wavelet features derived from MR, PWI and DWI were associated with KRAS mutation in rectal cancer patients in [112], achieving a final AUC of 0.651 (95% CI, 0.539–0.763) on a temporally independent validation cohort. 

#### 3.5.3. TP-53

One group found that an increased value of short-run low gray-level emphasis derived from the GLRLM in FDG-PET/CT was predictive for TP-53 mutation in CRC patients (AUC = 0.71, training). Authors also reported higher heterogeneity and lower PET signal values in TP-53-mutant cases [109]. On the other hand, one study carried out with FDG-PET/CT data from pancreatic ductal adenocarcinoma patients did not see a significant association between genetic alterations in TP-53 status and the radiomic features extracted from the PET images [108].

#### 3.5.4. HER-2

The association of HER-2 status and CT radiomics in gastric cancer patients was investigated in [103]. Authors reported a final AUC of 0.771 (95% CI, 0.607–0.934) on an internal validation cohort when employing a nomogram based on seven wavelet features and patient carcino-embryogenic antigen (CEA) level. One study extracted radiomic features from pre-operative MR images of patients suffering from rectal cancer, achieving a final AUC of 0.696 (95% CI, 0.610–0.782) on a temporally independent validation cohort [112].

#### 3.5.5. Ki-67

Three studies investigated the potential association of Ki-67 index and radiomic signatures [104,105,112]. A CE CT-based radiomics nomogram including six radiomic features for the gastrointestinal stromal tumors was significantly associated with Ki-67 (AUC = 0.754, external validation) [104]. Another retrospective, multicenter study in CE CT focused on pancreatic neuroendocrine tumors showed a significant association between Ki-67 and an eight-feature-combined radiomics [105]. The third study analyzed a combination of MR, PWI and DWI radiomics to predict Ki-67 expression, with a final AUC of 0.699 on a temporally independent cohort [112]. Different Ki-67 expression cut-off values were used on each study, ranging from 10 to 40%.

#### 3.5.6. BRAF

As explained in the KRAS biomarker subsection, one study investigated the relation between CE CT radiomics and the mutation signature KRAS/NRAS/BRAF together, which reported a final AUC of 0.829 on a temporally independent cohort [106].

### 3.6. Liver Cancer

#### 3.6.1. Summary

Four studies were found which associated radiomics and tissue biomarkers in liver cancer patients, using either MR with contrast agents (*n* = 2) or US (*n* = 2). The most common tumor type was hepatocellular carcinoma (HCC, *n* = 3) [113,114,115] followed by cholangiocarcinoma (CCA, *n* = 1) [116]. Three tissue biomarkers were investigated: Ki-67 (*n* = 3), PD-L1 (*n* = 2) and VEGF (*n* = 1) and all were shown to be significantly correlated to radiomics (AUC = 0.85–0.97). All studies employed a dataset limited to a single center; one study separated the dataset into a training and a validation cohort [116]. None of the studies reported any radiomics quality measure and only one of them was a registered prospective study [114]. A summary of the findings of this section can be found in Table 6.

#### 3.6.2. PD-L1

The best predictive performance overall for liver studies was obtained for PD-L1 in US images of HCC patients (AUC = 0.97, cross-validation) [115]. The expression of PD-L1 was also predicted from MRI images of HCC, where the best association was found with the texture feature ADC variance. This may be interpreted as a correspondence between higher heterogeneity and higher PD-L1 expression levels [113].

#### 3.6.3. Ki-67

The best AUC for Ki-67 expression prediction in HCC was obtained in [115] by means of a SVM model based on US radiomic features (AUC = 0.94, cross-validation). Slightly worse performances (AUC = 0.804, internal validation) were obtained with US wavelet features for CCA patients in [116]. Another group employed texture features from MR images of HCC patients [114]. Authors combined 13 features from T2W, pre-contrast (PRE), arterial phase (AP) and portal venous phase (PV) scans into a multiparametric texture signature which achieve a c-index of 0.878 after cross-validation. The features included suggested that higher intra-tumor heterogeneity correlates to higher expression of Ki-67. The latter may reflect the cell proliferation status and therefore tumor aggressiveness.

#### 3.6.4. VEGF

The relationship between VEGF expression and US radiomic features was analyzed only in CCA patients [116]. Wavelet features were found to be the most relevant feature type to predict the biomarker expression (AUC = 0.864, internal validation). These were associated with the heterogeneity of the tumor volume by the authors.

### 3.7. Other Cancers

#### 3.7.1. Summary

In total, five studies were found which investigated the correlation between radiomics and molecular markers in other entities not included in the sections above: melanoma (*n* = 1) [117], thyroid cancer (*n* = 1) [118], head and neck cancer (*n* = 2) [119,120], adrenal gland carcinoma (*n* = 1) [121]. All studies showed a significant correlation between the biomarker and radiomics (AUC = 0.62–0.78). None of the studies used external validation. None of the studies reported any radiomics quality measure, nor were they registered prospective studies. A summary of the findings of this section can be found in Table 7.

#### 3.7.2. Details

One study explored the use of FDG-PET/CT radiomics to predict BRAFv600 mutation status in melanoma patients achieving a final AUC of 0.62 after 10-CV [117]. Another study investigated the use of US radiomics to predict BRAFv600 mutation of thyroid cancer patients with a limited predictive performance on a temporally independent validation cohort (c-statistics = 0.629) [118]. Two studies explored the association of different biomarkers and imaging features in head and neck squamous cell carcinoma patients. One of them reported a moderate predictive power of CT radiomics for TP-53 mutation prediction (AUC = 0.641, 5-CV) [119], while the other study reported a limited linkage between PD-L1, VEGF, Ki-67 and EGFR expression and FDG-PET radiomics on their training cohort [120]. The latter also showed a positive correlation between PD-L1 and Ki-67 expression. The GLCM-derived feature of correlation was found to be a negative predictor of PD-L1 expression, while it was positively associated with VEGF expression. One study investigated the efficacy of CE CT radiomics to predict Ki-67 expression in adrenal gland carcinoma patients [121]. The authors reported final AUCs of 0.7–0.78 on the training cohort after using logistic regression models based on two shape features, suggesting that high Ki-67 expression is associated with flatter and more elongated tumors.

### 3.8. Feature Interpretation

In Table 8, Table 9 and Table 10, we gathered those radiomic features employed in the best performing models for each combination of biomarker and tumor site, for MRI, CT and PET, respectively. Detailed tables including feature names and additional modalities (e.g., US or advanced MRI sequences) are shown in Supplementary Tables S1–S4. For seven studies, no interpretation was possible due to lack of information.

Oftentimes, dysregulation of one specific biomarker led to similar tumor phenotype across entities and imaging modalities. This was the case for EGFR-mutant tumors, which exhibited greater textural heterogeneity in CNS MRI, PWI and DWI, as well as in lung CT and PET. Similarly, alteration of TP-53 status was associated with increased heterogeneity in CT of HN and PET of colorectal cancer. IDH-mutant tumors were reported to have greater textural homogeneity in MRI, DWI, PWI, DKI and FDG-PET in CNS. High Ki-67-expressing tumors were reported to be more homogenous in CT for lung cancer but more heterogeneous for gynecological tumors and head and neck tumors. KRAS+ was shown to be more homogeneous for CT in lung, but more heterogeneous for gastrointestinal cancer.

### 3.9. Results per Biomarker

An overview of the analyzed studies per biomarker can be found in Tables S5–S14 in the Supplementary Materials.

## 4. Discussion

In recent decades, extensive genomic studies have leveraged our understanding of cancer biology and pathophysiology. The identification of key genetic alterations that drive oncogenesis and their subsequent molecular markers has led to a more accurate and comprehensive patient-specific treatment planning and adaptation [2]. Furthermore, the field of radiomics, i.e., the quantitative, high-throughput analysis of medical images, has emerged as a potential diagnostic, prognostic and predictive tool in clinical decision-support systems. This is of particular interest in cancer treatment, where medical imaging is routinely performed with diagnostic and monitoring purposes. Nonetheless, the reliability, clinical applicability and biological meaning of radiomics models and imaging biomarkers has to be extensively validated before they can be incorporated into clinical routine [17]. Hence, the primary objective of this review was to identify key radiomic features associated with specific tumor molecular markers through an electronic search of peer-reviewed journal publications.

For this purpose, we limited our search to ten cancer biological endpoints commonly investigated and used in clinical practice, which apply to a broad range of cancer types. Other, even though valid, biomarkers, such as methylation status or indicators for virus-born cancers were deliberately excluded as their origin and/or mechanism leading to malignant transformation of healthy cells is not trivially comparable. Other examples of biomarkers excluded in this review are the loss of tumor suppressors in cancer such as breast cancer genes 1 and 2 *(BRCA-1*, *BRCA-2*), RNAs, proteins such as prostate-specific antigen (PSA) or circulating tumor DNA (ct-DNA) [1]. By focusing on this compact set of biomarkers, we aimed to summarize the reported associations between radiomics and signature molecules and eventually contribute to the promotion of radiomics as a valid diagnostic, prognostic and predictive tool in cancer treatment. We are aware that the selection of biomarkers is not complete but due to the sheer number of biomarkers and the variability thereof, the search had to be narrowed in order to perform a meaningful systematic review.

Most of the studies included in this review reported some association between the selected biomarkers and radiomics, suggesting that mutated and non-mutated tumors have different growth patterns that are identifiable in high-throughput imaging. The association of textural, intensity, shape, size and wavelet image features with tumor biomarkers entails an advance in feature interpretability, as shown in Table 8, Table 9 and Table 10, which brings radiomics closer to its application in a clinical setting.

In total, 96 out of 104 studies found a significant relationship between at least one of the studied biomarkers and one or more radiomic features. However, only 7 studies validated their models on external cohorts, 11 studies on temporally independent cohorts and 14 studies did not use any form of validation. Additionally, only 7 out of 104 included a prospectively collected dataset, which is necessary to confirm the clinical validity and usefulness of any radiomics signature. Along these lines, we believe greater effort should be made to employ larger, multi-institutional cohorts, either by means of new data-sharing agreements among research groups or through distributed learning. The feasibility of the latter has already been shown in a number of studies and entails new possibilities for training reliable radiomics models [122,123]. Furthermore, only 37 studies performed some type of robustness analysis of the selected features. Different image acquisition parameters, scanner models, pre-processing and region of interest segmentation techniques among other factors have been shown to significantly affect feature robustness and results reproducibility, and should be evaluated in greater detail [124,125,126]. Moreover, we would like to encourage projects such as the image biomarker standardization initiative (IBSI) [127], which works towards the homogenization of image feature extraction and analysis.

Another factor that hindered results interpretation and studies comparison was the great variability in biomarker expression levels employed as cut-offs to stratify patients. Currently, there exist a lack of standardization of immunohistochemistry techniques for biomarker staining and scoring systems, leading to moderate intra/inter-laboratory and intra/inter-observer variabilities [1,128]. This could potentially explain the observed phenotype disagreement across different entities and modalities for Ki-67, PD-L1 and KRAS biomarkers, as described in Table 8, Table 9 and Table 10. However, as previously explained, it should be noted that these studies were included on the interpretation table based on their performance on the training set, and, for the vast majority, external validation remains to be accomplished.

In an attempt to standardize the clinical utility evaluation of radiomics studies, as well as to increase transparency and minimize risk of bias, two rigorous reporting guidelines, the TRIPOD [17] and the RQS [4] scores, have been devised. In Table 2, Table 3, Table 4, Table 5, Table 6 and Table 7, we gathered some of the most relevant reporting criteria such as the type of validation used, the performance of feature reduction and robustness analysis, the use of discrimination statistics, the inclusion of non-radiomic features and the public availability of the code and/or data. However, none of the studies included in this review followed explicitly TRIPOD or RQS guidelines. We would like to encourage the use of such guidelines as they provide a common framework to compare state-of-the-art results in radiomics and bring closer its incorporation into clinical decision support-systems.

## 5. Conclusions

In summary, radiomics from different modalities and cancer entities is a promising tool for tumor biology assessment. Nevertheless, a large majority of studies included in this review only employed internal validation datasets or bootstrap and cross-validation techniques to assess model performance. Thus, further multi-center, prospective studies are required to validate the reported outcomes. Moreover, none of the studies followed any reporting or quality assurance protocols. Hence, we would like to encourage the employment of reporting guidelines such as TRIPOD and RQS scores, as well as the use of IBSI-standardized radiomics software. As a closing remark, we would like to emphasize the utmost importance of transparency to ensure the reproducibility of radiomics studies.

## Figures and Tables

**Table 1:** Frequency of investigation of each biomarker and entity site.

	Breast	CNS	GI	Liver	Lung	Others	Total
ALK	0	0	0	0	3	0	3
BRAF	0	0	1	0	0	2	3
EGFR	0	5	0	0	26	1	32
HER-2	10	0	2	0	0	0	12
IDH	0	24	0	0	0	0	24
Ki-67	8	5	3	3	2	2	23
KRAS	0	0	7	0	5	0	12
PD-L1	0	0	0	2	3	1	6
TP-53	1	2	2	0	1	1	7
VEGF	0	1	0	1	0	1	3
TOTAL	19	37	15	6	40	8	125

**Table 2:** An overview of the radiomic studies included in the CNS cancer section.

Study	Biomarker	Alteration	Modality	Dataset Origin	Training	Validation	Feature Reduction	Feature Robustness	# Radiomic Features	Additional Features	Predictive Power Measure = Mean (95% Confidence Interval)	Open Source
Akbari et al. [41]	EGFR	Variant III mutation (deletion of exons 2–7)	MRI, DWI, PWI	Hospital of the University of Pennsylvania, Philadelphia, US	75	54 *	no	no	421	16 tumor spatial location features; peritumoral heterogeneity index	AUC = 0.92 Accuracy = 88.9%	Code
Arita et al. [26]	IDH	Isoforms 1 (codon 132) and 2 (codon 172) mutations	MRI	Osaka International Cancer Institute, Osaka, Japan; National Cancer Center Research Institute, Tokyo, Japan	111	58 *	yes	no	50	59 tumor spatial location features	Accuracy = 87%	Code Features
Binder et al. [39]	EGFR	Extracellular A289D/T/V, R108G/K and G598V mutations	MRI, PWI, DWI	Hospital of the University of Pennsylvania, Philadelphia, US	260	-	yes	no	2088	11 tumor spatial location features; 5 glioma diffusion properties from tumor biophysical models	Significant correlation (*p* < 0.0444)	Code
Choi et al. [20]	IDH	Isoforms 1 (codon 132) mutation	MRI	TCIA/TCGA-GBM; St. Mary’s Hospital, Seoul, South Korea	45	91 **	yes	no	107	-	AUC = 0.904 (0.805, 1.0) Accuracy = 86.8% (63.7, 97.8)	Images and ROI partially
Fukuma et al. [27]	IDH	Isoforms 1 (codon 132) and 2 (codon 172) mutations	MRI	Osaka International Cancer Institute, Osaka, Japan; National Cancer Center Research Institute, Tokyo, Japan	127	10-CV	yes	no	61	3 tumor spatial location features; 4000 DL features; age	Accuracy = 73.1%	-
Han et al. [53]	IDH	Isoforms 1 (codon 132) mutation	APTw	Tangdu Hospital, Xian, China	49	10 *	yes	yes	1044	-	AUC = 0.952 Accuracy = 0.892	Images on request
Kim et al. [47]	IDH	Isoforms 1 (codon 132) mutation	MRI, DWI, PWI	Asan Medical Center, Seoul, South Korea	127	28 ***	yes	yes	6472	-	AUC = 0.747 (0.66–0.83) Accuracy = 65.3%	-
Kong et al. [51]	Ki-67	High Ki-67 expression as > 10%	FDG-PET	Peking Union Medical College Hospital, Beijing, China	82	41 *	yes	no	1561	Age; sex; metabolic pattern; SUVmax; SUVmean	AUC = 0.73 Accuracy = 78%	-
Kuthuru et al. [28]	IDH	Isoforms 1 (codon 132) mutation	MRI	TCGA/TCIA-LGG	108	10-CV	no	no	No	> 35,000 histogram of oriented gradients, scale-invariant feature transform and voxel intensities	AUC = 0.8224 (0.7856–0.8575)	Images and ROI
Lee et al. [31]	EGFR	mutation	MRI	TCGA/TCIA-GBM	44	3-CV	no	no	-	36 spatial diversity features	AUC = 0.845 Accuracy = 0.79	Images and ROI
Lee et al. [48]	IDH	Isoforms 1 (codon 132) mutation	MRI, DWI, PWI	Samsung Medical Center, Seoul, South Korea	88	35 ***	yes	no	82	-	Accuracy = 83.4%	-
Li et al. [50]	IDH	Isoforms 1 (codon 132) and 2 (codon 172) mutations	FDG-PET	Peking Union Medical College Hospital, Beijing, China	84	43 *	yes	no	1561	Age; sex; metabolic pattern; SUVmax; SUVmean	AUC = 0.900 (0.877–0.923)	Code
Li et al. [19]	IDH	Isoforms 1 (codon 132) and 2 (codon 172) mutations	MRI	TCGA/TCIA-GBM; Sun Yat-sen University Cancer Center, Guangzhou, China; The 3rd Affiliated Hospital of Sun Yat-sen University, Guangzhou, China; Guangzhou General Hospital of Guangzhou Military Command, Guangzhou, China	118	107 **	yes	no	1614	Sex; age; KPS	AUC = 0.96 Accuracy = 97%	Images and ROI (partially)
Li et al. [33]	Ki-67	High Ki-67 expression as > 25%	MRI	The Second Hospital of Hebei Medical University, Tangshan, Hebei, China	50	3-CV, 5-CV, bootstrap	yes	no	396	-	AUC = 0.713 (0.568–0.832) Accuracy = 66.0%	-
Li et al. [32]	EGFR	High EGFR expression as > 30%	MRI	Beijing Tiantan Hospital, Beijing, China	200	70 *	yes	no	431	-	AUC = 0.95 Accuracy = 90.0%	
Li et al. [36]	TP-53	mutation	MRI	Chinese Glioma Genome Atlas, Beijing Tiantan Hospital, Beijing, China	180	92 *	yes	no	431	-	AUC = 0.763 Accuracy = 70.7%	Images and ROI
Li et al. [34]	Ki-67	High Ki-67 expression as > 10%	MRI	Beijing Tiantan Hospital, Beijing, China; Chinese Glioma Genome Atlas	78	39 *	yes	no	431	-	AUC = 0.90 Accuracy = 88.6%	-
Li et al. [22]	IDH	Isoforms 1 (codon 132) mutation	MRI	Huashan Hospital, Shangai, China	229	LOOCV	yes	no	671	16,384 DLR features	AUC = 0.9521 Accuracy = 92.44%	-
Liu et al. [21]	IDH	Isoforms 1 (codon 132) mutation	MRI	Beijing Tiantan Hospital, Beijing, China;	158	102 ***	yes	yes	431	-	AUC = 0.99	-
Lohmann et al. [52]	IDH	Isoforms 1 (codon 132) mutation	FET-PET	University Hospital RWTH Aachen	84	5-CV, 10-CV	no	yes	33	Slope; TTP; mean tumor-to-brain ratio; maximum tumor-to-brain ratio	AUC = 0.79 Accuracy = 80.0%	-
Lu et al. [43]	IDH	mutation	MRI, DWI	TCGA/TCIA-LGG; TCGA/TCIA-GBM; TCIA-REMBRANDT; Taipei Medical University, Taipei, Taiwan	214	70 **	yes	no	39,212	-	Accuracy = 88.9–91.7%	Images and ROI (partially)
Park et al. [46]	IDH	Isoforms 1 (codon 132) mutation	MRI, DWI	Yonsei University, Seoul, South Korea	168	10-CV	yes	no	411	-	AUC = 0.900 (0.855–0.945)	-
Rathore et al. [40]	EGFR	Variant III mutation (deletion of exons 2–7)	MRI, DWI, PWI	Hospital of the University of Pennsylvania, Philadelphia, US	107	10-CV	yes	no	255	9 tumor spatial location features; 3 biophysical growth model-based features	Accuracy = 80.19%	-
Ren et al. [49]	IDH	Isoforms 1 (codon 132) mutation	MRI, DWI, PWI	Huashan Hospital, Shangai, China	57	10-CV	yes	no	260	10 VASARI features; age; sex; Ki-67	AUC = 0.931 Accuracy = 94.74%	-
Su et al. [44]	Ki-67	High Ki-67 expression as > 25%	MRI, DWI, PWI	Tongji Hospital, Wuhan, Hubei, China	220	bootstrap	yes	no	431	-	AUC = 0.936	-
Sun et al. [37]	VEGF	VEGF expression at < 5%, 6–25%, 26–50% and > 50%	MRI	Beijing Tiantan Hospital, Beijing, China;	160	79 *	yes	no	431	-	AUC = 0.702 Accuracy = 72.3%	Images on request
Tan et al. [45]	IDH	mutation	DKI, DWI	Shanxi Medical University Shanxi, China	62	bootstrap	yes	no	728	Age; sex; grade; tumor size; tumor border; hemorrhage; cystic and necrosis; edema degree; enhancement style; enhancement degree; signal characteristics; 6 tumor location features; mean diffusivity value; mean kurtosis value	AUC = 0.885 (0.802–0.955) Accuracy = 80.6% (71.0–90.3)	-
Tan et al. [42]	IDH	Isoforms 1 (codon 132) and 2 (codon 172) mutations	MRI, DWI	Shanxi Medical University Shanxi, China	74	31 *	yes	yes	3882	Age; sex; grade; tumor size; tumor border; hemorrhage; cystic and necrosis; edema degree; enhancement style; enhancement degree; signal characteristics; 6 tumor location features	AUC = 0.900 (0.859–0.941) Accuracy = 87.1%	Features, ROI
Tontong Liu et al. [25]	IDH	Isoforms 1 (codon 132) mutation	MRI	Huashan Hospital, Shangai, China	110	LOOCV	yes	no	671	-	AUC = 0.90 Accuracy = 0.85	-
Ugga et al. [35]	Ki-67	High Ki-67 expression as > 3%	MRI	University of Naples “Federico II” Neurosurgery, Naples, Italy	53	36 *	yes	yes	1128	-	AUC = 0.87 Accuracy = 91.67%	-
Wu et al. [38]	IDH	Isoforms 1 (codon 132) mutation	MRI	TCGA/TCIA-LGG; TCGA/TCIA-GBM	126	bootstrap	yes	no	698	6 tumor growth model parameters	AUC = 0.931 Accuracy = 0.885	Images, ROI
Wu et al. [18]	IDH	Isoforms 1 (codon 132) mutation	MRI	Huashan Hospital, Shangai, China	80	25 *	yes	no	-	968 dictionary features	Accuracy = 88.0%	-
Yu et al. [24]	IDH	Isoforms 1 (codon 132) mutation	MRI	Huashan Hospital, Shangai, China	92	LOOCV	no	no	-	116 tumor spatial location features	AUC = 0.71 Accuracy = 72.0%	-
Yu et al. [23]	IDH	Isoforms 1 (codon 132) mutation	MRI	Huashan Hospital, Shangai, China	110	LOOCV	yes	no	671	-	AUC = 0.86 Accuracy = 80.0%	-
Zhang et al. [30]	IDH, TP-53	mutation	MRI	TCGA/TCIA-LGG	73	30 *	yes	yes	260	16 VASARI features	IDH: AUC = 0.792 Accuracy = 80.0% TP-53: AUC = 0.869 Accuracy = 85.0%	Images, ROI
Zhou et al. [29]	IDH	mutation	MRI	TCGA/TCIA-LGG	84	bootstrap	yes	no	3360	30 VASARI features; age; sex; KPS; histological type; grade; laterality; location	AUC = 0.86	Images, ROI, code

* internal validation; ** external validation; *** temporally independent internal validation. Acronyms: epidermal growth factor (EGFR), isocitrate dehydrogenase (IDH), antigen Ki-67 (Ki-67), tumor protein p53 (TP-53), vascular endothelial growth factor (VEGF), fluorodeoxyglucose positron emission tomography (FDG-PET), fluoroethyl tyrosine positron emission tomography (FET-PET), perfusion weighted imaging (PWI), magnetic resonance imaging (MRI), diffusion weighted imaging (DWI), amide proton transfer-weighted imaging (APTw), max and mean standardized uptake value (SUVmax, SUVmean), The Cancer Imaging Archive/The Cancer Genome Atlas (TCIA/TCGA), glioblastoma (GBM), lower-grade glioma (LGG), Karnofsky Performance Status (KPS), The Repository of Molecular Brain Neoplasia Data (REMBRANDT), Visually AccesSAble Rembrandt Images (VASARI), deep learning (DL), deep learning radiomics (DLR), time-to-peak (TTP), leave-one-out cross-validation (LOOCV), 3-, 5- and 10-fold cross-validation (3-, 5- and 10-CV), area under the curve (AUC).

**Table 3:** An overview of the radiomic studies included in the breast cancer section.

Study	Biomarker	Alteration	Modality	Dataset Origin	Training	Validation	Feature Reduction	Feature Robustness	# Radiomic Features	Additional Features	Predictive Power Measure = Mean (95% Confidence Interval)	Open Source
Antunovic et al. [68]	HER-2, Ki-67	HER-2: positive (IHC 3+) vs. negative (IHC 0 or 1) Ki-67: High expression at >20%	FDG-PET/CT	Humanitas Hospital, Milan, Italy	43	-	yes	no	20	MTV, SUV_mean_ and TLG	HER-2: Significant correlation (*p* = 0.021–0.046) Ki-67: No significant correlation	-
Braman et al. [61]	HER-2	mutation	PWI	Cleveland Medical Center, Cleveland, Ohio, US; City of Hope Comprehensive Cancer Center, Duarte, California, US; Yale Cancer Center, New Haven, Connecticut, US; Brown University Oncology Research Group, Providence, Rhode Island, US; TCIA/TCGA-BRCA	117	3-CV	yes	no	495	-	AUC = 0.71 (0.63–0.79)	images
Castaldo et al. [60]	HER-2	mutation	PWI	TCIA/TCGA-BRCA	55	36 *	no	no	36	**-**	AUC = 0.91 Accuracy = 81–88%	images
Fan et al. [59]	HER-2	positive (IHC 3+) vs. negative (IHC 0 or 1)	PWI	Zhejiang Cancer Hospital, Hangzhou, China	60	36 *	yes	no	65	Age, menopausal status; 29 dynamic features from BPE and the lesion; 9 bilateral differences in BPE	AUC = 0.947	-
Fan et al. [66]	Ki-67	mutation	PWI, DWI	First Affiliated Hospital of Zhejiang Chinese Medical University, Hangzhou, China	144	LOOCV	yes	no	97	-	AUC = 0.811	Code
Leithner et al. [64]	HER-2	mutation	MRI	Memorial Sloan Kettering Cancer Center, New York, USA; Medical University Vienna, Vienna, Austria	91	-	yes	no	352	-	Accuracy = 73.6%	Code
Li et al. [54]	HER-2	mutation	PWI	TCGA/TCIA-BRCA	91	LOOCV	yes	no	24	10 kinetic features (maximum contrast enhancement, TTP, uptake rate, washout rate, curve shape index, enhancement at first post-contrast, SER, volume of most enhancing voxels, total rate variation, normalized total rate variation) and 4 enhancement-variance kinetic features (maximum variance of enhancement, TTP, variance increase rate, and variance decrease rate)	AUC = 0.65	images
Li et al. [58]	HER-2	mutation	PWI	Cancer Hospital of Liaoning, China	637	LOOCV	yes	no	137	5 kinetic features (standard deviation, mean, maximum value, enhancement rate, absorption rate)	AUC = 0.83 Accuracy = 87.0%	-
Liang et al. [63]	Ki-67	High expression at >14%	MRI	Guangdong General Hospital, Guangdong Academy of Medical Sciences, Guangzhou, China & Southern Medical University, Guangzhou, Guangdong, China	200	118 ***	yes	yes	10,207	-	AUC = 0.740 (0.645,0.836) Accuracy = 0.729	-
Lin et al. [55]	TP-53	mutation	PWI	TCGA/TCIA-BRCA	88	LOOCV	yes	no	5234	-	AUC = 0.886 (0.817–0.955)	images
Ma et al. [56]	Ki-67	High expression at >14%	PWI	Tianjin Medical University Cancer Institute and Hospital, National Clinical Research Center for Cancer, Tianjin, China	159	10-CV	yes	no	56	-	AUC = 0.773 Accuracy = 0.757	-
Monti et al. [57]	HER-2, Ki-67	mutation	PWI	Hospital of Moscati, Avellino, Italy; Institute for Hospitalization and Healthcare ***SDN***, Naples, Italy	HER-2: 48 Ki-67: 49	bootstrap	yes	no	163	Pharmacokinetic maps	HER-2: AUC = 0.838 Accuracy = 0.785 Ki-67: AUC = 0.811 Accuracy = 0.677	
Tagliafico et al. [67]	Ki-67	High expression at >14%	DBT	Emergency Radiology, IRCCS Policlinico San Martino, Genova, Italy	70	bootstrap	yes	no	106	-	AUC = 0.698	Code, features
Zhang et al. [65]	Ki-67	High expression at >14%	DWI	The Second Hospital, Dalian Medical University, Dalian, China	101	27 *	yes	no	1029	-	AUC = 0.72 (0.495–0.857) Accuracy = 0.70	-
Zhou et al. [69]	HER-2	positive (IHC 3+) vs. negative (IHC 0 or 1)	DMG	Henan Provincial People’s Hospital, Henan, China	244	62 *	yes	no	186	-	AUC = 0.787 (0.673–0.885) Accuracy = 77.00%	
Zhou et al. [62]	HER-2; Ki-67	HER-2: positive (IHC 3+) vs. negative (IHC 0 or 1) Ki-67: High expression at >20%	PWI	The Affiliated Huaian No. 1 People’s Hospital of Nanjing Medical University, China	126	5-CV	yes	yes	386	-	HER-2: AUC = 0.68 Accuracy = 0.60 Ki-67: AUC = 0.74 Accuracy = 0.69	

* internal validation; *** temporally independent internal validation. Acronyms: human epidermal growth factor receptor 2 (HER-2), antigen Ki-67 (Ki-67), tumor protein p53 (TP-53), fluorodeoxyglucose positron emission tomography/computed tomography (FDG-PET/CT), perfusion weighted imaging (PWI), magnetic resonance imaging (MRI), diffusion weighted imaging (DWI), digital breast tomosynthesis (DBT), digital mammography (DMG), metabolic tumor volume (MTV), mean standardized uptake value (SUVmean), The Cancer Imaging Archive/The Cancer Genome Atlas (TCIA/TCGA), BReast invasive CArcinoma (BRCA), total lesion glycolysis (TLG), background parenchymal enhancement (BPE), signal enhancement ratio (SER), time-to-peak (TTP), leave-one-out cross-validation (LOOCV), 3-, 5- and 10-fold cross-validation (3-, 5- and 10-CV), area under the curve (AUC).

**Table 4:** An overview of the radiomic studies included in the lung cancer section.

Study	Biomarker	Alteration	Modality	Dataset Origin	Training	Validation	Feature Reduction	Feature Robustness	# Radiomic Features	Additional Features	Predictive Power Measure = Mean (95% Confidence Interval)	Open Source
Aerts et al. [70]	EGFR	Exons 19 and 21 mutations	CT	Memorial Sloan-Kettering Cancer Center, New York City, New York, US	47	-	yes	yes	183	-	AUC = 0.91	Images
Chen et al. [102]	EGFR; KRAS; ALK	mutation	MR	City of Hope Medical Center, Duarte, California, US	110	LOOCV	yes	yes	2786	Age; sex; ethnicity; history of smoking; histology type; other metastatic sites	EGFR: AUC = 0.912 Accuracy = 77.7% ALK: AUC = 0.915 Accuracy = 86.7% KRAS: AUC = 0.985 Accuracy = 96.7%	-
Gu et al. [91]	Ki-67	High Ki-67 expression as >50%	CT	The Third Xiangya Hospital of Central South University, Hunan, China	245	10-CV	yes	no	103	Lobulation sign; spicule sign; cavitation; cystic necrosis; pleural indentation; pleural effusion	AUC = 0.782	-
Hong et al. [85]	EGFR	Exons 18, 19, 20, and 21 mutations	CT	The First Hospital of China Medical University, Shenyang, China	140	61 *	yes	no	396	Age; sex; history of smoking	AUC = 0.851 (0.750–0.951) c-index = 0.835 (0.825–0.845)	-
Huang et al. [71]	EGFR	mutation	CT	The University of Texas MD Anderson Cancer Center, Houston, Texas	46	-	yes	yes	89	-	AUC = 0.88	Images
Jia et al. [72]	EGFR	Exons 19 and 21 mutations	CT	Shanghai Chest Hospital, Shanghai, China	345	158 *	no	no	440	Age; sex; smoking history; TNM stage	AUC = 0.828 (0.764–0.892)	-
Jiang et al. [99]	PD-L1	PD-L1 cutoff value of 1% and 50%	PET/CT	Shanghai Institute of Medical Imaging, Zhongshan Hospital of Fudan University, Shanghai, China	266	133 *	yes	no	1744	SUVmax; age; sex; smoking status; TNM stage; histology type	AUC = 0.97	-
Jiang et al. [92]	EGFR	mutation	PET/CT	Shanghai Institute of Medical Imaging, Zhongshan Hospital of Fudan University, Shanghai, China	80	10-CV	yes	no	512	12 semantic features	AUC = 0.953	-
Koyasu et al. [93]	EGFR	mutation	PET/CT	TCIA- NSCLC Radiogenomics	138	10-CV	yes	no	Not disclosed	SUVmax; SUVmean; TLG; MTV	AUC = 0.659 Accuracy = 81.2%	Images, ROI
Li et al. [94]	EGFR	Exons 18–24 mutations	PET/CT	Tianjin Medical University Cancer Hospital, Tianjin, China	115	10-CV	yes	no	38	SUVmax; SUVmean; SUVpeak; TLG; MTV; age; sex; smoking status; TNM stage; lesion location	AUC = 0.822 Accuracy = 82.65%	-
Li et al. [74]	EGFR	mutation	CT	Second Xiangya Hospital of Central South University, Hunan, China	51	10-CV	yes	yes	1695	-	AUC = 0.83 (0.68–0.92)	Images, ROI
Li et al. [75]	EGFR	Exon 19 and 21 mutations	CT	Shanghai Chest Hospital, Shanghai, China	810	200 *	yes	no	440	DL prediction; age; sex; smoking history; pathological stage	AUC = 0.834 (0.776–0.892)	-
Li et al. [76]	EGFR	Exon 19 and 21 mutations	CT	Shengjing Hospital of China Medical University, Liaoning, China	236	76 ***	yes	yes	580	Age; sex; tumor grade; lobe; smoking history; intrapulmonary metastasis	AUC = 0.7750–0.7925	-
Liu et al. [86]	EGFR	Exons 18–21 mutations	CT	Tianjin Medical University Cancer Institute and Hospital, Tianjin, China	298	bootstrap	yes	no	209	10 tumor spatial location features; age; sex; histological subtype; pathological stage; smoking history	AUC = 0.709 (0.654–0.766)	-
Lu et al. [73]	EGFR	mutation	CT	The First Hospital of Jilin University, China	83	21 *	yes	yes	1025	45 categorical variables including: age, sex, smoking status, CEA level, vascular infiltration, visceral pleural infiltration, lymph node metastasis, histological subtype, pathological stage, type of lesion, tumor location, tumor size, tumor necrosis, lobulation, spiculation, vacuolization, etc.	AUC = 0.894	code
Mei et al. [77]	EGFR	Exon 18–21 mutations	CT	Shenzhen People’s Hospital, Guangdong, China	296	-	yes	no	94	Age; sex; smoking status	AUC = 0.75	code
Nair et al. [95]	EGFR	Exons 19 and 21 mutations	PET/CT	McGill University Health Centre, 2011 and 2015	50	LOOCV	yes	no	326	-	AUC = 0.8713	-
Rios Velazquez et al. [83]	EGFR; KRAS	mutation	CT	Profile and Harvard-RT (Dana-Farber/Harvard Cancer Center IRB, Boston, MA), Tianjin (Tianjin Medical University IRB, Tianjin, China), Moffitt (IRB Moffitt Cancer Center, Tampa, FL)	353	352 ***	yes	yes	635	Age; sex; smoking status; ethnicity; clinical stage	EGFR: AUC = 0.75 (0.69–0.81) Accuracy = 65.0% KRAS: AUC = 0.75 (0.69–080) Accuracy = 66.0% EGFR+ vs. KRAS+: 0.86 (0.80–0.91) Accuracy = 79.0%	Images
Shiri et al. [97]	EGFR; KRAS	EGFR: Exons 18–21 mutations KRAS: Exon 2 codons 12 and 13 mutations	PET/CT	TCIA	82	68 *	yes	no	109	MTV, SUVmax, SUVpeak, SULmax, SULpeak	EGFR: AUC = 0.82 KRAS: AUC = 0.83	Images, ROI, code
Song et al. [87]	ALK	mutation	CT	Peking Union Medical College Hospital, Chinese Academy of Medical Sciences and Peking Union Medical College, November 2015 to October 2018	268	67 *	yes	no	1218	Age; sex; smoking history; smoking index; clinical stage; distal metastasis; pathological invasiveness of tumor; maximum diameter; mean CT attenuation; lesion location; involved lobe; density; margin; cavity; calcification; pleural retraction sign; pleural effusion; pericardial effusion; local lymphadenopathy	AUC = 0.88 (0.77–0.94) Accuracy = 79.0%	Images (partially), code
Sun et al. [88]	PD-L1	High PD-L1 expression as ≥ 50%	CT	The First Affiliated Hospital of Soochow University, Suzhou City, China	260	130 *	yes	yes	200	Age; sex; tumor location; CEA level; TNM stage; smoking status; histologic type; histologic grade	AUC = 0.848	-
Tu et al. [78]	EGFR	Exons 18–21 mutations	CT	Changzheng Hospital, Second Military Medical University, Shanghai, China	243	130 *	yes	yes	234	Age; sex; smoking status; CEA level; clinical stage; maximum diameter; density; tumor location; interface; shape; lobulation; pleural indentation; spiculation; cusp angle; spine-like process; vacuole sign; cavity sign; air bronchograms; vascular convergence; pleura thickening; pleural effusion; lymphadenopathy	AUC = 0.818 (0.751–0885) Accuracy = 75.8%	-
Wang et al. [84]	EGFR; TP-53	mutation	CT	Nanjing Medical University Affiliated Cancer Hospital, Nanjing, China	41	20 *	yes	no	718	78 clinical and pathological features (age, sex, smoking status, histological subtypes, pathological stages, etc.)	EGFR: AUC = 0.697 TP-53: AUC = 0.656	code
Yang et al. [79]	EGFR	Exons 18–21 mutations	CT	The First Affiliated Hospital of Guangzhou Medical University, Guangzhou, China	306	161 ***	yes	no	1063	Age; sex; smoking history; CT pattern; histopathological subtype	AUC = 0.779 (0.702–0.856)	code
Yip et al. [100]	EGFR; KRAS	EGFR: Exons 18–24 mutations KRAS: Exons 2–3 mutations	PET	Dana-Farber Cancer Institute, Brigham and Women’s Hospital, and Harvard Medical School, Boston, Massachusetts	348	bootstrap	yes	no	68	MTV, SUV_max_, SUV_peak_, SUV_mean_, and SUV_tot_	EGFR: AUC = 0.67 KRAS:- EGFR+ vs. KRAS+: AUC = 0.65	-
Yip et al. [101]	EGFR; KRAS	mutation	PET	Dana-Farber Cancer Institute, Brigham and Women’s Hospital, and Harvard Medical School, Boston, Massachusetts	348	-	yes	yes	66	-	EGFR: AUC = 0.66 KRAS:-	-
Yoon et al. [89]	PD-L1	High PD-L1 expression as ≥50%	CT	Severance Hospital, Yonsei University College of Medicine, Seoul, South Korea	153	bootstrap	yes	yes	58	Age; sex; smoking history; stage; tumor size; tumor location; tumor type; tumor margin; internal characteristics of tumor; external characteristics of tumor; lung metastasis; pleural effusion; pleural nodularity; pericardial effusion; lymphadenopathy	c-index = 0.646	-
Yoon et al. [98]	ALK/ROS1/RET	mutation	PET/CT	Samsung Medical Center, Sungkyunkwan University School of Medicine, Seoul, South Korea	128	10-CV	yes	yes	50	Age; sex; smoking history; stage; SUVmax; tumor solidity; tumor size; tumor location; lymphangitic metastasis; pleural effusion	Sensitivity = 0.73 Specificity = 0.70	-
Zhang et al. [96]	EGFR	Exons 18–21 mutations	PET/CT	The Fourth Hospital of Hebei Medical University, Hebei, China	175	73 *	yes	no	92	Age; sex; smoking history; pathological stage; CEA level	AUC = 0.87 (0.79–0.95)	-
Zhang et al. [80]	EGFR	Exons 18–21 mutations	CT	West China Hospital, Sichuan, China	140	40 *	yes	no	485	Age; sex; smoking status	AUC = 0.8725 Accuracy = 72.5%	-
Zhao et al. [82]	EGFR	Exons 18–21 mutations	CT	Huadong Hospital Affiliated to Fudan University, Shanghai, China; TCIA	464 nodules	115 nodules * 37 nodules **	yes	yes	475	DL prediction	AUC = 0.76	Images, ROI
Zhao et al. [81]	EGFR	Exon 19 and 21 mutations	CT	Second Xiangya Hospital, Central South University, Changsha, China; Huadong Hospital Affiliated to Fudan University, Shanghai, China	322	315 *	yes	yes	475	Age; sex; smoking status; tumor size; tumor location; histological subtype; TNM stage; tumor solidity; tumor margin; tumor type; pleural retraction; bubble lucency; vascular change; bronchiole change; lobulation; spiculation; peripheral emphysema; peripheral fibrosis; pleural effusion	AUC = 0.734	-
Zhou et al. [90]	Ki-67	High Ki-67 expression as > 40%	CT	Tianjin Medical University Cancer Institute and Hospital, Tianjin, China	110	-	yes	no	105	Age; sex; smoking history; histological subtype; TNM stage	AUC = 0.77	code

* internal validation; ** external validation; *** temporally independent internal validation. Acronyms: anaplastic lymphoma kinase (ALK), epidermal growth factor (EGFR), antigen Ki-67 (Ki-67), kirsten rat sarcoma viral oncogene homolog (KRAS), programmed cell death ligand 1 (PD-L1), computed tomography (CT), magnetic resonance imaging (MRI), positron emission tomography (PET), max, mean, peak and total standardized uptake value (SUVmax, SUVmean, SUVpeak, SUVtot), total lesion glycolysis (TLG), metabolic tumor volume (MTV), max and peak standardized uptake normalized to lean body mass (SULmax, SULpeak), carcino-embryogenic antigen (CEA), tumor, node and metastasis (TNM), non-small cell lung cancer (NSCLC), The Cancer Imaging Archive (TCIA), deep learning (DL), leave-one-out cross-validation (LOOCV), 3-, 5- and 10-fold cross-validation (3-, 5- and 10-CV), area under the curve (AUC).

**Table 5:** An overview of the radiomic studies included in the gastrointestinal cancer section.

Study	Biomarker	Alteration	Modality	Dataset Origin	Training	Validation	Feature Reduction	Feature Robustness	# Radiomic Features	Additional Features	Predictive Power Measure = Mean (95% Confidence Interval)	Open Source
Chen et al. [109]	KRAS; TP-53	KRAS: Exon 2 codons 12 and 13 mutation TP-53: Exons 2–11 mutations	FDG-PET/CT	China Medical University Hospital, Taichung, Taiwan	74	-	yes	no	56	SUVmax, SUVpeak, SUVtot, MTV, TLGmax, TLGpeak, and TLGmean	KRAS: AUC = 0.79 Accuracy = 77% TP-53: AUC = 0.71 Accuracy = 62%	-
Cui et al. [110]	KRAS	KRAS: Exons 2–4 mutations	MRI	Shanxi Province Cancer Hospital, Taiyuan, China; Xinhua Hospital, Shanghai, China	213	91 * 86 **	yes	no	960	**-**	AUC * = 0.682 (0.569–0.794) AUC ** = 0.714 (0.602–0.827)	-
Li et al. [103]	HER-2	positive (HER-2/CEP17 ≥ 2) vs. negative (HER-2/CEP17 < 2)	CT	Guangdong Provincial People’s Hospital, Guangzhou, China	94	40 *	yes	yes	12,410	CEA level	AUC = 0.771 (0.607−0.934)	-
Liang et al. [105]	Ki-67	mutation	CT	The First Affiliated Hospital, Hangzhou, Zhejiang, China; Second Affiliated Hospital, Hangzhou, Zhejiang, China	86	51 **	yes	no	467	Clinical stage	Significant correlation (*p* < 0.0001)	-
Lim et al. [108]	KRAS; TP-53	mutation	FDG-PET/CT	Samsung Medical Center, Sungkyunkwan University School of Medicine, Gangnam-gu, Seoul, South Korea	48	-	no	yes	27	SUVmax, SUVmean, SUVstd, SUVkurt, SUVskew, SUVent, MTV, TLG	KRAS: AUC = 0.829 TP-53:-	Code (partially)
Meng et al. [112]	Ki-67, KRAS, HER-2	KRAS: exon 2 codons 12 and 13 mutation Ki-67: High expression at >40% HER-2:	MRI, DWI, PWI	Sixth Affiliated Hospital of Sun Yat-sen University. Guangzhou, China	197	148 ***	yes	yes	2534	-	HER-2: AUC = 0.696 (0.610–0.782) Accuracy = 0.621 Ki-67: AUC = 0.699 (0.611–0.788) Accuracy = 0.582 KRAS: AUC = 0.651 (0.539–0.763) Accuracy = 0.616	-
Oh et al. [111]	KRAS	A59T, G12A, G12C, G12D, G12F, G12R, G12S, G12V, G13D, G61H, and Q61 mutation	MRI	Research Institute and Hospital, National Cancer Center, Goyang, Korea	60	-	no	no	44	-	AUC = 0.884 Accuracy = 81.7%	-
Wu et al. [107]	KRAS	Exons 2–4 mutations	CT	South China University of Technology, Guangzhou, Guangdong Province, China	279	119 ***	yes	yes	2634	2208 DL features	c-index = 0.832 (0.762–0.905)	-
Yang et al. [106]	KRAS; BRAF	KRAS: Exons 2–4 mutations BRAF: v600E mutation	CT	National Cancer Center/Cancer Hospital, Chinese Academy of Medical Sciences and Peking Union Medical College, Beijing, China	61	57 ***	yes	yes	346	-	AUC = 0.829 (0.718–0.939) Accuracy = 0.750 (0.623–0.845)	-
Zhang et al. [104]	Ki-67	High expression as ≥ 10%	CT	Renji Hospital, Huangpu, Shanghai, China; Zhongshan Hospital, Shanghai, China; Sir Run Shaw Hospital, Hangzhou, Zhejiang, China and First Affiliated Hospital of Wenzhou Medical University, Wenzhou, China	148	41 * 150 **	yes	yes	833	Tumor size	AUC * = 0.828 (0.681–0.974) AUC ** = 0.784 (0.701–0.868) Accuracy * = 68.29% Accuracy ** = 73.33%	Images/data on request

* internal validation; ** external validation; *** temporally independent internal validation. Acronyms: v-raf murine sarcoma viral oncogene homolog B1 (BRAF), antigen Ki-67 (Ki-67), kirsten rat sarcoma viral oncogene homolog (KRAS), tumor protein p53 (TP-53), fluorodeoxyglucose positron emission tomography (FDG-PET), computed tomography (CT), perfusion weighted imaging (PWI), magnetic resonance imaging (MRI), diffusion weighted imaging (DWI), carcinoembryonic antigen (CEA), metabolic tumor volume (MTV), max, mean, peak, standard deviation, skewness, kurtosis, entropy and total standardized uptake value (SUVmax, SUVmean, SUVpeak, SUVstd, SUVskew, SUVkurt, SUVent, SUVtot), max, peak and min of total lesion glycolysis (TLGmax, TLGmin, TLGpeak), deep learning (DL), area under the curve (AUC), chromosome enumeration probe 17 (CEP17).

**Table 6:** An overview of the radiomic studies included in the liver cancer section.

Study	Biomarker	Alteration	Modality	Dataset Origin	Training	Validation	Feature Reduction	Feature Robustness	# Radiomic Features	Additional Features	Predictive Power Measure = Mean (95% Confidence Interval)	Open Source
Hectors et al. [113]	PD-L1	expression	MRI, DWI	Icahn School of Medicine at Mount Sinai, New York, USA	48	-	no	no	196	Infiltrative pattern; presence of multiple lesions; extra-nodular growth; macrovascular invasion; tumor necrosis; tumor hemorrhage; tumor fat content; mosaic appearance; internal arteries; capsule; T2 hyper-intensity; ADC hypo-intensity; wash-in/wash-out; hepatobiliary phase hypo-intensity; ADCmin; ADCmean; ER in EA, LA, PV, LV and hepatobiliary phases; tumor size	Significant correlation (*p* < 0.029)	-
Peng et al. [116]	Ki-67; VEGF	Ki-67: High expression at ≥10% VEGF: expression	US	First Affiliated Hospital of Guangxi Medical University, Nanning, Guangxi, China	Ki-67: 63 VEGF: 39	Ki-67: 27 * VEGF: 18 *	yes	no	1,076	-	Ki-67: AUC = 0.848 Accuracy = 0.889 VEGF: AUC = 0.864 Accuracy = 0.833	-
Yao et al. [115]	Ki-67; PD-L1	Ki-67: High expression at ≥25% PD-L1: expression	US	Zhongshan Hospital, Fudan University, Shanghai, China	47	LOOCV	yes	no	-	2560 dictionary-based image features	PD-L1: AUC = 0.97 (0.89–0.98) Accuracy = 92% Ki-67: AUC = 0.94 (0.87–0.97) Accuracy = 93%	Images on request
Ye et al. [114]	Ki-67	High expression at ≥15%	MRI	West China Hospital, Sichuan, China	89	10-CV	yes	no	396	Serum level of alpha-fetoprotein; hepatitis B surface antigen; hepatitis C antibody; Barcelona-Clinic Liver Cancer classification; cirrhosis; multifocality; arterial phase hyper-enhancement; washout, capsule integrity, internal arteries, tumor margin, enhancing capsule, hepato-biliary phase hypo-intensity	c-index: 0.936 (0.863–0.977)	-

* internal validation. Acronyms: antigen Ki-67 (Ki-67), programmed cell death ligand 1 (PD-L1), vascular endothelial growth factor (VEGF), magnetic resonance imaging (MRI), diffusion weighted imaging (DWI), ultrasound (US), apparent diffusion coefficient (ADC), enhancement ratio (ER), early arterial (EA), late arterial (LA), early venous (EV), late venous (LV), portal vein (PV), area under the curve (AUC), leave-one-out cross-validation (LOOCV), 10-fold cross-validation (10-CV).

**Table 7:** An overview of the radiomic studies included in the other cancers section.

Study	Biomarker	Alteration	Modality	Dataset Origin	Training	Validation	Feature Reduction	Feature Robustness	# Radiomic Features	Additional Features	Predictive Power Measure = Mean (95% Confidence Interval)	Open Source
Ahmed et al. [121]	Ki-67	High expression at ≥10%	CT	MD Anderson Cancer Center, Texas, US	53	-	no	no	106	-	AUC = 0.78	-
Chen et al. [120]	PD-L1; EGFR; VEGF; Ki-67	PD-L1: High expression at ≥5% and ≥1% EGFR: expression VEGF: expression Ki-67: expression	FDG-PET	China Medical University, Taichung City, Taiwan	53	-	no	no	41	SUVmax, MTV, TLGmean; smoking history; tumor origin; TNM stage	PD-L1: AUC = 0.24 ^1^; EGFR: no correlation. VEGF: Correlation (*p* < 0.05); Ki-67: Correlation (*p* < 0.05)	-
Saadani et al. [117]	BRAF	v600E mutation	FDG-PET/CT	Netherlands Cancer Institute, Amsterdam, The Netherlands	70	10-CV	yes	no	480	SUVmax; SUVmean; SUVpeak; MTV; TLG; longest diameter	AUC = 0.62	-
Yoon et al. [118]	BRAF	v600E mutation	US	Severance Hospital, Yonsei University College of Medicine, Seoul, South Korea	387	140 ***	yes	no	730	Age; tumor size; sex;	AUC = 0.629 (0.516–0.742)	-
Zhu et al. [119]	TP-53	mutation	CT	TCIA/TCGA-HNSCC	126	5-CV	yes	yes	187	-	AUC = 0.641	Images, ROI

*** temporally independent internal validation; ^1^ negative correlation. Acronyms: v-raf murine sarcoma viral oncogene homolog B1 (BRAF), epidermal growth factor (EGFR), antigen Ki-67, kirsten rat sarcoma viral oncogene homolog (KRAS), programmed cell death ligand 1 (PD-L1), tumor protein p53 (TP-53), vascular endothelial growth factor (VEGF), computed tomography (CT), fluorodeoxyglucose positron emission tomography (FDG-PET), ultrasound (US), max, mean and peak standardized uptake value (SUVmax, SUVmean, SUVpeak), mean total lesion glycolysis (TLGmean), metabolic tumor volume (MTV), tumor, node and metastasis (TNM), 5- and 10- fold cross-validation (5-,10-CV), area under the curve (AUC).

**Table 8:** Interpretation of the best performing models on the training dataset for T2-weighted magnetic resonance imaging (MRI). Acronyms: central nervous system (CNS), gastrointestinal (GI), epidermal growth factor (EGFR), isocitrate dehydrogenase (IDH), kirsten rat sarcoma viral oncogene homolog (KRAS), tumor protein p53 (TP-53), vascular endothelial growth factor (VEGF).

MRI	EGFR	Ki-67	KRAS	TP-53	VEGF	IDH
CNS	EGFR+ more heterogeneous, less spherical [32]	Ki-67 high expression more heterogeneous [34]		TP-53+ higher intensity [36]	VEGF+ more heterogeneous [37]	IDH+ more homogeneous, more regularly shaped [21]
GI			KRAS+ more heterogeneous [110]			
Liver		Ki-67 high expression more heterogeneous [114]				

**Table 9:** Interpretation of the best performing models on the training dataset for computer tomography (CT). Acronyms: head and neck cancer (HNC), gastrointestinal (GI), anaplastic lymphoma kinase (ALK), v-raf murine sarcoma viral oncogene homolog B1 (BRAF), epidermal growth factor (EGFR), human epidermal growth factor receptor 2 (HER-2), kirsten rat sarcoma viral oncogene homolog (KRAS), programmed cell death ligand 1 (PD-L1), tumor protein p53 (TP-53).

CT	EGFR	Ki-67	KRAS/BRAF	TP-53	HER-2	ALK	PD-L1
HNC		Ki-67 high expression more heterogeneous [120]		TP-53+ more heterogeneous [119]			
Lung	EGFR+ more heterogeneous, smaller [83]	Ki-67 high expression more homogeneous, more elongated [90]	KRAS+ more homogeneous [83]			ALK+ higher density [87]	PD-L1+ more homogeneous [89]
GI		Ki-67 high expression more heterogeneous [104]	KRAS/BRAF+ more heterogeneous [106]		HER-2+ more heterogeneous [103]		

**Table 10:** Interpretation of the best performing models on the training dataset for positron emission tomography (PET). Acronyms: head and neck cancer (HNC), central nervous system (CNS), gastrointestinal (GI), epidermal growth factor (EGFR), isocitrate dehydrogenase (IDH), kirsten rat sarcoma viral oncogene homolog (KRAS), programmed cell death ligand 1 (PD-L1), tumor protein p53 (TP-53), vascular endothelial growth factor (VEGF).

PET	EGFR	Ki-67	KRAS	TP-53	VEGF	IDH	PD-L1
CNS						IDH+ more homogeneous, less spherical [50]	
HNC					VEGF+ more heterogeneous [120]		PD-L1+ more heterogeneous [120]
Lung	EGFR+ more heterogeneous, more compact [100]						
GI			KRAS+ lower intensity [108]	TP-53+ more heterogeneous [109]			
Adrenal gland carcinoma		Ki-67 high expression more elongated and flatter [121]					

## Supplementary Materials

The following are available online at https://www.mdpi.com/article/10.3390/cancers13123015/s1, List S1: Queries employed in the PubMed search, List S2. PRISMA Checklist, Table S1: Feature interpretation for ALK, BRAF and EGFR, Table S2: Feature interpretation for HER-2, IDH and Ki-67, Table S3: Feature interpretation for KRAS, KRAS/BRAF and PD-L1, Table S4: Feature interpretation for TP-53 and VEGF, Table S5: An overview of the radiomic studies included for IDH biomarker, Table S6: An overview of the radiomic studies included for EGFR biomarker, Table S7: An overview of the radiomic studies included for VEGF biomarker, Table S8: An overview of the radiomic studies included for HER-2 biomarker, Table S9: An overview of the radiomic studies included for ALK biomarker, Table S10: An overview of the radiomic studies included for BRAF biomarker, Table S11: An overview of the radiomic studies included for PD-L1 biomarker, Table S12: An overview of the radiomic studies included for TP-53 biomarker, Table S13: An overview of the radiomic studies included for KRAS biomarker and Table S14: An overview of the radiomic studies included for Ki-67 biomarker.
