# Supplementary material for: Systematic Review on the Association of Radiomics with Tumor Biological Endpoints
Source: Cancers (Basel). 2021 Jun 16;13(12):3015. doi: 10.3390/cancers13123015 (PMC8234501; doi:10.3390/cancers13123015)
Supplement: Supplementary file 1 [file cancers-13-03015-s001.zip › Supplementary_ListS1.pdf]

radiomics[All fields] AND EGFR[All fields]
radiomics[All fields] AND PD1[All fields]
radiomics[All fields] AND PD-1[All fields]
radiomics[All fields] AND PDL1[All fields]
radiomics[All fields] AND PD-L1[All fields]
radiomics[All fields] AND KRAS[All fields]
radiomics[All fields] AND P53[All fields]
radiomics[All fields] AND P-53[All fields]
radiomics[All fields] AND TP53[All fields]
radiomics[All fields] AND TP-53[All fields]
radiomics[All fields] AND IDH[All fields]
radiomics[All fields] AND IDH1[All fields]
radiomics[All fields] AND IDH-1[All fields]
radiomics[All fields] AND HER2[All fields]
radiomics[All fields] AND HER-2[All fields]
radiomics[All fields] AND ALK[All fields]
radiomics[All fields] AND VEGF[All fields]
radiomics[All fields] AND KI67[All fields]
radiomics[All fields] AND KI-67[All fields]
radiomics[All fields] AND BRAF[All fields]

List S1. Queries employed in the PubMed search.
