# Supplementary material for: Systematic Review on the Association of Radiomics with Tumor Biological Endpoints
Source: Cancers (Basel). 2021 Jun 16;13(12):3015. doi: 10.3390/cancers13123015 (PMC8234501; doi:10.3390/cancers13123015)
Supplement: Supplementary file 1 [file cancers-13-03015-s001.zip › Supplementary_TableS1, S2, S3 and S4.pdf]

Entity	*Imaging*	ALK		BRAF		EGFR	
		Feature	*Interpretation*	*Feature*	*Interpretation*	*Feature*	*Interpretation*
**CNS**	*MRI*					**T2W:** 25 first-order statistics or related wavelet features (range, SD, uniformity, variance, maximum, minimum, median, MAD, RMS, skewness), one shape- and size-based feature (spherical disproportion) and 15 textural features or related wavelet features (SRE, LGLRE, RLNU, maximum probability, SRHGLE, SRLGLE, SV, SE, RP) †[1]<br><br>**T1W + C, T2 FLAIR:** T1-high and T1-low Kendall index, mean and standard deviation of Fisher's alpha, skewness of the inverse Simpson [2] †	**T2W:** EGFR-amplified tumors have higher spherical disproportion and greater range and variance (greater heterogeneity) which may be linked to increased cell proliferation<br><br>**T1W + C, T2 FLAIR:** EGFR+ tumors show less spatial concordance among subregions and with respect to the rest of mutant individuals, increased spatial diversity and higher asymmetry, which may be linked to cell proliferation, angiogenesis and metastases<br><br>**T1W, T1W + C, T2W, T2 FLAIR, DWI, PWI:** EGFR+ tumors showed higher values for rCBV and FA, and lower values for ADC

						**T1W, T1W + C, T2W, T2 FLAIR, DWI, PWI:** SD of the non-ET intensity in T2W, 3rd and 4th bins of the non-ET intensity distribution in ADC maps and two atlas-based spatial distribution parameters of the tumor core [3]†	and T2-FLAIR, which may be linked to increased hypervascularity, cellularity, anisotropic tissue and decreased water concentration. EGFR+ tumors also presented a distinctive spatial pattern (more frequent in the frontal and parietal lobes)
**Lung**	*CT*	90th percentile, maximum, LHH_GLDM_LDHGLE†[4]	ALK+ tumors are more likely to have higher density			Homogeneity, inverse variance, HLL_GLCM_SE, LoG_SRLGLE, Volume [5] *	EGFR+ are smaller and more heterogeneous, potentially due to increased vascularization
	PET					Compactness, busyness, SUVmax, GLRLM_LGLRE, GLSZM_LGSZE, GLSZM_SZV, GLCM_IDM_normalized [6] †	EGFR+ are more compact and more heterogeneous
**Other**	*PET/CT*			**Melanoma**<br><br>GLCM_entropy, GLCM_joint_entropy, GLCM_IDM, GLCM_IDM_normalized, GLCM_DE, GLCM_SA, GLCM_SE, GLRLM_HGLRE,	**Melanoma**<br><br>BRAF+ more heterogeneous		

				GLRLM_SRE, GLSZM_ZP[7] †			
	US			**Thyroid Cancer**<br><br>MAD, SV, SE, HL_energy, LL_LRGLE, HL_RLNU, LL_uniformity, LL_SE [8]	**Thyroid Cancer**<br><br>Interpretation not possible		

Table S 1 Features of the best performing models on the training sets for the different modalities and tumor sites for biomarkers anaplastic lymphoma kinase (ALK), v-raf murine sarcoma viral oncogene homolog B1 (BRAF) and epidermal growth factor (EGFR). Acronyms: central nervous system (CNS), magnetic resonance imaging (MRI), computed tomography (CT), positron emission tomography (PET), ultrasound (US), T1-weighted (T1W), T2-weighted (T2W), plus contrast (+C), fluid attenuated inversion recovery (FLAIR), diffusion weighted imaging (DWI), perfusion weighted imaging (PWI), apparent diffusion coefficient (ADC), relative cerebral blood volume (rCBV), fractional anisotropy (FA), standard deviation (SD), mean absolute deviation (MAD), root mean square (RMS), short-run emphasis (SRE), gray-level co-occurrence matrix (GLCM), gray-level dependence matrix (GLDM), gray-level pre-emphasis matrix (GLPM), gray-level run length matrix (GLRLM), sum average (SA), sum variance (SV), sum entropy (SE), run percentage (RP), zone percentage (ZP), enhancement tissue (ET), low gray-level run emphasis (LGRLE), low gray-level short zone emphasis (LGSZE), run length non-uniformity (RLNU), high-gray level emphasis (HGLE), short-run high-gray level emphasis (SRHGLE), short-run low gray-level emphasis (SRLGLE), high gray-level run emphasis (HGLRE), low-pass filter (L), high-pass filter (H), laplacian of gaussian (LoG), large dependence high graylevel emphasis (LDHGLE), standardized uptake value (SUV), size zone variance (SZV), inverse difference moment (IDM), difference entropy (DE).

**Entity**	***Imaging***	**HER-2**		**IDH**		**KI-67**	
		Feature	*Interpretation*	*Feature*	*Interpretation*	*Feature*	*Interpretation*
**CNS**	*MRI*			**T2W:** 86 features comprising first order statistics features (energy, entropy, mean, median and RMS), surface-to-volume ratio, textural features (GLCM: contrast, dissimilarity, energy, entropy, DE, IMC; GLRLM: SRE, RP) and wavelet-derived features [9] †	**T2W:** IDH+ are more homogeneous and regular in shape with lower values for HHL_energy, HLL_DE, HLL_RP, HHL_variance, HLL_RMS and surface-to-volume ratio	**T2w**: 6 wavelet features derived from first-order statistics (energy, range, and maximum); 1 second-order feature (SD); and 2 wavelet features derived from textural features (correlation and HGLE) [13] †	**T2w**: Greater heterogeneity (greater range, HGLE and maximum values) in high Ki-67 expression tumors

				**T1W + C, T1 FLAIR, T2 BLADE, T2 FLAIR, PWI-CBF, DWI-ADC, DWI-eADC, PWI-ASL:** eADC_GLRLM_SRE, eADC_GLCM_energy, eADC_GLRLM_LRE, eADC_GLGCM_energy, eADC_GLRLM_RLNU, eADC_GLGCM_contrast, eADC_GLGCM_correlation, eADC_GLGCM_variance, eADC_hist_mode, eADC_hist_entropy, eADC_GLRLM_GLNU, eADC_GLSZM_GLNU, eADC_GLSZM_LGZE, eADC_GLCM_contrast, eADC_GLGCM_IDM, eADC_GLCM_correlation, ADC_hist_max, ADC_hist_min, ADC_NGTDM_coarseness, ADC_GLRLM_LGLRE, ADC_GLSZM_HGLZE CBF_GLSZM_SZE, CBF_GLSZM_ZSNU, FLAIR_GLSZM_LZE, FLAIR_GLRLM_RP, FLAIR_GLSZM_ZP, FLAIR_GLGCM_SA, FLAIR_hist_entropy [10] †	**T1W + C, T1 FLAIR, T2 BLADE, T2 FLAIR, DWI-CBF, DWI-ADC, DWI-eADC, PWI-ASL:** IDH+ more homogeneous with a coarser zone texture, lower energy, RLNU, variance and higher ADC minimum values, IDM, contrast, GLNU<br><br>**DWI, DKI:** IDH+ more homogeneous<br><br>**APTW:** Interpretation not possible	**T1 FLAIR, T1W + C, T2 FLAIR, T2 FSE, DWI-CBF, DWI-ADC, DWI-eADC, DWI-B0, DWI-B1000, PWI-CBF, PWI-ASL**:<br><br>Wavelet features derived from eADC_SD, eADC_MAD, eADC_ClusterTendency, eADC_homogeneity, eADC_InverseVariance, eADC_RP, B1000_MAD, B1000_energy, surface-to volume ratio, compactness, volume, spherical disproportion, ClusterProminence[14] †	**T1 FLAIR, T1W + C, T2 FLAIR, T2 FSE, DWI-CBF, DWI-ADC, DWI-eADC, DWI-B0, DWI-B1000, PWI-CBF, PWI-ASL**:<br><br>Interpretation not possible

				**DWI, DKI:** MK_90th, MD_elongation, MK_E5R5L5 [11] † <br>**APTW:** 13 GLCM features (correlation, entropy, inertia, haralick correlation, IDM, cluster prominence and energy) from different angles and offsets and 6 GLRLM features (RLNU, LGLRE, LRLGLE, HGLRE) from different angles and offsets [12] †			
	PET			**FET:** hist_skewness, LRHGLE, SZHGE[15] † **FDG:** sphericity, GLCM_ InverseVariance, GLDM_DNU_normalized, GLRLM_SRLGLE, GLCM_IMC, GLRLM_RLNU, HLL_GLCM_autocorrelation, LHL_skewness, lbp_RMS, lbp_mean [16] †	**FET:** Interpretation not possible **FDG:** IDH+ more homogeneous, less spherical	lbp_median, lbp_GLSZM_SALGLE, HHH_range, HLH_skewness, log_GLCM_difference variance, log_GLDM_SDHGLE, HLL_GLCM_correlation, grad_GLRLM_RE [17] †	Interpretation not possible
**Breast**	*MRI*	**PWI:** skewness, kurtosis, dynamic features in BPE and in breast lesions, two bilateral asymmetry features [18] †	**PWI:** HER-2+ tumors had the highest enhancement values in the normal breasts potentially			**PWI:** area, perimeter, effective diameter, GSS_kurtosis, GSS_entropy, GSS_skewness, GLCM_homogeneity,	**PWI:** High-Ki-67 tumors have higher values for area, perimeter, effective diameter, entropy, homogeneity, kurtosis

			linked to increased neo-angiogenesis, and higher skewness and kurtosis, potentially linked to higher heterogeneity and worse prognosis			GLCM_contrast, GLCM_IDM, GLDS_mean, GLDS_entropy, Tamura_coarseness, Tamura_line_likeliness [19] * <br>**DWI+PWI:** DE [20] *	and coarseness, and lower values for contrast, IDM, skewness line likeliness and GLDS_mean <br>**DWI+PWI:** DE is lower in PWI of high-Ki-67 expressing tumors, and higher in DWI of high-Ki-67 expressing tumors.
**GI**	*CT*	2 db wavelet-derived features (skewness, and GLSZM_LALGLE), 2 bior wavelet-derived features (GLDM_SDHGLE and kurtosis) and 3 rbio wavelet-derived features (GLCM_correlation, GLSZM_SALGLE, GLDM_DE) [21] †	HER-2+ more heterogeneous			HHH_GLSZM_LALGLE, HLL_GLCM_maxProbability, HHH_GLCM_IMC, LLL_NGTDM_Busyness, HLL_GLDM_LDE, LLL_firstorder_TotalEnergy [22] ‡	High-Ki-67 more heterogeneous
**Liver**	*MRI*					**T2W+PRE+AP+PVP:** multiparametric texture signature [23] *	High-Ki-67 tumors have higher heterogeneity, reflecting cell proliferation status, and aggressiveness
	US					elongation, LLH_lbp_skewness, LLH_lbp_kurtosis,	

						CoLIAGe2D_90Percentile	
**Lung**	*CT*					GLCM_InverseVariance, minor axis and elongation [24]*	High-Ki-67 more elongated and homogeneous
**Other**	*CT*					**HN**<br>HGLRE, SRHGLE, HGZE, SZLGE [25]†	**HN**<br>high expression of Ki-67 more heterogeneous
	PET					**Adrenal gland carcinoma**<br>shape elongation, shape flatness [26]†	**Adrenal gland carcinoma**<br>High expression of Ki-67 correlated with more elongated and flatter tumors

Table S 2 Features of the best performing models on the training sets for the different modalities and tumor sites for biomarkers human epidermal growth factor receptor 2 (HER-2), isocitrate dehydrogenase (IDH) and antigen Ki-67 (KI-67). Acronyms: central nervous system (CNS), magnetic resonance imaging (MRI), computed tomography (CT), positron emission tomography (PET), ultrasound (US), T1-weighted (T1W), T2-weighted (T2W), plus contrast (+C), fast spin echo (FSE), fluid attenuated inversion recovery (FLAIR), diffusion weighted imaging (DWI), perfusion weighted imaging (PWI), apparent diffusion coefficient (ADC), exponential apparent diffusion coefficient (eADC), relative cerebral blood volume (rCBV), cerebral blood flow (CBF), arterial spin label (ASL), fractional anisotropy (FA), standard deviation (SD), mean absolute deviation (MAD), root mean square (RMS), short-run emphasis (SRE), gray-level co-occurrence matrix (GLCM), gray-level dependence matrix (GLDM), gray-level gradient co-occurrence matrix (GLGCM), gray-level pre-emphasis matrix (GLPM), gray-level run length matrix (GLRLM), sum average (SA), sum variance (SV), sum entropy (SE), run percentage (RP), zone percentage (ZP), enhancement tissue (ET), low gray-level run emphasis (LGRLE), low gray-level short zone emphasis (LGSZE), low gray-level zone emphasis (LGZE), run length non-uniformity (RLNU), gray-level non-uniformity (GLNU), short-run high-gray level emphasis (SRHGLE), short-run low gray-level emphasis (SRLGLE), long run emphasis (LRE), high gray-level run emphasis (HGLRE), low gray-level run emphasis (LGLRE), long run low gray-level emphasis (LRLGLE), low-pass filter (L), high-pass filter (H), laplacian of gaussian (LoG), large dependence high graylevel emphasis (LDHGLE), standardized uptake value (SUV), size zone variance (SZV), inverse difference moment (IDM), difference entropy (DE).informational measure of correlation (IMC), histogram (hist), neighborhood gray-tone difference matrix (NGTDM), high gray-level zone emphasis (HGLZE), long zone emphasis (LZE), zone size non-uniformity (ZSNU), mean diffusivity (MD), mean kurtosis (MK), dependence non-uniformity (DNU), background parenchymal enhancement (BPE), grayscale statistic (GSS), gradient (grad), local binary pattern (lbp), small area low gray-level emphasis (SALGLE), small dependence high gray-level emphasis (SDHGLE), large dependence emphasis (LDE), Daubechies wavelet (db), biorthogonal wavelet (bior) , reverse biorthogonal wavelet (rbio), pre-contrast (PRE), arterial phase (AP), portal venous phase (PVP).

**Entity**	***Imaging***	**KRAS**		**KRAS/BRAF**		**PD-L1**	
		Feature	*Interpretation*	*Feature*	*Interpretation*	*Feature*	*Interpretation*

GI	*CT*			HLH_GLCM_energy, LHH_GLCM_maximum_probability, HHH_GLCM_SA [27] †	KRAS/BRAF+ more heterogeneous		
	PET	GLRLM_LILRE, GLSZE_LIZE, GLSZE_LILZE[28] *	KRAS+ have low-intensity features potentially linked to hypoxia				
	MRI	elongation, flatness, LoG_maximum, LoG_10^{th}_percentile, GLDM_DV, GLDM_DN _normalized, GLRLM_SRLGLE[29] ‡	KRAS+ are more heterogeneous, with increased signal peakedness and variance				
**Liver**	*MRI*					**T1w + C, DWI:** ADC_variance, enhancement ratios at portal and late venous phase, correlation of late arterial phase [30] *	**T1w + C, DWI:** PD-L1+ more heterogeneous in DWI and presented higher enhancement ratios
**Lung**	*CT*	SE, total energy [5]†	KRAS+ more homogeneous			GLCM_ASM, GLRLM_RV, GLRLM_RE, GLRLM_SRHGLE [31] †	PD-L1+ tumors are more homogeneous
	PET	No distinctive feature [6] *	Interpretation not possible				
**Other**	*PET*					**HN**	**HN** PD-L1+ tumors have lower textural

						GLRLM_GLNUr, GLRLM_RP, GLSZM_SZLGE, GLCM_correlation [25] *	coarseness and are more heterogeneous

Table S 3 Features of the best performing models on the training sets for the different modalities and tumor sites for biomarkers kirsten rat sarcoma viral oncogene homolog (KRAS), KRAS together with v-raf murine sarcoma viral oncogene homolog B1 (BRAF), and programmed cell death ligand 1 (PD-L1). **Acronyms: gastrointestinal (GI), head and neck (HN), magnetic resonance imaging (MRI), computed tomography (CT), positron emission tomography (PET), ultrasound (US), T1-weighted (T1W), T2-weighted (T2W), plus contrast (+C), fluid attenuated inversion recovery (FLAIR), diffusion weighted imaging (DWI), perfusion weighted imaging (PWI), apparent diffusion coefficient (ADC), relative cerebral blood volume (rCBV), fractional anisotropy (FA), standard deviation (SD), mean absolute deviation (MAD), root mean square (RMS), short-run emphasis (SRE), gray-level co-occurrence matrix (GLCM), gray-level dependence matrix (GLDM), gray-level pre-emphasis matrix (GLPM), gray-level run length matrix (GLRLM), sum average (SA), sum variance (SV), sum entropy (SE), run percentage (RP), zone percentage (ZP), enhancement tissue (ET), low gray-level run emphasis (LGRLE), low gray-level short zone emphasis (LGSZE), run length non-uniformity (RLNU), high-gray level emphasis (HGLE), short-run high-gray level emphasis (SRHGLE), short-run low gray-level emphasis (SRLGLE), high gray-level run emphasis (HGLRE), low-pass filter (L), high-pass filter (H), laplacian of gaussian (LoG), large dependence high graylevel emphasis (LDHGLE), standardized uptake value (SUV), size zone variance (SZV), inverse difference moment (IDM), difference entropy (DE), difference variance (DV), difference normalized (DN), run variance (RV), run entropy (RE).**

**Entity**	*Imaging*	**TP-53**		**VEGF**	
		Feature	*Feature*	*Feature*	*Interpretation*
**CNS**	*MRI*	**T2w:** spherical disproportion, SA and thirteen wavelet-derived features: maximum median, uniformity, autocorrelation, correlation, SE, RLNU, LRLGLE, maximum probability, RP [32] †	**T2w:** TP-53+ tumors have higher maximum and median values possibly reflecting water content differences due to increased micro-vascularity, as well as lower uniformity	**T2w:** Cluster Tendency_HLL, Entropy_LLL, LRLGLE_LHL, Minimum, SRHGLE_LLH, SRLGLE_LLL, SRLGLEs_LHH, SRLGLE_HLL, SRLGLE_HLH [33] †	VEGF+ more heterogeneous
**Breast**	*MRI*	**T1w, DCE:** 5 shearlet-derived features: GLRLM_LRE, GLRLM_LRLGLE, GLSZM_LAHGLE, GLCM_cluster_shade and GLSZM_SZNU_normalized; 3 features derived from wavelets	Interpretation not possible		

		and 3D lbp: HLH_RMS, HLL_kurtosis, HLH_variance; 2 features from wilbp and gLTCoPs1_hist [34]			
**GI**	*PET*	GLRLM_SRLGLE [35]*	TP-53+ tumors are more heterogeneous		
**Liver**	*US*			NGTDM_Contrast, LLH_lbp-3D_Entropy, LLH_lbp-3D_Maximum, LLH_lbp-3D_Minimum, CoLIAGe2D_Kurtosis [36] †	VEGF+ more heterogeneous
**Other**	*CT*	**HN** MAD, global maximum, SE, texture strength, and global entropy [37]	**HN** TP-53+ tumors are more heterogeneous		
	PET			**HN** GLCM_entropy, GLNUz, RLNU GLCM_contrast, HGLRE, SRHGLE, HGZE, SZHGE, dissimilarity[25] †	**HN** VEGF+ more heterogeneous

Table S 4 Features of the best performing models on the training sets for the different modalities and tumor sites for biomarkers tumor protein p53 (TP-53) and vascular endothelial growth factor (VEGF). Acronyms: central nervous system (CNS), gastrointestinal (GI), head and neck (HN), magnetic resonance imaging (MRI), computed tomography (CT), positron emission tomography (PET), ultrasound (US), T1-weighted (T1W), T2-weighted (T2W), plus contrast (+C), dynamic contrast enhancement (DCE), relative cerebral blood volume (rCBV), fractional anisotropy (FA), standard deviation (SD), mean absolute deviation (MAD), root mean square (RMS), short-run emphasis (SRE), gray-level co-occurrence matrix (GLCM), gray-level dependence matrix (GLDM), gray-level pre-emphasis matrix (GLPM), gray-level run length matrix (GLRLM), sum average (SA), sum variance (SV), sum entropy (SE), run percentage (RP), zone percentage (ZP), enhancement tissue (ET), low gray-level run emphasis (LGRLE), low gray-level short zone emphasis (LGSZE), run length non-uniformity (RLNU), high-gray level emphasis (HGLE), short-run high-gray level emphasis (SRHGLE), short-run low gray-level emphasis (SRLGLE), high gray-level run emphasis (HGLRE), low-pass filter (L), high-pass filter (H), laplacian of gaussian (LoG), large dependence high graylevel emphasis (LDHGLE), standardized uptake value (SUV), size zone variance (SZV), inverse difference moment (IDM), difference entropy (DE), difference variance (DV), difference normalized (DN), run variance (RV), run entropy (RE).

[1] Y. Li *et al.*, "MRI features can predict EGFR expression in lower grade gliomas: A voxel-based radiomic analysis," *Eur. Radiol.*, vol. 28, no. 1, pp. 356–362, Jan. 2018, doi: 10.1007/s00330-017-4964-z.

[2] J. Lee, S. Narang, J. J. Martinez, G. Rao, and A. Rao, "Associating spatial diversity features of radiologically defined tumor habitats with epidermal growth factor receptor driver status and 12-month survival in glioblastoma: methods and preliminary investigation," *J. Med. Imaging Bellingham Wash*, vol. 2, no. 4, p. 041006, Oct. 2015, doi: 10.1117/1.JMI.2.4.041006.

[3] H. Akbari *et al.*, "In vivo evaluation of EGFRvIII mutation in primary glioblastoma patients via complex multiparametric MRI signature," *Neuro-Oncol.*, vol. 20, no. 8, pp. 1068–1079, 05 2018, doi: 10.1093/neuonc/noy033.

[4] L. Song *et al.*, "Clinical, Conventional CT and Radiomic Feature-Based Machine Learning Models for Predicting ALK Rearrangement Status in Lung Adenocarcinoma Patients," *Front. Oncol.*, vol. 10, p. 369, 2020, doi: 10.3389/fonc.2020.00369.

[5] E. Rios Velazquez *et al.*, "Somatic Mutations Drive Distinct Imaging Phenotypes in Lung Cancer," *Cancer Res.*, vol. 77, no. 14, pp. 3922–3930, 15 2017, doi: 10.1158/0008-5472.CAN-17-0122.

[6] S. S. F. Yip *et al.*, "Associations Between Somatic Mutations and Metabolic Imaging Phenotypes in Non-Small Cell Lung Cancer," *J. Nucl. Med. Off. Publ. Soc. Nucl. Med.*, vol. 58, no. 4, pp. 569–576, 2017, doi: 10.2967/jnumed.116.181826.

[7] H. Saadani *et al.*, "Metabolic Biomarker-Based BRAFV600 Mutation Association and Prediction in Melanoma," *J. Nucl. Med. Off. Publ. Soc. Nucl. Med.*, vol. 60, no. 11, pp. 1545–1552, 2019, doi: 10.2967/jnumed.119.228312.

[8] J. H. Yoon *et al.*, "Radiomics in predicting mutation status for thyroid cancer: A preliminary study using radiomics features for predicting BRAFV600E mutations in papillary thyroid carcinoma," *PloS One*, vol. 15, no. 2, p. e0228968, 2020, doi: 10.1371/journal.pone.0228968.

[9] X. Liu *et al.*, "IDH mutation-specific radiomic signature in lower-grade gliomas," *Aging*, vol. 11, no. 2, pp. 673–696, 29 2019, doi: 10.18632/aging.101769.

[10] Y. Ren *et al.*, "Noninvasive Prediction of IDH1 Mutation and ATRX Expression Loss in Low-Grade Gliomas Using Multiparametric MR Radiomic Features," *J. Magn. Reson. Imaging JMRI*, vol. 49, no. 3, pp. 808–817, 2019, doi: 10.1002/jmri.26240.

[11] Y. Tan, W. Mu, X.-C. Wang, G.-Q. Yang, R. J. Gillies, and H. Zhang, "Whole-tumor radiomics analysis of DKI and DTI may improve the prediction of genotypes for astrocytomas: A preliminary study," *Eur. J. Radiol.*, vol. 124, p. 108785, Mar. 2020, doi: 10.1016/j.ejrad.2019.108785.

[12] Y. Han *et al.*, "Amide Proton Transfer Imaging in Predicting Isocitrate Dehydrogenase 1 Mutation Status of Grade II/III Gliomas Based on Support Vector Machine," *Front. Neurosci.*, vol. 14, p. 144, 2020, doi: 10.3389/fnins.2020.00144.

[13] Y. Li *et al.*, "Radiomic features predict Ki-67 expression level and survival in lower grade gliomas," *J. Neurooncol.*, vol. 135, no. 2, pp. 317–324, Nov. 2017, doi: 10.1007/s11060-017-2576-8.

[14] C. Su *et al.*, "Radiomics based on multicontrast MRI can precisely differentiate among glioma subtypes and predict tumour-proliferative behaviour," *Eur. Radiol.*, vol. 29, no. 4, pp. 1986–1996, Apr. 2019, doi: 10.1007/s00330-018-5704-8.

[15] P. Lohmann *et al.*, "Predicting IDH genotype in gliomas using FET PET radiomics," *Sci. Rep.*, vol. 8, no. 1, p. 13328, 06 2018, doi: 10.1038/s41598-018-31806-7.

[16] L. Li *et al.*, "A Non-invasive Radiomic Method Using 18F-FDG PET Predicts Isocitrate Dehydrogenase Genotype and Prognosis in Patients With Glioma," *Front. Oncol.*, vol. 9, p. 1183, 2019, doi: 10.3389/fonc.2019.01183.

[17] Z. Kong *et al.*, "Radiomics signature based on FDG-PET predicts proliferative activity in primary glioma," *Clin. Radiol.*, vol. 74, no. 10, p. 815.e15-815.e23, Oct. 2019, doi: 10.1016/j.crad.2019.06.019.

[18] M. Fan, H. Li, S. Wang, B. Zheng, J. Zhang, and L. Li, "Radiomic analysis reveals DCE-MRI features for prediction of molecular subtypes of breast cancer," *PloS One*, vol. 12, no. 2, p. e0171683, 2017, doi: 10.1371/journal.pone.0171683.

[19] W. Ma, Y. Ji, L. Qi, X. Guo, X. Jian, and P. Liu, "Breast cancer Ki67 expression prediction by DCE-MRI radiomics features," *Clin. Radiol.*, vol. 73, no. 10, p. 909.e1-909.e5, 2018, doi: 10.1016/j.crad.2018.05.027.

[20] M. Fan *et al.*, "Joint Prediction of Breast Cancer Histological Grade and Ki-67 Expression Level Based on DCE-MRI and DWI Radiomics," *IEEE J. Biomed. Health Inform.*, vol. 24, no. 6, pp. 1632–1642, Jun. 2020, doi: 10.1109/JBHI.2019.2956351.

[21] Y. Li *et al.*, "A CT-based radiomics nomogram for prediction of human epidermal growth factor receptor 2 status in patients with gastric cancer," *Chin. J. Cancer Res. Chung-Kuo Yen Cheng Yen Chiu*, vol. 32, no. 1, pp. 62–71, Feb. 2020, doi: 10.21147/j.issn.1000-9604.2020.01.08.

[22] Q.-W. Zhang *et al.*, "Personalized CT-based radiomics nomogram preoperative predicting Ki-67 expression in gastrointestinal stromal tumors: a multicenter development and validation cohort," *Clin. Transl. Med.*, vol. 9, no. 1, p. 12, Jan. 2020, doi: 10.1186/s40169-020-0263-4.

[23] Z. Ye *et al.*, "Texture analysis on gadoxetic acid enhanced-MRI for predicting Ki-67 status in hepatocellular carcinoma: A prospective study," *Chin. J. Cancer Res. Chung-Kuo Yen Cheng Yen Chiu*, vol. 31, no. 5, pp. 806–817, Oct. 2019, doi: 10.21147/j.issn.1000-9604.2019.05.10.

[24] B. Zhou, J. Xu, Y. Tian, S. Yuan, and X. Li, "Correlation between radiomic features based on contrast-enhanced computed tomography images and Ki-67 proliferation index in lung cancer: A preliminary study," *Thorac. Cancer*, vol. 9, no. 10, pp. 1235–1240, 2018, doi: 10.1111/1759-7714.12821.

[25] R.-Y. Chen *et al.*, "Associations of Tumor PD-1 Ligands, Immunohistochemical Studies, and Textural Features in 18F-FDG PET in Squamous Cell Carcinoma of the Head and Neck," *Sci. Rep.*, vol. 8, no. 1, p. 105, 08 2018, doi: 10.1038/s41598-017-18489-2.

[26] A. A. Ahmed *et al.*, "Radiomic mapping model for prediction of Ki-67 expression in adrenocortical carcinoma," *Clin. Radiol.*, vol. 75, no. 6, p. 479.e17-479.e22, Jun. 2020, doi: 10.1016/j.crad.2020.01.012.

[27] L. Yang *et al.*, "Can CT-based radiomics signature predict KRAS/NRAS/BRAF mutations in colorectal cancer?," *Eur. Radiol.*, vol. 28, no. 5, pp. 2058–2067, May 2018, doi: 10.1007/s00330-017-5146-8.

[28] C. H. Lim *et al.*, "Imaging phenotype using 18F-fluorodeoxyglucose positron emission tomography-based radiomics and genetic alterations of pancreatic ductal adenocarcinoma," *Eur. J. Nucl. Med. Mol. Imaging*, Jan. 2020, doi: 10.1007/s00259-020-04698-x.

[29] Y. Cui *et al.*, "Development and validation of a MRI-based radiomics signature for prediction of KRAS mutation in rectal cancer," *Eur. Radiol.*, vol. 30, no. 4, pp. 1948–1958, Apr. 2020, doi: 10.1007/s00330-019-06572-3.

[30] S. J. Hectors *et al.*, "MRI radiomics features predict immuno-oncological characteristics of hepatocellular carcinoma," *Eur. Radiol.*, vol. 30, no. 7, pp. 3759–3769, Jul. 2020, doi: 10.1007/s00330-020-06675-2.

[31] J. Yoon *et al.*, "Utility of CT radiomics for prediction of PD-L1 expression in advanced lung adenocarcinomas," *Thorac. Cancer*, vol. 11, no. 4, pp. 993–1004, 2020, doi: 10.1111/1759-7714.13352.

[32] Y. Li *et al.*, "MRI features predict p53 status in lower-grade gliomas via a machine-learning approach," *NeuroImage Clin.*, vol. 17, pp. 306–311, 2018, doi: 10.1016/j.nicl.2017.10.030.

[33] Z. Sun *et al.*, "Radiogenomic analysis of vascular endothelial growth factor in patients with diffuse gliomas," *Cancer Imaging Off. Publ. Int. Cancer Imaging Soc.*, vol. 19, no. 1, p. 68, Oct. 2019, doi: 10.1186/s40644-019-0256-y.

[34] P. Lin *et al.*, "MRI-based radiogenomics analysis for predicting genetic alterations in oncogenic signalling pathways in invasive breast carcinoma," *Clin. Radiol.*, vol. 75, no. 7, p. 561.e1-561.e11, Jul. 2020, doi: 10.1016/j.crad.2020.02.011.

[35] S.-W. Chen *et al.*, "Metabolic Imaging Phenotype Using Radiomics of [18F]FDG PET/CT Associated with Genetic Alterations of Colorectal Cancer," *Mol. Imaging Biol.*, vol. 21, no. 1, pp. 183–190, 2019, doi: 10.1007/s11307-018-1225-8.

[36] Y.-T. Peng *et al.*, "Preoperative Ultrasound Radiomics Signatures for Noninvasive Evaluation of Biological Characteristics of Intrahepatic Cholangiocarcinoma," *Acad. Radiol.*, vol. 27, no. 6, pp. 785–797, Jun. 2020, doi: 10.1016/j.acra.2019.07.029.

[37] Y. Zhu *et al.*, "Imaging-Genomic Study of Head and Neck Squamous Cell Carcinoma: Associations Between Radiomic Phenotypes and Genomic Mechanisms via Integration of The Cancer Genome Atlas and The Cancer Imaging Archive," *JCO Clin. Cancer Inform.*, vol. 3, pp. 1–9, 2019, doi: 10.1200/CCI.18.00073.
