# Supplementary material for: Systematic Review on the Association of Radiomics with Tumor Biological Endpoints
Source: Cancers (Basel). 2021 Jun 16;13(12):3015. doi: 10.3390/cancers13123015 (PMC8234501; doi:10.3390/cancers13123015)
Supplement: Supplementary file 1 [file cancers-13-03015-s001.zip › Supplementary_TableS10_BRAF.pdf]

Study	Tumor Site	Alteration	Modality	Dataset Origin	Training	Validation	Feature Reduction	Feature Robustnes s	# Radiomic Features	Additional features	Predictive power Measure = mean [95% confidence interval]	Open source
Yang et al.[1]	GI	v600E mutation	CT	National Cancer Center/Cancer Hospital, Chinese Academy of Medical Sciences and Peking Union Medical College, Beijing, China	61	57***	yes	yes	346	-	AUC = 0.829 [0.718–0.939]<br>Accuracy = 0.750 [0.623–0.845]	-
Saadani et al. [2]	Melanoma	v600E mutation	FDG-PET/CT	Netherlands Cancer Institute, Amsterdam, The Netherlands	70	10-CV	yes	no	480	SUVmax; SUVmean; SUVpeak; MTV; TLG; longest diameter	AUC = 0.62	-
Yoon et al. [3]	Thyroid	v600E mutation	US	Severance Hospital, Yonsei University College of Medicine, Seoul, South Korea	387	140***	yes	no	730	Age; tumor size; sex;	AUC = 0.629 [0.516-0.742]	-

Table S 10 An overview of the radiomic studies included for BRAF biomarker. * temporally independent internal validation;. Acronyms: v-raf murine sarcoma viral oncogene homolog B1 (BRAF), gastrointestinal (GI), computed tomography (CT), fluorodeoxyglucose positron emission tomography (FDG-PET), ultrasound (US), max, mean and peak standardized uptake value (SUVmax, SUVmean, SUVpeak), mean total lesion glycolysis (TLGmean), metabolic tumor volume (MTV), tumor, node and metastasis (TNM) , 10- fold cross-validation (10-CV), area under the curve (AUC).**

[1] L. Yang *et al.*, “Can CT-based radiomics signature predict KRAS/NRAS/BRAF mutations in colorectal cancer?,” *Eur. Radiol.*, vol. 28, no. 5, pp. 2058–2067, May 2018, doi: 10.1007/s00330-017-5146-8.

[2] H. Saadani *et al.*, “Metabolic Biomarker-Based BRAFV600 Mutation Association and Prediction in Melanoma,” *J. Nucl. Med. Off. Publ. Soc. Nucl. Med.*, vol. 60, no. 11, pp. 1545–1552, 2019, doi: 10.2967/jnumed.119.228312.

[3] J. H. Yoon *et al.*, “Radiomics in predicting mutation status for thyroid cancer: A preliminary study using radiomics features for predicting BRAFV600E mutations in papillary thyroid carcinoma,” *PloS One*, vol. 15, no. 2, p. e0228968, 2020, doi: 10.1371/journal.pone.0228968.
