# Supplementary material for: Systematic Review on the Association of Radiomics with Tumor Biological Endpoints
Source: Cancers (Basel). 2021 Jun 16;13(12):3015. doi: 10.3390/cancers13123015 (PMC8234501; doi:10.3390/cancers13123015)
Supplement: Supplementary file 1 [file cancers-13-03015-s001.zip › Supplementary_TableS11_PDL1.pdf]

Study	Tumor Site	Alteration	Modality	Dataset Origin	Training	Validation	Feature Reduction	Feature Robustness	# Radiomic Features	Additional features	Predictive power Measure = mean [95% confidence interval]	Open Source
Jiang et al. [1]	Lung	PD-L1 cutoff value of 1% and 50%	PET/CT	Shanghai Institute of Medical Imaging, Zhongshan Hospital of Fudan University, Shanghai, China	266	133*	yes	no	1,744	SUVmax; age; sex; smoking status; TNM stage; histology type	AUC = 0.97	-
Sun et al. [2]	Lung	High PD-L1 expression as ≥ 50%	CT	The First Affiliated Hospital of Soochow University, Suzhou City, China	260	130*	yes	yes	200	Age; sex; tumor location; CEA level; TNM stage; smoking status; histologic type; histologic grade	AUC = 0.848	-
Yoon et al. [3]	Lung	High PD-L1 expression as ≥50%	CT	Severance Hospital, Yonsei University College of Medicine, Seoul, South Korea	153	bootstrap	yes	yes	58	Age; sex; smoking history; stage; tumor size; tumor location; tumor type; tumor margin; internal characteristics of tumor; external characteristics of tumor; lung metastasis; pleural effusion; pleural nodularity; pericardial effusion; lymphadenopathy	c-index = 0.646	-
Hectors et al.[4]	Liver	expression	MRI, DWI	Icahn School of Medicine at Mount Sinai, New York, USA	48		no	no	196	Infiltrative pattern; presence of multiple lesions; extra-nodular growth; macrovascular invasion; tumor necrosis; tumor hemorrhage; tumor fat content; mosaic appearance; internal arteries; capsule; T2 hyper-intensity; ADC hypo-intensity; wash-in/wash-out; hepatobiliary phase hypo-intensity; ADCmin; ADCmean; ER in EA, LA, PV, LV and hepatobiliary phases; tumor size	Significant correlation ($p < 0.029$)	-
Chen et al. [5]	Head and Neck	PD-L1 cutoff value of 1% and 5%	FDG-PET	China Medical University, Taichung City, Taiwan	53	-	no	no	41	SUVmax, MTV, TLGmean; smoking history; tumor origin; TNM stage	AUC = 0.24	-

Table S 11 An overview of the radiomic studies included in the other cancers section. * internal validation; ** external validation; * temporally independent internal validation; ¹ negative correlation. Acronyms: programmed cell death ligand 1 (PD-L1), computed tomography (CT),**

fluorodeoxyglucose positron emission tomography (FDG-PET), minimum and mean values of apparent diffusion coefficient (ADCmin, ADCmean), maximal standardized uptake value (SUVmax), mean total lesion glycolysis (TLGmean), metabolic tumor volume (MTV), tumor, node and metastasis (TNM) , area under the curve (AUC), carcino-embryogenic antigen (CEA).

[1] M. Jiang *et al.*, "Assessing PD-L1 Expression Level by Radiomic Features From PET/CT in Nonsmall Cell Lung Cancer Patients: An Initial Result," *Acad. Radiol.*, vol. 27, no. 2, pp. 171–179, Feb. 2020, doi: 10.1016/j.acra.2019.04.016.

[2] Z. Sun *et al.*, "Radiomics study for predicting the expression of PD-L1 in non-small cell lung cancer based on CT images and clinicopathologic features," *J. X-Ray Sci. Technol.*, Mar. 2020, doi: 10.3233/XST-200642.

[3] J. Yoon *et al.*, "Utility of CT radiomics for prediction of PD-L1 expression in advanced lung adenocarcinomas," *Thorac. Cancer*, Feb. 2020, doi: 10.1111/1759-7714.13352.

[4] S. J. Hectors *et al.*, "MRI radiomics features predict immuno-oncological characteristics of hepatocellular carcinoma," *Eur. Radiol.*, Feb. 2020, doi: 10.1007/s00330-020-06675-2.

[5] R.-Y. Chen *et al.*, "Associations of Tumor PD-1 Ligands, Immunohistochemical Studies, and Textural Features in 18F-FDG PET in Squamous Cell Carcinoma of the Head and Neck," *Sci. Rep.*, vol. 8, no. 1, p. 105, 08 2018, doi: 10.1038/s41598-017-18489-2.
