# Supplementary material for: Systematic Review on the Association of Radiomics with Tumor Biological Endpoints
Source: Cancers (Basel). 2021 Jun 16;13(12):3015. doi: 10.3390/cancers13123015 (PMC8234501; doi:10.3390/cancers13123015)
Supplement: Supplementary file 1 [file cancers-13-03015-s001.zip › Supplementary_TableS12_TP53.pdf]

Study	Tumor Site	Alteration	Modality	Dataset Origin	Training	Validation	Feature Reduction	Feature Robustness	# Radiomic Features	Additional Features	Predictive power Measure = mean [95% confidence interval]	Open Source
Li et al. [1]	CNS	mutation	MRI	Chinese Glioma Genome Atlas, Beijing Tiantan Hospital, Beijing, China	180	92*	Yes	No	431	-	AUC = 0.763 Accuracy = 70.7%	Images and ROI
Zhang et al. [2]	CNS	mutation	MRI	TCGA/TCIA-LGG	73	30*	Yes	Yes	260	16 VASARI features	AUC = 0.869 Accuracy = 85.0%	Images, ROI
Lin et al. [3]	Breast	mutation	PWI	TCGA/TCIA-BRCA	88	LOOCV	yes	no	5,234	-	AUC = 0.886 [0.817 - 0.955]	images
Wang et al. [4]	Lung	mutation	CT	Nanjing Medical University Affiliated Cancer Hospital, Nanjing, China	41	20*	yes	no	718	78 clinical and pathological features (age, sex, smoking status, histological subtypes, pathological stages, etc.)	AUC = 0.656	code

Chen et al.[5]	GI	KRAS: Exon 2 codons 12 and 13 mutation TP-53: Exons 2-11 mutations	FDG-PET/CT	China Medical University Hospital, Taichung, Taiwan	74	-	yes	no	56	SUVmax, SUVpeak, SUVtot, MTV, TLGmax, TLGpeak, and TLGmean	AUC = 0.71 Accuracy = 62%	-
Lim et al. [6]	GI	mutation	FDG-PET/CT	Samsung Medical Center, Sungkyunkwan University School of Medicine, Gangnam-gu, Seoul, South Korea	48	-	no	yes	27	SUVmax, SUVmean, SUVstd, SUVkurt, SUVskew, SUVent, MTV, TLG	No correlation	Code (partially)
Zhu et al. [7]	Head and Neck	mutation	CT	TCIA/TCGA-HNSCC	126	5-CV	yes	yes	187	-	AUC = 0.641	Images, ROI

Table S 12 An overview of the radiomic studies included for TP-53 biomarker. * internal validation. Acronyms: tumor protein p53 (TP-53), central nervous system (CNS), gastrointestinal (GI), fluorodeoxyglucose positron emission tomography (FDG-PET), computed tomography (CT), perfusion weighted imaging (PWI), magnetic resonance imaging (MRI), metabolic tumor volume (MTV), max, mean, peak, standard deviation, skewness, kurtosis, entropy and total standardized uptake value (SUVmax, SUVmean, SUVpeak, SUVstd, SUVskew, SUVkurt, SUVent, SUVtot), max, peak and min of total lesion glycolysis (TLGmax, TLGmin, TLGpeak), area under the curve (AUC), leave-one-out- and 5-fold cross-validation (LOOCV, 5-CV), Visually AcceSAble Rembrandt Images (VASARI), The Cancer Imaging Archive / The Cancer Genome Atlas (TCIA/TCGA), Head and Neck Squamous Cell Carcinoma (HNSCC), BReast invasive CArcinoma (BRCA), Lower Grade Glioma (LGG), region of interest (ROI).

[1] Y. Li *et al.*, “MRI features predict p53 status in lower-grade gliomas via a machine-learning approach,” *NeuroImage Clin.*, vol. 17, pp. 306–311, 2018, doi: 10.1016/j.nicl.2017.10.030.

[2] X. Zhang *et al.*, “Radiomics Strategy for Molecular Subtype Stratification of Lower-Grade Glioma: Detecting IDH and TP53 Mutations Based on Multimodal MRI,” *J. Magn. Reson. Imaging JMRI*, vol. 48, no. 4, pp. 916–926, 2018, doi: 10.1002/jmri.25960.

[3] P. Lin *et al.*, “MRI-based radiogenomics analysis for predicting genetic alterations in oncogenic signalling pathways in invasive breast carcinoma,” *Clin. Radiol.*, vol. 75, no. 7, p. 561.e1-561.e11, Jul. 2020, doi: 10.1016/j.crad.2020.02.011.

[4] X. Wang *et al.*, "Decoding tumor mutation burden and driver mutations in early stage lung adenocarcinoma using CT-based radiomics signature," *Thorac. Cancer*, vol. 10, no. 10, pp. 1904–1912, Oct. 2019, doi: 10.1111/1759-7714.13163.

[5] S.-W. Chen *et al.*, "Metabolic Imaging Phenotype Using Radiomics of [18F]FDG PET/CT Associated with Genetic Alterations of Colorectal Cancer," *Mol. Imaging Biol.*, vol. 21, no. 1, pp. 183–190, 2019, doi: 10.1007/s11307-018-1225-8.

[6] C. H. Lim *et al.*, "Imaging phenotype using 18F-fluorodeoxyglucose positron emission tomography-based radiomics and genetic alterations of pancreatic ductal adenocarcinoma," *Eur. J. Nucl. Med. Mol. Imaging*, vol. 47, no. 9, pp. 2113–2122, Aug. 2020, doi: 10.1007/s00259-020-04698-x.

[7] Y. Zhu *et al.*, "Imaging-Genomic Study of Head and Neck Squamous Cell Carcinoma: Associations Between Radiomic Phenotypes and Genomic Mechanisms via Integration of The Cancer Genome Atlas and The Cancer Imaging Archive," *JCO Clin. Cancer Inform.*, vol. 3, pp. 1–9, 2019, doi: 10.1200/CCI.18.00073.
