# Supplementary material for: Systematic Review on the Association of Radiomics with Tumor Biological Endpoints
Source: Cancers (Basel). 2021 Jun 16;13(12):3015. doi: 10.3390/cancers13123015 (PMC8234501; doi:10.3390/cancers13123015)
Supplement: Supplementary file 1 [file cancers-13-03015-s001.zip › Supplementary_TableS13_KRAS.pdf]

Study	Tumor Site	Alteration	Modality	Dataset Origin	Training	Validation	Feature Reduction	Feature Robustness	# Radiomic Features	Additional features	Predictive power Measure = mean [95% confidence interval]	Open Source
Chen et al. [1]	Lung	mutation	MR	City of Hope Medical Center, Duarte, California, US	110	LOOCV	yes	yes	2,786	Age; sex; ethnicity; history of smoking; histology type; other metastatic sites	AUC = 0.985<br>Accuracy = 96.7%	-
Rios Velazquez et al. [2]	Lung	mutation	CT	Profile and Harvard-RT (Dana-Farber/Harvard Cancer Center IRB, Boston, MA), Tianjin (Tianjin Medical University IRB, Tianjin, China), Moffitt (IRB Moffitt Cancer Center, Tampa, FL)	353	352***	yes	yes	635	Age; sex; smoking status; ethnicity; clinical stage	AUC = 0.75 [0.69-080]<br>Accuracy = 66.0%	Images
Shiri et al. [3]	Lung	Exon 2 codons 12 and 13 mutations	PET/CT	TCIA	82	68*	yes	no	109	MTV, SUVmax, SUVpeak, SULmax, SULpeak	AUC = 0.83	Images, ROI, code
Yip et al. [4]	Lung	Exons 2–3 mutations	PET	Dana-Farber Cancer Institute, Brigham and Women's Hospital, and Harvard Medical School, Boston, Massachusetts	348	bootstrap	yes	no	68	MTV, SUV_{max}, SUV_{peak}, SUV_{mean}, and SUV_{tot}	KRAS: -	-
Yip et al. [5]	Lung	mutation	PET	Dana-Farber Cancer Institute, Brigham and Women's Hospital, and Harvard Medical School, Boston, Massachusetts	348	-	yes	yes	66	-	KRAS: -	-

Chen et al.[6]	GI	Exon 2 codons 12 and 13 mutation	FDG-PET/CT	China Medical University Hospital, Taichung, Taiwan	74	-	yes	no	56	SUVmax, SUVpeak, SUVtot, MTV, TLGmax, TLGpeak, and TLGmean	AUC = 0.79<br>Accuracy = 77%	-
Cui et al.[7]	GI	Exons 2-4 mutations	MRI	Shanxi Province Cancer Hospital, Taiyuan, China; Xinhua Hospital, Shanghai, China	213	91*<br>86**	yes	no	960	-	AUC* = 0.682 [0.569–0.794]<br>AUC** = 0.714 [0.602–0.827]	-
Lim et al. [8]	GI	mutation	FDG-PET/CT	Samsung Medical Center, Sungkyunkwan University School of Medicine, Gangnam-gu, Seoul, South Korea	48	-	no	yes	27	SUVmax, SUVmean, SUVstd, SUVkurt, SUVskew, SUVent, MTV, TLG	AUC = 0.829	Code (partially)
Meng et al. [9]	GI	exon 2 codons 12 and 13 mutation	MRI, DWI, PWI	Sixth Affiliated Hospital of Sun Yat-sen University. Guangzhou, China	197	148***	yes	yes	2,534	-	AUC = 0.651 [0.539 - 0.763]<br>Accuracy = 0.616	-
Oh et al.[10]	GI	A59T, G12A, G12C, G12D, G12F, G12R, G12S, G12V, G13D, G61H, and Q61 mutation	MRI	Research Institute and Hospital, National Cancer Center, Goyang, Korea	60	-	no	no	44	-	AUC = 0.884<br>Accuracy = 81.7%	-

Wu et al. [11]	GI	Exons 2-4 mutations	CT	South China University of Technology, Guangzhou, Guangdong Province, China	279	119***	yes	yes	2,634	2,208 DL features	c-index = 0.832 [0.762–0.905]	-
Yang et al.[12]	GI	Exons 2-4 mutations	CT	National Cancer Center/Cancer Hospital, Chinese Academy of Medical Sciences and Peking Union Medical College, Beijing, China	61	57***	yes	yes	346	-	AUC = 0.829 [0.718–0.939]<br>Accuracy = 0.750 [0.623–0.845]	-

Table S 13 An overview of the radiomic studies included for KRAS biomarker. * internal validation; ** external validation; * temporally independent internal validation. Acronyms: kirsten rat sarcoma viral oncogene homolog (KRAS), gastrointestinal (GI), computed tomography (CT), magnetic resonance imaging (MRI), positron emission tomography (PET), perfusion weighted imaging (PWI), diffusion weighted imaging (DWI), The Cancer Imaging Archive (TCIA), deep learning (DL), leave-one-out cross-validation (LOOCV), area under the curve (AUC), metabolic tumor volume (MTV), max, mean, peak, standard deviation, skewness, kurtosis, entropy and total standardized uptake value (SUVmax, SUVmean, SUVpeak, SUVstd, SUVskew, SUVkurt, SUVent, SUVtot), max, peak and min of total lesion glycolysis (TLGmax, TLGmin, TLGpeak), region of interest (ROI).**

[1] B. T. Chen *et al.*, "Radiomic prediction of mutation status based on MR imaging of lung cancer brain metastases," *Magn. Reson. Imaging*, vol. 69, pp. 49–56, Mar. 2020, doi: 10.1016/j.mri.2020.03.002.

[2] E. Rios Velazquez *et al.*, "Somatic Mutations Drive Distinct Imaging Phenotypes in Lung Cancer," *Cancer Res.*, vol. 77, no. 14, pp. 3922–3930, 15 2017, doi: 10.1158/0008-5472.CAN-17-0122.

[3] I. Shiri *et al.*, "Next-Generation Radiogenomics Sequencing for Prediction of EGFR and KRAS Mutation Status in NSCLC Patients Using Multimodal Imaging and Machine Learning Algorithms," *Mol. Imaging Biol.*, Mar. 2020, doi: 10.1007/s11307-020-01487-8.

[4] S. S. F. Yip *et al.*, "Associations Between Somatic Mutations and Metabolic Imaging Phenotypes in Non-Small Cell Lung Cancer," *J. Nucl. Med. Off. Publ. Soc. Nucl. Med.*, vol. 58, no. 4, pp. 569–576, 2017, doi: 10.2967/jnumed.116.181826.

[5] S. S. F. Yip, C. Parmar, J. Kim, E. Huynh, R. H. Mak, and H. J. W. L. Aerts, "Impact of experimental design on PET radiomics in predicting somatic mutation status," *Eur. J. Radiol.*, vol. 97, pp. 8–15, Dec. 2017, doi: 10.1016/j.ejrad.2017.10.009.

[6] S.-W. Chen *et al.*, "Metabolic Imaging Phenotype Using Radiomics of [18F]FDG PET/CT Associated with Genetic Alterations of Colorectal Cancer," *Mol. Imaging Biol.*, vol. 21, no. 1, pp. 183–190, 2019, doi: 10.1007/s11307-018-1225-8.

[7] Y. Cui *et al.*, "Development and validation of a MRI-based radiomics signature for prediction of KRAS mutation in rectal cancer," *Eur. Radiol.*, vol. 30, no. 4, pp. 1948–1958, Apr. 2020, doi: 10.1007/s00330-019-06572-3.

[8] C. H. Lim *et al.*, "Imaging phenotype using 18F-fluorodeoxyglucose positron emission tomography-based radiomics and genetic alterations of pancreatic ductal adenocarcinoma," *Eur. J. Nucl. Med. Mol. Imaging*, vol. 47, no. 9, pp. 2113–2122, Aug. 2020, doi: 10.1007/s00259-020-04698-x.

[9] X. Meng *et al.*, "Preoperative radiomic signature based on multiparametric magnetic resonance imaging for noninvasive evaluation of biological characteristics in rectal cancer," *Eur. Radiol.*, vol. 29, no. 6, pp. 3200–3209, Jun. 2019, doi: 10.1007/s00330-018-5763-x.

[10] J. E. Oh *et al.*, "Magnetic Resonance-Based Texture Analysis Differentiating KRAS Mutation Status in Rectal Cancer," *Cancer Res. Treat. Off. J. Korean Cancer Assoc.*, vol. 52, no. 1, pp. 51–59, Jan. 2020, doi: 10.4143/crt.2019.050.

[11] X. Wu *et al.*, "Deep Learning Features Improve the Performance of a Radiomics Signature for Predicting KRAS Status in Patients with Colorectal Cancer," *Acad. Radiol.*, Jan. 2020, doi: 10.1016/j.acra.2019.12.007.

[12] L. Yang *et al.*, "Can CT-based radiomics signature predict KRAS/NRAS/BRAF mutations in colorectal cancer?," *Eur. Radiol.*, vol. 28, no. 5, pp. 2058–2067, May 2018, doi: 10.1007/s00330-017-5146-8.
