# Supplementary material for: Systematic Review on the Association of Radiomics with Tumor Biological Endpoints
Source: Cancers (Basel). 2021 Jun 16;13(12):3015. doi: 10.3390/cancers13123015 (PMC8234501; doi:10.3390/cancers13123015)
Supplement: Supplementary file 1 [file cancers-13-03015-s001.zip › Supplementary_TableS14_KI67.pdf]

Study	Tumor Site	Alteration	Modality	Dataset Origin	Training	Validation	Feature Reduction	Feature Robustness	# Radiomic Features	Additional Features	Predictive power Measure = mean [95% confidence interval]	Open Source
Kong et al. [1]	CNS	High Ki-67 expression as > 10%	FDG-PET	Peking Union Medical College Hospital, Beijing, China	82	41*	Yes	No	1,561	Age; sex; metabolic pattern; SUVmax; SUVmean	AUC = 0.73 Accuracy = 78%	-
Li et al. [2]	CNS	High Ki-67 expression as > 25%	MRI	The Second Hospital of Hebei Medical University, Tangshan, Hebei, China	50	3-CV, 5-CV, bootstrap	Yes	No	396	-	AUC = 0.713 [0.568-0.832] Accuracy = 66.0%	-
Li et al. [3]	CNS	High Ki-67 expression as > 10%	MRI	Beijing Tiantan Hospital, Beijing, China; Chinese Glioma Genome Atlas	78	39*	Yes	No	431	-	AUC = 0.90 Accuracy = 88.6%	-
Su et al. [4]	CNS	High Ki-67 expression as > 25%	MRI, DWI, PWI	Tongji Hospital, Wuhan, Hubei, China	220	bootstrap	Yes	No	431	-	AUC = 0.936	-
Ugga et al. [5]	CNS	High Ki-67 expression as > 3%	MRI	University of Naples "Federico II" Neurosurgery, Naples, Italy	53	36*	Yes	Yes	1,128	-	AUC = 0.87 Accuracy = 91.67%	-
Antunovic et al. [6]	Breast	Ki-67: High expression at >20%	FDG-PET/CT	Humanitas Hospital, Milan, Italy	43	-	yes	no	20	MTV, SUVmean and TLG	No significant correlation	-
Fan et al. [7]	Breast	mutation	PWI, DWI	First Affiliated Hospital of Zhejiang Chinese Medical University, Hangzhou, China	144	LOOCV	yes	no	97	-	AUC = 0.811	Code

Liang et al.[8]	Breast	High expression at >14%	MRI	Guangdong General Hospital, Guangdong Academy of Medical Sciences, Guangzhou, China & Southern Medical University, Guangzhou, Guangdong, China	200	118***	yes	yes	10,207	-	AUC = 0.740 [0.645,0.836] Accuracy = 0.729	-
Ma et al. [9]	Breast	High expression at >14%	PWI	Tianjin Medical University Cancer Institute and Hospital, National Clinical Research Center for Cancer, Tianjin, China	159	10-CV	yes	no	56	-	AUC = 0.773 Accuracy = 0.757	-
Monti et al.[10]	Breast	mutation	PWI	Hospital of Moscati, Avellino, Italy; Institute for Hospitalization and Healthcare SDN, Naples, Italy	HER-2: 48 Ki-67: 49	bootstrap	yes	no	163	Pharmacokinetic maps	AUC = 0.811 Accuracy = 0.677	
Tagliafico et al.[11]	Breast	High expression at >14%	DBT	Emergency Radiology, IRCCS Policlinico San Martino, Genova, Italy	70	bootstrap	yes	no	106	-	AUC = 0.698	Code, features
Zhang et al.[12]	Breast	High expression at >14%	DWI	The Second Hospital, Dalian Medical University, Dalian, China	101	27*	yes	no	1,029	-	AUC = 0.72 [0.495 - 0.857] Accuracy = 0.70	-
Zhou et al.[13]	Breast	High expression at >20%	PWI	The Affiliated Huaian No. 1 People's Hospital of	126	5-CV	yes	yes	386	-	AUC = 0.74 Accuracy = 0.69	

				Nanjing Medical University, China								
Gu et al. [14]	Lung	High Ki-67 expression as >50%	CT	The Third Xiangya Hospital of Central South University, Hunan, China	245	10-CV	yes	no	103	Lobulation sign; spicule sign; cavitation; cystic necrosis; pleural indentation; pleural effusion	AUC = 0.782	-
Zhou et al. [15]	Lung	High Ki-67 expression as > 40%	CT	Tianjin Medical University Cancer Institute and Hospital, Tianjin, China	110	-	yes	no	105	Age; sex; smoking history; histological subtype; TNM stage	AUC = 0.77	code
Liang et al.[16]	GI	mutation	CT	The First Affiliated Hospital, Hangzhou, Zhejiang, China; Second Affiliated Hospital, Hangzhou, Zhejiang, China	86	51**	yes	no	467	Clinical stage	Significant correlation ($p < 0.0001$)	-
Meng et al. [17]	GI	High expression at >40% HER-2:	MRI, DWI, PWI	Sixth Affiliated Hospital of Sun Yat-sen University. Guangzhou, China	197	148***	yes	yes	2,534	-	AUC = 0.699 [0.611 - 0.788] Accuracy = 0.582	-
Zhang et al.[18]	GI	High expression as ≥ 10%	CT	Renji Hospital, Huangpu, Shanghai, China; Zhongshan Hospital, Shanghai, China; Sir Run Shaw Hospital, Hangzhou, Zhejiang, China and First Affiliated Hospital of Wenzhou Medical University, Wenzhou, China	148	41* 150**	yes	yes	833	Tumor size	AUC* = 0.828 [0.681–0.974] AUC** = 0.784 [0.701–0.868] Accuracy* = 68.29% Accuracy** = 73.33%	Images/data on request

Peng et al. [19]	Liver	High expression at ≥ 10%	US	First Affiliated Hospital of Guangxi Medical University, Nanning, Guangxi, China	63	27*	yes	no	1,076	-	AUC = 0.848 Accuracy = 0.889	-
Yao et al. [116]	Liver	High expression at ≥ 25%	US	Zhongshan Hospital, Fudan University, Shanghai, China	47	LOOCV	yes	no	-	2560 dictionary-based image features	AUC = 0.94 [0.87 - 0.97] Accuracy = 93%	Images on request
Ye et al. [20]	Liver	High expression at ≥ 15%	MRI	West China Hospital, Sichuan, China	89	10-CV	yes	no	396	Serum level of alpha-fetoprotein; hepatitis B surface antigen; hepatitis C antibody; Barcelona-Clinic Liver Cancer classification; cirrhosis; multifocality; arterial phase hyper-enhancement; washout, capsule integrity, internal arteries, tumor margin, enhancing capsule, hepato-biliary phase hypo-intensity	c-index: 0.936 [0.863−0.977]	-
Ahmed et al. [21]	Adrenal gland	High expression at ≥ 10%	CT	MD Anderson Cancer Center, Texas, US	53	-	no	no	106	-	AUC = 0.78	-
Chen et al. [22]	Head and Neck	expression	FDG-PET	China Medical University, Taichung City, Taiwan	53	-	no	no	41	SUVmax, MTV, TLGmean; smoking	Correlation ($p < 0.05$)	-

history; tumor origin; TNM stage

Table S 14 An overview of the radiomic studies included in the liver cancer section. * internal validation; ** external validation; * temporally independent internal validation. Acronyms: antigen Ki-67 (Ki-67), central nervous system (CNS), gastrointestinal (GI), computed tomography (CT), magnetic resonance imaging (MRI), fluorodeoxyglucose positron emission tomography (FDG-PET), ultrasound (US), diffusion weighted imaging (DWI), perfusion weighted imaging (PWI), area under the curve (AUC), leave-one-out-, 3-, 5- and 10- fold cross-validation (LOOCV, 3-, 5-,10-CV), max, mean and peak standardized uptake value (SUVmax, SUVmean, SUVpeak), mean total lesion glycolysis (TLGmean), metabolic tumor volume (MTV), tumor, node and metastasis (TNM).**

[1] Z. Kong *et al.*, “Radiomics signature based on FDG-PET predicts proliferative activity in primary glioma,” *Clin. Radiol.*, vol. 74, no. 10, p. 815.e15-815.e23, Oct. 2019, doi: 10.1016/j.crad.2019.06.019.

[2] J. Li, S. Liu, Y. Qin, Y. Zhang, N. Wang, and H. Liu, “High-order radiomics features based on T2 FLAIR MRI predict multiple glioma immunohistochemical features: A more precise and personalized gliomas management,” *PloS One*, vol. 15, no. 1, p. e0227703, 2020, doi: 10.1371/journal.pone.0227703.

[3] Y. Li *et al.*, “Radiomic features predict Ki-67 expression level and survival in lower grade gliomas,” *J. Neurooncol.*, vol. 135, no. 2, pp. 317–324, Nov. 2017, doi: 10.1007/s11060-017-2576-8.

[4] C. Su *et al.*, “Radiomics based on multicontrast MRI can precisely differentiate among glioma subtypes and predict tumour-proliferative behaviour,” *Eur. Radiol.*, vol. 29, no. 4, pp. 1986–1996, Apr. 2019, doi: 10.1007/s00330-018-5704-8.

[5] L. Ugga *et al.*, “Prediction of high proliferative index in pituitary macroadenomas using MRI-based radiomics and machine learning,” *Neuroradiology*, vol. 61, no. 12, pp. 1365–1373, Dec. 2019, doi: 10.1007/s00234-019-02266-1.

[6] L. Antunovic *et al.*, “[18F]FDG PET/CT features for the molecular characterization of primary breast tumors,” *Eur. J. Nucl. Med. Mol. Imaging*, vol. 44, no. 12, pp. 1945–1954, Nov. 2017, doi: 10.1007/s00259-017-3770-9.

[7] M. Fan *et al.*, “Joint Prediction of Breast Cancer Histological Grade and Ki-67 Expression Level Based on DCE-MRI and DWI Radiomics,” *IEEE J. Biomed. Health Inform.*, vol. 24, no. 6, pp. 1632–1642, Jun. 2020, doi: 10.1109/JBHI.2019.2956351.

[8] C. Liang *et al.*, “An MRI-based Radiomics Classifier for Preoperative Prediction of Ki-67 Status in Breast Cancer,” *Acad. Radiol.*, vol. 25, no. 9, pp. 1111–1117, 2018, doi: 10.1016/j.acra.2018.01.006.

[9] W. Ma, Y. Ji, L. Qi, X. Guo, X. Jian, and P. Liu, “Breast cancer Ki67 expression prediction by DCE-MRI radiomics features,” *Clin. Radiol.*, vol. 73, no. 10, p. 909.e1-909.e5, 2018, doi: 10.1016/j.crad.2018.05.027.

[10] S. Monti *et al.*, “DCE-MRI Pharmacokinetic-Based Phenotyping of Invasive Ductal Carcinoma: A Radiomic Study for Prediction of Histological Outcomes,” *Contrast Media Mol. Imaging*, vol. 2018, p. 5076269, 2018, doi: 10.1155/2018/5076269.

[11] A. S. Tagliafico *et al.*, “Breast cancer Ki-67 expression prediction by digital breast tomosynthesis radiomics features,” *Eur. Radiol. Exp.*, vol. 3, no. 1, p. 36, 14 2019, doi: 10.1186/s41747-019-0117-2.

[12] Y. Zhang *et al.*, "Invasive ductal breast cancer: preoperative predict Ki-67 index based on radiomics of ADC maps," *Radiol. Med. (Torino)*, vol. 125, no. 2, pp. 109–116, Feb. 2020, doi: 10.1007/s11547-019-01100-1.

[13] X. Zhou *et al.*, "Radiomic features of Pk-DCE MRI parameters based on the extensive Tofts model in application of breast cancer," *Phys. Eng. Sci. Med.*, vol. 43, no. 2, pp. 517–524, Jun. 2020, doi: 10.1007/s13246-020-00852-9.

[14] Q. Gu *et al.*, "Machine learning-based radiomics strategy for prediction of cell proliferation in non-small cell lung cancer," *Eur. J. Radiol.*, vol. 118, pp. 32–37, Sep. 2019, doi: 10.1016/j.ejrad.2019.06.025.

[15] B. Zhou, J. Xu, Y. Tian, S. Yuan, and X. Li, "Correlation between radiomic features based on contrast-enhanced computed tomography images and Ki-67 proliferation index in lung cancer: A preliminary study," *Thorac. Cancer*, vol. 9, no. 10, pp. 1235–1240, 2018, doi: 10.1111/1759-7714.12821.

[16] W. Liang *et al.*, "A Combined Nomogram Model to Preoperatively Predict Histologic Grade in Pancreatic Neuroendocrine Tumors," *Clin. Cancer Res. Off. J. Am. Assoc. Cancer Res.*, vol. 25, no. 2, pp. 584–594, 15 2019, doi: 10.1158/1078-0432.CCR-18-1305.

[17] X. Meng *et al.*, "Preoperative radiomic signature based on multiparametric magnetic resonance imaging for noninvasive evaluation of biological characteristics in rectal cancer," *Eur. Radiol.*, vol. 29, no. 6, pp. 3200–3209, Jun. 2019, doi: 10.1007/s00330-018-5763-x.

[18] Q.-W. Zhang *et al.*, "Personalized CT-based radiomics nomogram preoperative predicting Ki-67 expression in gastrointestinal stromal tumors: a multicenter development and validation cohort," *Clin. Transl. Med.*, vol. 9, no. 1, p. 12, Jan. 2020, doi: 10.1186/s40169-020-0263-4.

[19] Y.-T. Peng *et al.*, "Preoperative Ultrasound Radiomics Signatures for Noninvasive Evaluation of Biological Characteristics of Intrahepatic Cholangiocarcinoma," *Acad. Radiol.*, Sep. 2019, doi: 10.1016/j.acra.2019.07.029.

[20] Z. Ye *et al.*, "Texture analysis on gadoxetic acid enhanced-MRI for predicting Ki-67 status in hepatocellular carcinoma: A prospective study," *Chin. J. Cancer Res. Chung-Kuo Yen Cheng Yen Chiu*, vol. 31, no. 5, pp. 806–817, Oct. 2019, doi: 10.21147/j.issn.1000-9604.2019.05.10.

[21] A. A. Ahmed *et al.*, "Radiomic mapping model for prediction of Ki-67 expression in adrenocortical carcinoma," *Clin. Radiol.*, vol. 75, no. 6, p. 479.e17-479.e22, Jun. 2020, doi: 10.1016/j.crad.2020.01.012.

[22] R.-Y. Chen *et al.*, "Associations of Tumor PD-1 Ligands, Immunohistochemical Studies, and Textural Features in 18F-FDG PET in Squamous Cell Carcinoma of the Head and Neck," *Sci. Rep.*, vol. 8, no. 1, p. 105, 08 2018, doi: 10.1038/s41598-017-18489-2.
