# Supplementary material for: Systematic Review on the Association of Radiomics with Tumor Biological Endpoints
Source: Cancers (Basel). 2021 Jun 16;13(12):3015. doi: 10.3390/cancers13123015 (PMC8234501; doi:10.3390/cancers13123015)
Supplement: Supplementary file 1 [file cancers-13-03015-s001.zip › Supplementary_TableS5_IDH.pdf]

Study	Tumor Site	Alteration	Modality	Dataset Origin	Training	Validation	Feature Reduction	Feature Robustness	# Radiomic Features	Additional Features	Predictive power Measure = mean [95% confidence interval]	Open Source
Arita et al.[1]	CNS	Isoforms 1 (codon 132) and 2 (codon 172) mutations	MRI	Osaka International Cancer Institute, Osaka, Japan; National Cancer Center Research Institute, Tokyo, Japan	111	58*	Yes	No	50	59 tumor spatial location features	Accuracy = 87%	Code Features
Choi et al. [2]	CNS	Isoforms 1 (codon 132) mutation	MRI	TCIA/TCGA-GBM; St. Mary's Hospital, Seoul, South Korea	45	91**	Yes	No	107	-	AUC = 0.904 [0.805, 1.0] Accuracy = 86.8 % [63.7, 97.8]	Images and ROI partially
Fukuma et al. [3]	CNS	Isoforms 1 (codon 132) and 2 (codon 172) mutations	MRI	Osaka International Cancer Institute, Osaka, Japan; National Cancer Center Research Institute, Tokyo, Japan	127	10-CV	Yes	No	61	3 tumor spatial location features; 4000 DL features; age	Accuracy = 73.1 %	-
Han et al. [4]	CNS	Isoforms 1 (codon 132) mutation	APTw	Tangdu Hospital, Xian, China	49	10*	Yes	Yes	1,044	-	AUC = 0.952 Accuracy = 0.892	Images on request
Kim et al. [5]	CNS	Isoforms 1 (codon 132) mutation	MRI, DWI, PWI	Asan Medical Center, Seoul, South Korea	127	28***	Yes	Yes	6,472	-	AUC = 0.747 [0.66–0.83] Accuracy = 65.3%	-
Kuthuru et al. [6]	CNS	Isoforms 1 (codon 132) mutation	MRI	TCGA/TCIA-LGG	108	10-CV	No	No	No	> 35,000 histogram of oriented gradients, scale-	AUC = 0.8224 [0.7856-0.8575]	Images and ROI

									invariant feature transform and voxel intensities		
Lee et al. [7]	CNS	Isoforms 1 (codon 132) mutation	MRI, DWI, PWI	Samsung Medical Center, Seoul, South Korea	88	35***	Yes	No	82	-	Accuracy = 83.4%	-
Li et al. [8]	CNS	Isoforms 1 (codon 132) and 2 (codon 172) mutations	FDG-PET	Peking Union Medical College Hospital, Beijing, China	84	43*	Yes	No	1,561	Age; sex; metabolic pattern; SUVmax; SUVmean	AUC = 0.900 [0.877–0.923]	Code
Li et al. [9]	CNS	Isoforms 1 (codon 132) and 2 (codon 172) mutations	MRI	TCGA/TCIA-GBM; Sun Yat-sen University Cancer Center, Guangzhou, China; The 3rd Affiliated Hospital of Sun Yat-sen University, Guangzhou, China; Guangzhou General Hospital of Guangzhou Military Command, Guangzhou, China	118	107**	Yes	No	1,614	Sex; age; KPS	AUC = 0.96 Accuracy = 97%	Images and ROI (partially)
Li et al. [10]	CNS	Isoforms 1 (codon 132) mutation	MRI	Huashan Hospital, Shangai, China	229	LOOCV	Yes	No	671	16,384 DLR features	AUC = 0.9521 Accuracy = 92.44%	-
Liu et al. [11]	CNS	Isoforms 1 (codon 132) mutation	MRI	Beijing Tiantan Hospital, Beijing, China;	158	102***	Yes	Yes	431	-	AUC = 0.99	-
Lohmann et al. [12]	CNS	Isoforms 1 (codon 132) mutation	FET-PET	University Hospital RWTH Aachen	84	5-CV, 10-CV	No	Yes	33	Slope; TTP; mean tumor-to-brain ratio; maximum	AUC = 0.79 Accuracy = 80.0%	-

										tumor-to-brain ratio		
Lu et al. [13]	CNS	mutation	MRI, DWI	TCGA/TCIA-LGG; TCGA/TCIA-GBM; TCIA-REMBRANDT; Taipei Medical University, Taipei, Taiwan	214	70**	Yes	No	39,212	-	Accuracy = 88.9 – 91.7%	Images and ROI (partially)
Park et al. [14]	CNS	Isoforms 1 (codon 132) mutation	MRI, DWI	Yonsei University, Seoul, South Korea	168	10-CV	Yes	No	411	-	AUC = 0.900 [0.855–0.945]	-
Ren et al. [15]	CNS	Isoforms 1 (codon 132) mutation	MRI, DWI, PWI	Huashan Hospital, Shangai, China	57	10-CV	Yes	No	260	10 VASARI features; age; sex; Ki-67	AUC = 0.931 Accuracy = 94.74%	-
Tan et al. [16]	CNS	mutation	DKI, DWI	Shanxi Medical University Shanxi, China	62	bootstrap	Yes	No	728	Age; sex; grade; tumor size; tumor border; hemorrhage; cystic and necrosis; edema degree; enhancement style; enhancement degree; signal characteristics; 6 tumor location features; mean diffusivity value; mean kurtosis value	AUC = 0.885 [0.802-0.955] Accuracy = 80.6% [71.0-90.3]	-

Tan et al. [17]	CNS	Isoforms 1 (codon 132) and 2 (codon 172) mutations	MRI, DWI	Shanxi Medical University Shanxi, China	74	31*	Yes	Yes	3,882	Age; sex; grade; tumor size; tumor border; hemorrhage; cystic and necrosis; edema degree; enhancement style; enhancement degree; signal characteristics ; 6 tumor location features	AUC = 0.900 [0.859–0.941] Accuracy = 87.1%	Features, ROI
Tontong Liu et al. [18]	CNS	Isoforms 1 (codon 132) mutation	MRI	Huashan Hospital, Shangai, China	110	LOOCV	Yes	No	671	-	AUC = 0.90 Accuracy = 0.85	-
Wu et al. [19]	CNS	Isoforms 1 (codon 132) mutation	MRI	TCGA/TCIA-LGG; TCGA/TCIA-GBM	126	bootstrap	Yes	No	698	6 tumor growth model parameters	AUC = 0.931 Accuracy = 0.885	Images, ROI
Wu et al. [20]	CNS	Isoforms 1 (codon 132) mutation	MRI	Huashan Hospital, Shangai, China	80	25*	Yes	No	-	968 dictionary features	Accuracy = 88.0%	-
Yu et al. [21]	CNS	Isoforms 1 (codon 132) mutation	MRI	Huashan Hospital, Shangai, China	92	LOOCV	No	No	-	116 tumor spatial location features	AUC = 0.71 Accuracy = 72.0%	-
Yu et al. [22]	CNS	Isoforms 1 (codon 132) mutation	MRI	Huashan Hospital, Shangai, China	110	LOOCV	Yes	No	671	-	AUC = 0.86 Accuracy = 80.0 %	-

Zhang et al. [23]	CNS	mutation	MRI	TCGA/TCIA-LGG	73	30*	Yes	Yes	260	16 VASARI features	IDH: AUC = 0.792 Accuracy = 80.0%	Images, ROI
Zhou et al. [24]	CNS	mutation	MRI	TCGA/TCIA-LGG	84	bootstrap	Yes	No	3,360	30 VASARI features; age; sex; KPS; histological type; grade; laterality; location	AUC = 0.86	Images, ROI, code

Table S 5 An overview of the radiomic studies included for IDH biomarker. * internal validation; ** external validation; * temporally independent internal validation. Acronyms: isocitrate dehydrogenase (IDH), central nervous system (CNS), magnetic resonance imaging (MRI), diffusion weighted imaging (DWI), amide proton transfer-weighted imaging (APTw), fluorodeoxyglucose positron emission tomography (FDG-PET), fluoroethyl tyrosine positron emission tomography (FET-PET), perfusion weighted imaging (PWI), max and mean standardized uptake value (SUVmax, SUVmean), The Cancer Imaging Archive / The Cancer Genome Atlas (TCIA/TCGA), glioblastoma (GBM), lower-grade glioma (LGG), Karnofsky Performance Status (KPS), The Repository of Molecular Brain Neoplasia Data (REMBRANDT), Visually AccesSAble Rembrandt Images (VASARI), deep learning (DL), deep learning radiomics (DLR), time-to-peak (TTP), leave-one-out cross-validation (LOOCV), 3-, 5- and 10-fold cross-validation (3-, 5- and 10-CV), area under the curve (AUC).**

[1] H. Arita *et al.*, "Lesion location implemented magnetic resonance imaging radiomics for predicting IDH and TERT promoter mutations in grade II/III gliomas," *Sci. Rep.*, vol. 8, no. 1, p. 11773, 06 2018, doi: 10.1038/s41598-018-30273-4.

[2] Y. Choi *et al.*, "IDH1 mutation prediction using MR-based radiomics in glioblastoma: comparison between manual and fully automated deep learning-based approach of tumor segmentation," *Eur. J. Radiol.*, vol. 128, p. 109031, Jul. 2020, doi: 10.1016/j.ejrad.2020.109031.

[3] R. Fukuma *et al.*, "Prediction of IDH and TERT promoter mutations in low-grade glioma from magnetic resonance images using a convolutional neural network," *Sci. Rep.*, vol. 9, no. 1, p. 20311, 30 2019, doi: 10.1038/s41598-019-56767-3.

[4] Y. Han *et al.*, "Amide Proton Transfer Imaging in Predicting Isocitrate Dehydrogenase 1 Mutation Status of Grade II/III Gliomas Based on Support Vector Machine," *Front. Neurosci.*, vol. 14, p. 144, 2020, doi: 10.3389/fnins.2020.00144.

[5] M. Kim *et al.*, "Diffusion- and perfusion-weighted MRI radiomics model may predict isocitrate dehydrogenase (IDH) mutation and tumor aggressiveness in diffuse lower grade glioma," *Eur. Radiol.*, vol. 30, no. 4, pp. 2142–2151, Apr. 2020, doi: 10.1007/s00330-019-06548-3.

[6] S. Kuthuru *et al.*, “A Visually Interpretable, Dictionary-Based Approach to Imaging-Genomic Modeling, With Low-Grade Glioma as a Case Study,” *Cancer Inform.*, vol. 17, p. 1176935118802796, 2018, doi: 10.1177/1176935118802796.
[7] M. H. Lee *et al.*, “Prediction of IDH1 Mutation Status in Glioblastoma Using Machine Learning Technique Based on Quantitative Radiomic Data,” *World Neurosurg.*, vol. 125, pp. e688–e696, 2019, doi: 10.1016/j.wneu.2019.01.157.
[8] L. Li *et al.*, “A Non-invasive Radiomic Method Using 18F-FDG PET Predicts Isocitrate Dehydrogenase Genotype and Prognosis in Patients With Glioma,” *Front. Oncol.*, vol. 9, p. 1183, 2019, doi: 10.3389/fonc.2019.01183.
[9] Z.-C. Li *et al.*, “Multiregional radiomics profiling from multiparametric MRI: Identifying an imaging predictor of IDH1 mutation status in glioblastoma,” *Cancer Med.*, vol. 7, no. 12, pp. 5999–6009, 2018, doi: 10.1002/cam4.1863.
[10] Z. Li, Y. Wang, J. Yu, Y. Guo, and W. Cao, “Deep Learning based Radiomics (DLR) and its usage in noninvasive IDH1 prediction for low grade glioma,” *Sci. Rep.*, vol. 7, no. 1, p. 5467, 14 2017, doi: 10.1038/s41598-017-05848-2.
[11] X. Liu *et al.*, “IDH mutation-specific radiomic signature in lower-grade gliomas,” *Aging*, vol. 11, no. 2, pp. 673–696, 29 2019, doi: 10.18632/aging.101769.
[12] P. Lohmann *et al.*, “Predicting IDH genotype in gliomas using FET PET radiomics,” *Sci. Rep.*, vol. 8, no. 1, p. 13328, 06 2018, doi: 10.1038/s41598-018-31806-7.
[13] C.-F. Lu *et al.*, “Machine Learning-Based Radiomics for Molecular Subtyping of Gliomas,” *Clin. Cancer Res. Off. J. Am. Assoc. Cancer Res.*, vol. 24, no. 18, pp. 4429–4436, 15 2018, doi: 10.1158/1078-0432.CCR-17-3445.
[14] C. J. Park *et al.*, “Diffusion tensor imaging radiomics in lower-grade glioma: improving subtyping of isocitrate dehydrogenase mutation status,” *Neuroradiology*, vol. 62, no. 3, pp. 319–326, Mar. 2020, doi: 10.1007/s00234-019-02312-y.
[15] Y. Ren *et al.*, “Noninvasive Prediction of IDH1 Mutation and ATRX Expression Loss in Low-Grade Gliomas Using Multiparametric MR Radiomic Features,” *J. Magn. Reson. Imaging JMRI*, vol. 49, no. 3, pp. 808–817, 2019, doi: 10.1002/jmri.26240.
[16] Y. Tan, W. Mu, X.-C. Wang, G.-Q. Yang, R. J. Gillies, and H. Zhang, “Whole-tumor radiomics analysis of DKI and DTI may improve the prediction of genotypes for astrocytomas: A preliminary study,” *Eur. J. Radiol.*, vol. 124, p. 108785, Mar. 2020, doi: 10.1016/j.ejrad.2019.108785.
[17] Y. Tan *et al.*, “A radiomics nomogram may improve the prediction of IDH genotype for astrocytoma before surgery,” *Eur. Radiol.*, vol. 29, no. 7, pp. 3325–3337, Jul. 2019, doi: 10.1007/s00330-019-06056-4.
[18] null Tongtong Liu *et al.*, “A mRMRMSRC feature selection method for radiomics approach,” *Conf. Proc. Annu. Int. Conf. IEEE Eng. Med. Biol. Soc. IEEE Eng. Med. Biol. Soc. Annu. Conf.*, vol. 2017, pp. 616–619, 2017, doi: 10.1109/EMBC.2017.8036900.
[19] S. Wu, J. Meng, Q. Yu, P. Li, and S. Fu, “Radiomics-based machine learning methods for isocitrate dehydrogenase genotype prediction of diffuse gliomas,” *J. Cancer Res. Clin. Oncol.*, vol. 145, no. 3, pp. 543–550, Mar. 2019, doi: 10.1007/s00432-018-2787-1.
[20] G. Wu *et al.*, “Sparse Representation-Based Radiomics for the Diagnosis of Brain Tumors,” *IEEE Trans. Med. Imaging*, vol. 37, no. 4, pp. 893–905, 2018, doi: 10.1109/TMI.2017.2776967.
[21] J. Yu *et al.*, “Anatomical location differences between mutated and wild-type isocitrate dehydrogenase 1 in low-grade gliomas,” *Int. J. Neurosci.*, vol. 127, no. 10, pp. 873–880, Oct. 2017, doi: 10.1080/00207454.2016.1270278.
[22] J. Yu *et al.*, “Noninvasive IDH1 mutation estimation based on a quantitative radiomics approach for grade II glioma,” *Eur. Radiol.*, vol. 27, no. 8, pp. 3509–3522, Aug. 2017, doi: 10.1007/s00330-016-4653-3.

[23] X. Zhang *et al.*, "Radiomics Strategy for Molecular Subtype Stratification of Lower-Grade Glioma: Detecting IDH and TP53 Mutations Based on Multimodal MRI," *J. Magn. Reson. Imaging JMRI*, vol. 48, no. 4, pp. 916–926, 2018, doi: 10.1002/jmri.25960.

[24] H. Zhou *et al.*, "MRI features predict survival and molecular markers in diffuse lower-grade gliomas," *Neuro-Oncol.*, vol. 19, no. 6, pp. 862–870, 01 2017, doi: 10.1093/neuonc/now256.
