# Supplementary material for: Systematic Review on the Association of Radiomics with Tumor Biological Endpoints
Source: Cancers (Basel). 2021 Jun 16;13(12):3015. doi: 10.3390/cancers13123015 (PMC8234501; doi:10.3390/cancers13123015)
Supplement: Supplementary file 1 [file cancers-13-03015-s001.zip › Supplementary_TableS6_EGFR.pdf]

Study	Tumor Site	Alteration	Modality	Dataset Origin	Training	Validation	Feature Reduction	Feature Robustness	# Radiomic Features	Additional Features	Predictive power Measure = mean [95% confidence interval]	Open Source
Akbari et al.[1]	CNS	Variant III mutation (deletion of exons 2-7)	MRI, DWI, PWI	Hospital of the University of Pennsylvania, Philadelphia, US	75	54*	No	No	421	16 tumor spatial location features; peritumoral heterogeneity index	AUC = 0.92 Accuracy = 88.9%	Code
Binder et al. [2]	CNS	Extracellular A289D/T/V, R108G/K and G598V mutations	MRI, PWI, DWI	Hospital of the University of Pennsylvania, Philadelphia, US	260	-	Yes	No	2,088	11 tumor spatial location features; 5 glioma diffusion properties from tumor biophysical models	Significant correlation ($p < 0.0444$)	Code
Lee et al. [3]	CNS	mutation	MRI	TCGA/TCIA-GBM	44	3-CV	No	No	-	36 spatial diversity features	AUC = 0.845 Accuracy = 0.79	Images and ROI
Li et al. [4]	CNS	High EGFR expression as > 30%	MRI	Beijing Tiantan Hospital, Beijing, China	200	70*	Yes	No	431	-	AUC = 0.95 Accuracy = 90.0%	
Rathore et al. [5]	CNS	Variant III mutation (deletion of exons 2-7)	MRI, DWI, PWI	Hospital of the University of Pennsylvania, Philadelphia, US	107	10-CV	Yes	No	255	9 tumor spatial location features; 3 biophysical growth model-based features	Accuracy = 80.19%	-

Aerts et al. [6]	Lung	Exons 19 and 21 mutations	CT	Memorial Sloan-Kettering Cancer Center, New York City, New York, US	47	-	yes	yes	183	-	AUC = 0.91(	Images
Chen et al. [7]	Lung	mutation	MR	City of Hope Medical Center, Duarte, California, US	110	LOOCV	yes	yes	2,786	Age; sex; ethnicity; history of smoking; histology type; other metastatic sites	AUC = 0.912 Accuracy = 77.7%	-
Hong et al. [8]	Lung	Exons 18, 19, 20, and 21 mutations	CT	The First Hospital of China Medical University, Shenyang, China	140	61*	yes	no	396	Age; sex; history of smoking	AUC = 0.851 [0.750-0.951] c-index = 0.835 [0.825-0.845]	-
Huang et al. [9]	Lung	mutation	CT	The University of Texas MD Anderson Cancer Center, Houston, Texas	46	-	yes	yes	89	-	AUC = 0.88	Images
Jia et al. [10]	Lung	Exons 19 and 21 mutations	CT	Shanghai Chest Hospital, Shanghai, China	345	158*	no	no	440	Age; sex; smoking history; TNM stage	AUC = 0.828 [0.764-0.892]	-
Jiang et al. [11]	Lung	mutation	PET/CT	Shanghai Institute of Medical Imaging, Zhongshan Hospital of Fudan University, Shanghai, China	80	10-CV	yes	no	512	12 semantic features	AUC = 0.953	-
Koyasu et al. [12]	Lung	mutation	PET/CT	TCIA- NSCLC Radiogenomics	138	10-CV	yes	no	Not disclosed	SUVmax; SUVmean; TLG; MTV	AUC = 0.659 Accuracy = 81.2%	Images, ROI
Li et al. [13]	Lung	Exons 18–24 mutations	PET/CT	Tianjin Medical University Cancer Hospital, Tianjin, China	115	10-CV	yes	no	38	SUVmax; SUVmean; SUVpeak; TLG; MTV; age; sex;	AUC = 0.822 Accuracy = 82.65%	-

									smoking status; TNM stage; lesion location		
Li et al. [14]	Lung	mutation	CT	Second Xiangya Hospital of Central South University, Hunan, China	51	10-CV	yes	yes	1,695	-	AUC = 0.83 [0.68-0.92]	Images, ROI
Li et al. [15]	Lung	Exon 19 and 21 mutations	CT	Shanghai Chest Hospital, Shanghai, China	810	200*	yes	no	440	DL prediction; age; sex; smoking history; pathological stage	AUC = 0.834 [0.776–0.892]	-
Li et al. [16]	Lung	Exon 19 and 21 mutations	CT	Shengjing Hospital of China Medical University, Liaoning, China	236	76***	yes	yes	580	Age; sex; tumor grade; lobe; smoking history; intrapulmonary metastasis	AUC = 0.7750-0.7925	-
Liu et al. [17]	Lung	Exons 18-21 mutations	CT	Tianjin Medical University Cancer Institute and Hospital, Tianjin, China	298	bootstrap	yes	no	209	10 tumor spatial location features; age; sex; histological subtype; pathological stage; smoking history	AUC = 0.709 [0.654 - 0.766]	-
Lu et al. [18]	Lung	mutation	CT	The First Hospital of Jilin University, China	83	21*	yes	yes	1,025	45 categorical variables including: age, sex, smoking status, CEA level, vascular	AUC = 0.894	code

										infiltration, visceral pleural infiltration, lymph node metastasis, histological subtype, pathological stage, type of lesion, tumor location, tumor size, tumor necrosis, lobulation, spiculation, vacuolization, etc.		
Mei et al. [19]	Lung	Exon 18-21 mutations	CT	Shenzhen People's Hospital, Guangdong, China	296	-	yes	no	94	Age; sex; smoking status	AUC = 0.75	code
Nair et al. [20]	Lung	Exons 19 and 21 mutations	PET/CT	McGill University Health Centre, 2011 and 2015	50	LOOCV	yes	no	326	-	AUC = 0.8713	-
Rios Velazquez et al. [21]	Lung	mutation	CT	Profile and Harvard-RT (Dana-Farber/Harvard Cancer Center IRB, Boston, MA), Tianjin (Tianjin Medical University IRB, Tianjin, China), Moffitt (IRB Moffitt Cancer Center, Tampa, FL)	353	352***	yes	yes	635	Age; sex; smoking status; ethnicity; clinical stage	AUC = 0.75 [0.69-0.81] Accuracy = 65.0%	Images

Shiri et al. [22]	Lung	Exons 18-21 mutations	PET/CT	TCIA	82	68*	yes	no	109	MTV, SUVmax, SUVpeak, SULmax, SULpeak	AUC = 0.82	Images, ROI, code
Tu et al. [23]	Lung	Exons 18–21 mutations	CT	Changzheng Hospital, Second Military Medical University, Shanghai, China	243	130*	yes	yes	234	Age; sex; smoking status; CEA level; clinical stage; maximum diameter; density; tumor location; interface; shape; lobulation; pleural indentation; spiculation; cusp angle; spine-like process; vacuole sign; cavity sign; air bronchograms ; vascular convergence; pleura thickening; pleural effusion; lymphadenop athy	AUC = 0.818 [0.751-0885] Accuracy = 75.8%	-
Wang et al. [24]	Lung	mutation	CT	Nanjing Medical University Affiliated	41	20*	yes	no	718	78 clinical and pathological features (age,	AUC = 0.697	code

				Cancer Hospital, Nanjing, China						sex, smoking status, histological subtypes, pathological stages, etc.)		
Yang et al. [25]	Lung	Exons 18-21 mutations	CT	The First Affiliated Hospital of Guangzhou Medical University, Guangzhou, China	306	161***	yes	no	1,063	Age; sex; smoking history; CT pattern; histopathological subtype	AUC = 0.779 [0.702-0.856]	code
Yip et al. [26]	Lung	Exons 18–24 mutations	PET	Dana-Farber Cancer Institute, Brigham and Women's Hospital, and Harvard Medical School, Boston, Massachusetts	348	bootstrap	yes	no	68	MTV, SUVmax, SUVpeak, SUVmean, and SUVtot	AUC = 0.67	-
Yip et al. [27]	Lung	mutation	PET	Dana-Farber Cancer Institute, Brigham and Women's Hospital, and Harvard Medical School, Boston, Massachusetts	348	-	yes	yes	66	-	AUC = 0.66	-
Zhang et al. [28]	Lung	Exons 18–21 mutations	PET/CT	The Fourth Hospital of Hebei Medical University, Hebei, China	175	73*	yes	no	92	Age; sex; smoking history; pathological stage; CEA level	AUC = 0.87 [0.79-0.95]	-
Zhang et al. [29]	Lung	Exons 18–21 mutations	CT	West China Hospital, Sichuan, China	140	40*	yes	no	485	Age; sex; smoking status	AUC = 0.8725 Accuracy = 72.5%	-

Zhao et al. [30]	Lung	Exons 18–21 mutations	CT	Huadong Hospital Affiliated to Fudan University, Shanghai, China; TCIA	464 nodules	115 nodules* 37 nodules**	yes	yes	475	DL prediction	AUC = 0.76	Images, ROI
Zhao et al. [31]	Lung	Exon 19 and 21 mutations	CT	Second Xiangya Hospital, Central South University, Changsha, China; Huadong Hospital Affiliated to Fudan University, Shanghai, China	322	315*	yes	yes	475	Age; sex; smoking status; tumor size; tumor location; histological subtype; TNM stage; tumor solidity; tumor margin; tumor type; pleural retraction; bubble lucency; vascular change; bronchiole change; lobulation; spiculation; peripheral emphysema; peripheral fibrosis; pleural effusion	AUC = 0.734	-

Table S 6 An overview of the radiomic studies included for EGFR biomarker. * internal validation; ** external validation; * temporally independent internal validation. Acronyms: epidermal growth factor (EGFR), central nervous system (CNS), computed tomography (CT), magnetic resonance imaging (MRI), positron emission tomography (PET), fluorodeoxyglucose positron emission tomography (FDG-PET), fluoroethyl tyrosine positron emission tomography (FET-PET), perfusion weighted imaging (PWI), magnetic resonance imaging (MRI), diffusion weighted imaging (DWI), amide proton transfer-**

weighted imaging (APTw), max, mean, peak and total standardized uptake value (SUVmax, SUVmean, SUVpeak, SUVtot), total lesion glycolysis (TLG), metabolic tumor volume (MTV), max and peak standardized uptake normalized to lean body mass (SULmax, SULpeak), carcino-embryogenic antigen (CEA), tumor, node and metastasis (TNM), non-small cell lung cancer (NSCLC), The Cancer Imaging Archive (TCIA), deep learning (DL), leave-one-out cross-validation (LOOCV), 3-, 5- and 10-fold cross-validation (3-, 5- and 10-CV), area under the curve (AUC), glioblastoma (GBM), lower-grade glioma (LGG), Karnofsky Performance Status (KPS), The Repository of Molecular Brain Neoplasia Data (REMBRANDT), Visually AccesSAble Rembrandt Images (VASARI), deep learning (DL), deep learning radiomics (DLR), time-to-peak (TTP).

[1] H. Akbari *et al.*, "In vivo evaluation of EGFRvIII mutation in primary glioblastoma patients via complex multiparametric MRI signature," *Neuro-Oncol.*, vol. 20, no. 8, pp. 1068–1079, 05 2018, doi: 10.1093/neuonc/noy033.

[2] Z. A. Binder *et al.*, "Epidermal Growth Factor Receptor Extracellular Domain Mutations in Glioblastoma Present Opportunities for Clinical Imaging and Therapeutic Development," *Cancer Cell*, vol. 34, no. 1, pp. 163-177.e7, 09 2018, doi: 10.1016/j.ccell.2018.06.006.

[3] J. Lee, S. Narang, J. J. Martinez, G. Rao, and A. Rao, "Associating spatial diversity features of radiologically defined tumor habitats with epidermal growth factor receptor driver status and 12-month survival in glioblastoma: methods and preliminary investigation," *J. Med. Imaging Bellingham Wash*, vol. 2, no. 4, p. 041006, Oct. 2015, doi: 10.1117/1.JMI.2.4.041006.

[4] Y. Li *et al.*, "MRI features can predict EGFR expression in lower grade gliomas: A voxel-based radiomic analysis," *Eur. Radiol.*, vol. 28, no. 1, pp. 356–362, Jan. 2018, doi: 10.1007/s00330-017-4964-z.

[5] S. Rathore *et al.*, "Radiomic MRI signature reveals three distinct subtypes of glioblastoma with different clinical and molecular characteristics, offering prognostic value beyond IDH1," *Sci. Rep.*, vol. 8, no. 1, p. 5087, 23 2018, doi: 10.1038/s41598-018-22739-2.

[6] H. J. W. L. Aerts *et al.*, "Defining a Radiomic Response Phenotype: A Pilot Study using targeted therapy in NSCLC," *Sci. Rep.*, vol. 6, p. 33860, 20 2016, doi: 10.1038/srep33860.

[7] B. T. Chen *et al.*, "Radiomic prediction of mutation status based on MR imaging of lung cancer brain metastases," *Magn. Reson. Imaging*, vol. 69, pp. 49–56, Mar. 2020, doi: 10.1016/j.mri.2020.03.002.

[8] D. Hong, K. Xu, L. Zhang, X. Wan, and Y. Guo, "Radiomics Signature as a Predictive Factor for EGFR Mutations in Advanced Lung Adenocarcinoma," *Front. Oncol.*, vol. 10, p. 28, 2020, doi: 10.3389/fonc.2020.00028.

[9] Q. Huang *et al.*, "Interobserver variability in tumor contouring affects the use of radiomics to predict mutational status," *J. Med. Imaging Bellingham Wash*, vol. 5, no. 1, p. 011005, Jan. 2018, doi: 10.1117/1.JMI.5.1.011005.

[10] T.-Y. Jia *et al.*, "Identifying EGFR mutations in lung adenocarcinoma by noninvasive imaging using radiomics features and random forest modeling," *Eur. Radiol.*, vol. 29, no. 9, pp. 4742–4750, Sep. 2019, doi: 10.1007/s00330-019-06024-y.

[11] M. Jiang *et al.*, "Assessing EGFR gene mutation status in non-small cell lung cancer with imaging features from PET/CT," *Nucl. Med. Commun.*, vol. 40, no. 8, pp. 842–849, Aug. 2019, doi: 10.1097/MNM.0000000000001043.

[12] S. Koyasu, M. Nishio, H. Isoda, Y. Nakamoto, and K. Togashi, "Usefulness of gradient tree boosting for predicting histological subtype and EGFR mutation status of non-small cell lung cancer on 18F FDG-PET/CT," *Ann. Nucl. Med.*, vol. 34, no. 1, pp. 49–57, Jan. 2020, doi: 10.1007/s12149-019-01414-0.

[13] X. Li *et al.*, “Predictive Power of a Radiomic Signature Based on 18F-FDG PET/CT Images for EGFR Mutational Status in NSCLC,” *Front. Oncol.*, vol. 9, p. 1062, 2019, doi: 10.3389/fonc.2019.01062.
[14] Y. Li *et al.*, “CT Slice Thickness and Convolution Kernel Affect Performance of a Radiomic Model for Predicting EGFR Status in Non-Small Cell Lung Cancer: A Preliminary Study,” *Sci. Rep.*, vol. 8, no. 1, p. 17913, 17 2018, doi: 10.1038/s41598-018-36421-0.
[15] X.-Y. Li *et al.*, “Detection of epithelial growth factor receptor (EGFR) mutations on CT images of patients with lung adenocarcinoma using radiomics and/or multi-level residual convolutionary neural networks,” *J. Thorac. Dis.*, vol. 10, no. 12, pp. 6624–6635, Dec. 2018, doi: 10.21037/jtd.2018.11.03.
[16] S. Li, C. Ding, H. Zhang, J. Song, and L. Wu, “Radiomics for the prediction of EGFR mutation subtypes in non-small cell lung cancer,” *Med. Phys.*, vol. 46, no. 10, pp. 4545–4552, Oct. 2019, doi: 10.1002/mp.13747.
[17] Y. Liu *et al.*, “Radiomic Features Are Associated With EGFR Mutation Status in Lung Adenocarcinomas,” *Clin. Lung Cancer*, vol. 17, no. 5, pp. 441-448.e6, 2016, doi: 10.1016/j.cllc.2016.02.001.
[18] X. Lu *et al.*, “A novel radiomic nomogram for predicting epidermal growth factor receptor mutation in peripheral lung adenocarcinoma,” *Phys. Med. Biol.*, vol. 65, no. 5, p. 055012, 06 2020, doi: 10.1088/1361-6560/ab6f98.
[19] D. Mei, Y. Luo, Y. Wang, and J. Gong, “CT texture analysis of lung adenocarcinoma: can Radiomic features be surrogate biomarkers for EGFR mutation statuses,” *Cancer Imaging Off. Publ. Int. Cancer Imaging Soc.*, vol. 18, no. 1, p. 52, Dec. 2018, doi: 10.1186/s40644-018-0184-2.
[20] J. K. R. Nair *et al.*, “Radiogenomic Models Using Machine Learning Techniques to Predict EGFR Mutations in Non-Small Cell Lung Cancer,” *Can. Assoc. Radiol. J. J. Assoc. Can. Radiol.*, p. 846537119899526, Feb. 2020, doi: 10.1177/0846537119899526.
[21] E. Rios Velazquez *et al.*, “Somatic Mutations Drive Distinct Imaging Phenotypes in Lung Cancer,” *Cancer Res.*, vol. 77, no. 14, pp. 3922–3930, 15 2017, doi: 10.1158/0008-5472.CAN-17-0122.
[22] I. Shiri *et al.*, “Next-Generation Radiogenomics Sequencing for Prediction of EGFR and KRAS Mutation Status in NSCLC Patients Using Multimodal Imaging and Machine Learning Algorithms,” *Mol. Imaging Biol.*, Mar. 2020, doi: 10.1007/s11307-020-01487-8.
[23] W. Tu *et al.*, “Radiomics signature: A potential and incremental predictor for EGFR mutation status in NSCLC patients, comparison with CT morphology,” *Lung Cancer Amst. Neth.*, vol. 132, pp. 28–35, 2019, doi: 10.1016/j.lungcan.2019.03.025.
[24] X. Wang *et al.*, “Decoding tumor mutation burden and driver mutations in early stage lung adenocarcinoma using CT-based radiomics signature,” *Thorac. Cancer*, vol. 10, no. 10, pp. 1904–1912, Oct. 2019, doi: 10.1111/1759-7714.13163.
[25] X. Yang *et al.*, “Computed Tomography-Based Radiomics Signature: A Potential Indicator of Epidermal Growth Factor Receptor Mutation in Pulmonary Adenocarcinoma Appearing as a Subsolid Nodule,” *The Oncologist*, vol. 24, no. 11, pp. e1156–e1164, Nov. 2019, doi: 10.1634/theoncologist.2018-0706.
[26] S. S. F. Yip *et al.*, “Associations Between Somatic Mutations and Metabolic Imaging Phenotypes in Non-Small Cell Lung Cancer,” *J. Nucl. Med. Off. Publ. Soc. Nucl. Med.*, vol. 58, no. 4, pp. 569–576, 2017, doi: 10.2967/jnumed.116.181826.
[27] S. S. F. Yip, C. Parmar, J. Kim, E. Huynh, R. H. Mak, and H. J. W. L. Aerts, “Impact of experimental design on PET radiomics in predicting somatic mutation status,” *Eur. J. Radiol.*, vol. 97, pp. 8–15, Dec. 2017, doi: 10.1016/j.ejrad.2017.10.009.
[28] J. Zhang *et al.*, “Value of pre-therapy 18F-FDG PET/CT radiomics in predicting EGFR mutation status in patients with non-small cell lung cancer,” *Eur. J. Nucl. Med. Mol. Imaging*, vol. 47, no. 5, pp. 1137–1146, May 2020, doi: 10.1007/s00259-019-04592-1.

[29] L. Zhang *et al.*, “Quantitative Biomarkers for Prediction of Epidermal Growth Factor Receptor Mutation in Non-Small Cell Lung Cancer,” *Transl. Oncol.*, vol. 11, no. 1, pp. 94–101, Feb. 2018, doi: 10.1016/j.tranon.2017.10.012.
[30] W. Zhao *et al.*, “Toward automatic prediction of EGFR mutation status in pulmonary adenocarcinoma with 3D deep learning,” *Cancer Med.*, vol. 8, no. 7, pp. 3532–3543, Jul. 2019, doi: 10.1002/cam4.2233.
[31] W. Zhao *et al.*, “The Potential of Radiomics Nomogram in Non-invasively Prediction of Epidermal Growth Factor Receptor Mutation Status and Subtypes in Lung Adenocarcinoma,” *Front. Oncol.*, vol. 9, p. 1485, 2019, doi: 10.3389/fonc.2019.01485.
