# Supplementary material for: Systematic Review on the Association of Radiomics with Tumor Biological Endpoints
Source: Cancers (Basel). 2021 Jun 16;13(12):3015. doi: 10.3390/cancers13123015 (PMC8234501; doi:10.3390/cancers13123015)
Supplement: Supplementary file 1 [file cancers-13-03015-s001.zip › Supplementary_TableS7_VEGF.pdf]

Study	Tumor Site	Alteration	Modality	Dataset Origin	Training	Validation	Feature Reduction	Feature Robustness	# Radiomic Features	Additional Features	Predictive power Measure = mean [95% confidence interval]	Open Source
Sun et al. [1]	CNS	VEGF expression at < 5%, 6-25%, 26-50% and > 50%	MRI	Beijing Tiantan Hospital, Beijing, China;	160	79*	yes	no	431	-	AUC = 0.702 Accuracy = 72.3%	Images on request
Peng et al. [2]	Liver	expression	US	First Affiliated Hospital of Guangxi Medical University, Nanning, Guangxi, China	39	18*	yes	no	1,076	-	AUC = 0.864 Accuracy = 0.833	-
Chen et al. [3]	Head and Neck	expression	FDG-PET	China Medical University, Taichung City, Taiwan	53	-	no	no	41	SUVmax, MTV, TLGmean; smoking history; tumor origin; TNM stage	Correlation (p < 0.05)	-

Table S 7 An overview of the radiomic studies included for VEGF biomarker. * internal validation. Acronyms: vascular endothelial growth factor (VEGF), central nervous system (CNS), magnetic resonance imaging (MRI), fluorodeoxyglucose positron emission tomography (FDG-PET), ultrasound (US), maximal standardized uptake value (SUVmax), mean total lesion glycolysis (TLGmean), metabolic tumor volume (MTV), tumor, node and metastasis (TNM) , area under the curve (AUC).

[1] Z. Sun *et al.*, “Radiogenomic analysis of vascular endothelial growth factor in patients with diffuse gliomas,” *Cancer Imaging Off. Publ. Int. Cancer Imaging Soc.*, vol. 19, no. 1, p. 68, Oct. 2019, doi: 10.1186/s40644-019-0256-y.

[2] Y.-T. Peng *et al.*, “Preoperative Ultrasound Radiomics Signatures for Noninvasive Evaluation of Biological Characteristics of Intrahepatic Cholangiocarcinoma,” *Acad. Radiol.*, Sep. 2019, doi: 10.1016/j.acra.2019.07.029.

[3] R.-Y. Chen *et al.*, “Associations of Tumor PD-1 Ligands, Immunohistochemical Studies, and Textural Features in 18F-FDG PET in Squamous Cell Carcinoma of the Head and Neck,” *Sci. Rep.*, vol. 8, no. 1, p. 105, 08 2018, doi: 10.1038/s41598-017-18489-2.
