# Supplementary material for: Systematic Review on the Association of Radiomics with Tumor Biological Endpoints
Source: Cancers (Basel). 2021 Jun 16;13(12):3015. doi: 10.3390/cancers13123015 (PMC8234501; doi:10.3390/cancers13123015)
Supplement: Supplementary file 1 [file cancers-13-03015-s001.zip › Supplementary_TableS8_HER2.pdf]

Study	Tumor Site	Alteration	Modality	Dataset Origin	Training	Validation	Feature Reduction	Feature Robustness	# Radiomic Features	Additional Features	Predictive power Measure = mean [95% confidence interval]	Open Source
Antunovic et al. [1]	Breast	positive (IHC 3+) vs. negative (IHC 0 or 1)	FDG-PET/CT	Humanitas Hospital, Milan, Italy	43	-	yes	no	20	MTV, SUV_{mean} and TLG	HER-2: Significant correlation (p = 0.021 - 0.046)	-
Braman et al. [2]	Breast	mutation	PWI	Cleveland Medical Center, Cleveland, Ohio, US; City of Hope Comprehensive Cancer Center, Duarte, California, US; Yale Cancer Center, New Haven, Connecticut, US; Brown University Oncology Research Group, Providence, Rhode Island, US; TCIA/TCGA-BRCA	117	3-CV	yes	no	495	-	AUC = 0.71 [0.63-0.79]	images
Castaldo et al. [3]	Breast	mutation	PWI	TCIA/TCGA-BRCA	55	36*	no	no	36	-	AUC = 0.91<br>Accuracy = 81-88%	images
Fan et al. [4]	Breast	positive (IHC 3+) vs. negative (IHC 0 or 1)	PWI	Zhejiang Cancer Hospital, Hangzhou, China	60	36*	yes	no	65	Age, menopausal status; 29 dynamic features from BPE and the lesion; 9 bilateral differences in BPE	AUC = 0.947	-
Leithner et al. [5]	Breast	mutation	MRI	Memorial Sloan Kettering Cancer Center, New York, USA; Medical University Vienna, Vienna, Austria	91	-	yes	no	352	-	Accuracy = 73.6%	Code

Li et al. [6]	Breast	mutation	PWI	TCGA/TCIA-BRCA	91	LOOCV	yes	no	24	10 kinetic features (maximum contrast enhancement, TTP, uptake rate, washout rate, curve shape index, enhancement at first post-contrast, SER, volume of most enhancing voxels, total rate variation, normalized total rate variation) and 4 enhancement-variance kinetic features (maximum variance of enhancement, TTP, variance increase rate, and variance decrease rate)	AUC = 0.65	images
Li et al.[7]	Breast	mutation	PWI	Cancer Hospital of Liaoning, China	637	LOOCV	yes	no	137	5 kinetic features (standard deviation, mean, maximum value, enhancement rate, absorption rate)	AUC = 0.83<br>Accuracy = 87.0%	-
Monti et al.[8]	Breast	mutation	PWI	Hospital of Moscati, Avellino, Italy; Institute for Hospitalization and Healthcare ***SDN***, Naples, Italy	HER-2: 48<br>Ki-67: 49	bootstrap	yes	no	163	Pharmacokinetic maps	AUC = 0.838<br>Accuracy = 0.785	-
Zhou et al.[9]	Breast	positive (IHC 3+) vs. negative (IHC 0 or 1)	DMG	Henan Provincial People's Hospital, Henan, China	244	62*	yes	no	186	-	AUC = 0.787 [0.673-0.885]<br>Accuracy = 77.00%	-
Zhou et al.[10]	Breast	positive (IHC 3+) vs. negative (IHC 0 or 1)	PWI	The Affiliated Huaian No. 1 People's Hospital of Nanjing Medical University, China	126	5-CV	yes	yes	386	-	AUC = 0.68<br>Accuracy = 0.60	-

Table S 8 An overview of the radiomic studies included for HER-2 biomarker. * internal validation; ** external validation; * temporally independent internal validation. Acronyms: human epidermal growth factor receptor 2 (HER-2), fluorodeoxyglucose positron emission tomography / computed**

tomography (FDG-PET/CT), perfusion weighted imaging (PWI), magnetic resonance imaging (MRI), diffusion weighted imaging (DWI), digital breast tomosynthesis (DBT), digital mammography (DMG), metabolic tumor volume (MTV), mean standardized uptake value (SUVmean), The Cancer Imaging Archive / The Cancer Genome Atlas (TCIA/TCGA), BRCA (BReast invasive CArcinoma), total lesion glycolysis (TLG), background parenchymal enhancement (BPE), signal enhancement ratio (SER), time-to-peak (TTP), leave-one-out cross-validation (LOOCV), 3-, 5- and 10-fold cross-validation (3-, 5- and 10-CV), area under the curve (AUC).

[1] L. Antunovic *et al.*, "[18F]FDG PET/CT features for the molecular characterization of primary breast tumors," *Eur. J. Nucl. Med. Mol. Imaging*, vol. 44, no. 12, pp. 1945–1954, Nov. 2017, doi: 10.1007/s00259-017-3770-9.

[2] N. Braman *et al.*, "Association of Peritumoral Radiomics With Tumor Biology and Pathologic Response to Preoperative Targeted Therapy for HER2 (ERBB2)-Positive Breast Cancer," *JAMA Netw. Open*, vol. 2, no. 4, p. e192561, 05 2019, doi: 10.1001/jamanetworkopen.2019.2561.

[3] R. Castaldo, K. Pane, E. Nicolai, M. Salvatore, and M. Franzese, "The Impact of Normalization Approaches to Automatically Detect Radiogenomic Phenotypes Characterizing Breast Cancer Receptors Status," *Cancers*, vol. 12, no. 2, Feb. 2020, doi: 10.3390/cancers12020518.

[4] M. Fan, H. Li, S. Wang, B. Zheng, J. Zhang, and L. Li, "Radiomic analysis reveals DCE-MRI features for prediction of molecular subtypes of breast cancer," *PloS One*, vol. 12, no. 2, p. e0171683, 2017, doi: 10.1371/journal.pone.0171683.

[5] D. Leithner *et al.*, "Radiomic signatures with contrast-enhanced magnetic resonance imaging for the assessment of breast cancer receptor status and molecular subtypes: initial results," *Breast Cancer Res. BCR*, vol. 21, no. 1, p. 106, 12 2019, doi: 10.1186/s13058-019-1187-z.

[6] H. Li *et al.*, "Quantitative MRI radiomics in the prediction of molecular classifications of breast cancer subtypes in the TCGA/TCIA data set," *NPJ Breast Cancer*, vol. 2, 2016, doi: 10.1038/npjbcancer.2016.12.

[7] W. Li, K. Yu, C. Feng, and D. Zhao, "Molecular Subtypes Recognition of Breast Cancer in Dynamic Contrast-Enhanced Breast Magnetic Resonance Imaging Phenotypes from Radiomics Data," *Comput. Math. Methods Med.*, vol. 2019, p. 6978650, 2019, doi: 10.1155/2019/6978650.

[8] S. Monti *et al.*, "DCE-MRI Pharmacokinetic-Based Phenotyping of Invasive Ductal Carcinoma: A Radiomic Study for Prediction of Histological Outcomes," *Contrast Media Mol. Imaging*, vol. 2018, p. 5076269, 2018, doi: 10.1155/2018/5076269.

[9] J. Zhou *et al.*, "Evaluating the HER-2 status of breast cancer using mammography radiomics features," *Eur. J. Radiol.*, vol. 121, p. 108718, Dec. 2019, doi: 10.1016/j.ejrad.2019.108718.

[10] X. Zhou *et al.*, "Radiomic features of Pk-DCE MRI parameters based on the extensive Tofts model in application of breast cancer," *Phys. Eng. Sci. Med.*, vol. 43, no. 2, pp. 517–524, Jun. 2020, doi: 10.1007/s13246-020-00852-9.
