# Supplementary material for: Systematic Review on the Association of Radiomics with Tumor Biological Endpoints
Source: Cancers (Basel). 2021 Jun 16;13(12):3015. doi: 10.3390/cancers13123015 (PMC8234501; doi:10.3390/cancers13123015)
Supplement: Supplementary file 1 [file cancers-13-03015-s001.zip › Supplementary_TableS9_ALK.pdf]

Study	Tumor Site	Alteration	Modality	Dataset Origin	Training	Validation	Feature Reduction	Feature Robustness	# Radiomic Features	Additional features	Predictive power Measure = mean [95% confidence interval]	Open Source
Chen et al. [1]	Lung	mutation	MR	City of Hope Medical Center, Duarte, California, US	110	LOOCV	yes	yes	2,786	Age; sex; ethnicity; history of smoking; histology type; other metastatic sites	AUC = 0.915 Accuracy = 86.7%	-
Song et al. [2]	Lung	mutation	CT	Peking Union Medical College Hospital, Chinese Academy of Medical Sciences and Peking Union Medical College, November 2015 to October 2018	268	67*	yes	no	1,218	Age; sex; smoking history; smoking index; clinical stage; distal metastasis; pathological invasiveness of tumor; maximum diameter; mean CT attenuation; lesion location; involved lobe; density; margin; cavity; calcification; pleural retraction sign; pleural effusion; pericardial effusion; local lymphadenopathy	AUC = 0.88 [0.77-0.94] Accuracy = 79.0%	Images (partially), code
Yoon et al. [3]	Lung	mutation	PET/CT	Samsung Medical Center, Sungkyunkwan University School of Medicine, Seoul, South Korea	128	10-CV	yes	yes	50	Age; sex; smoking history; stage; SUVmax; tumor solidity; tumor size; tumor location; lymphangitic metastasis; pleural effusion	Sensitivity = 0.73 Specificity = 0.70	-

Table S 9 An overview of the radiomic studies included for ALK biomarker. * internal validation. Acronyms: anaplastic lymphoma kinase (ALK), computed tomography (CT), magnetic resonance imaging (MRI), positron emission tomography (PET), max standardized uptake value (SUVmax, leave-one-out cross-validation (LOOCV), 10-fold cross-validation (10-CV), area under the curve (AUC).

[1] B. T. Chen *et al.*, "Radiomic prediction of mutation status based on MR imaging of lung cancer brain metastases," *Magn. Reson. Imaging*, vol. 69, pp. 49–56, Mar. 2020, doi: 10.1016/j.mri.2020.03.002.

[2] L. Song *et al.*, "Clinical, Conventional CT and Radiomic Feature-Based Machine Learning Models for Predicting ALK Rearrangement Status in Lung Adenocarcinoma Patients," *Front. Oncol.*, vol. 10, Mar. 2020, doi: 10.3389/fonc.2020.00369.

[3] H. J. Yoon *et al.*, "Decoding Tumor Phenotypes for ALK, ROS1, and RET Fusions in Lung Adenocarcinoma Using a Radiomics Approach," *Medicine (Baltimore)*, vol. 94, no. 41, p. e1753, Oct. 2015, doi: 10.1097/MD.0000000000001753.
